# Fission Yeast Shelterin Regulates DNA Polymerases and Rad3^ATR^ Kinase to Limit Telomere Extension

**DOI:** 10.1371/journal.pgen.1003936

**Published:** 2013-11-07

**Authors:** Ya-Ting Chang, Bettina A. Moser, Toru M. Nakamura

**Affiliations:** Department of Biochemistry and Molecular Genetics, College of Medicine, University of Illinois at Chicago, Chicago, Illinois, United States of America; EPFL Lausanne, Switzerland

## Abstract

Studies in fission yeast have previously identified evolutionarily conserved shelterin and Stn1-Ten1 complexes, and established Rad3^ATR^/Tel1^ATM^-dependent phosphorylation of the shelterin subunit Ccq1 at Thr93 as the critical post-translational modification for telomerase recruitment to telomeres. Furthermore, shelterin subunits Poz1, Rap1 and Taz1 have been identified as negative regulators of Thr93 phosphorylation and telomerase recruitment. However, it remained unclear how telomere maintenance is dynamically regulated during the cell cycle. Thus, we investigated how loss of Poz1, Rap1 and Taz1 affects cell cycle regulation of Ccq1 Thr93 phosphorylation and telomere association of telomerase (Trt1^TERT^), DNA polymerases, Replication Protein A (RPA) complex, Rad3^ATR^-Rad26^ATRIP^ checkpoint kinase complex, Tel1^ATM^ kinase, shelterin subunits (Tpz1, Ccq1 and Poz1) and Stn1. We further investigated how telomere shortening, caused by *trt1Δ* or catalytically dead Trt1-D743A, affects cell cycle-regulated telomere association of telomerase and DNA polymerases. These analyses established that fission yeast shelterin maintains telomere length homeostasis by coordinating the differential arrival of leading (Polε) and lagging (Polα) strand DNA polymerases at telomeres to modulate Rad3^ATR^ association, Ccq1 Thr93 phosphorylation and telomerase recruitment.

## Author Summary

Stable maintenance of telomeres is critical to maintain a stable genome and to prevent accumulation of undesired mutations that may lead to formation of tumors. Telomere dysfunction can also lead to premature aging due to depletion of the stem cell population, highlighting the importance of understanding the regulatory mechanisms that ensure stable telomere maintenance. Based on careful analysis of cell cycle-regulated changes in telomere association of telomerase, DNA polymerases, Replication Protein A, checkpoint kinases, telomere protection complex shelterin, and Stn1-Ten1 complex, we will provide here a new and dynamic model of telomere length regulation in fission yeast, which suggests that shelterin-dependent regulation of differential arrival of leading and lagging strand DNA polymerase at telomeres is responsible for modulating Rad3^ATR^ checkpoint kinase accumulation and Rad3^ATR^-dependent phosphorylation of shelterin subunit Ccq1 to control telomerase recruitment to telomeres.

## Introduction

In eukaryotic cells, dynamic cell cycle-regulated protein-DNA complexes formed at telomeres play key roles in the maintenance of genome stability [1], [2]. Telomeric DNA, consisting of repetitive GT-rich sequences, is extended by telomerase to overcome loss of telomeric DNA due to the inability of replicative DNA polymerases to fully replicate ends of linear DNA molecules [3]. While telomeric DNA is mostly double-stranded, telomeres terminate with a single-stranded GT-rich 3′ overhang, known as G-tail. Cells have evolved distinct proteins that specifically recognize either double-stranded or single-stranded telomeric DNA [4].

In mammalian cells, double-stranded DNA (dsDNA)-specific telomere binding proteins are encoded by TRF1 and TRF2 and a single-stranded DNA (ssDNA)-specific telomere binding protein is encoded by POT1, and together with RAP1, TIN2 and TPP1, they form a telomere protection complex known as “shelterin” [4]. Mutations that affect shelterin or telomerase function in mammalian cells could lead to diseases that show premature aging due to depletion of the stem cell population, highlighting the importance to understand the regulatory mechanisms that ensure stable telomere maintenance [5].

**Figure 1:**
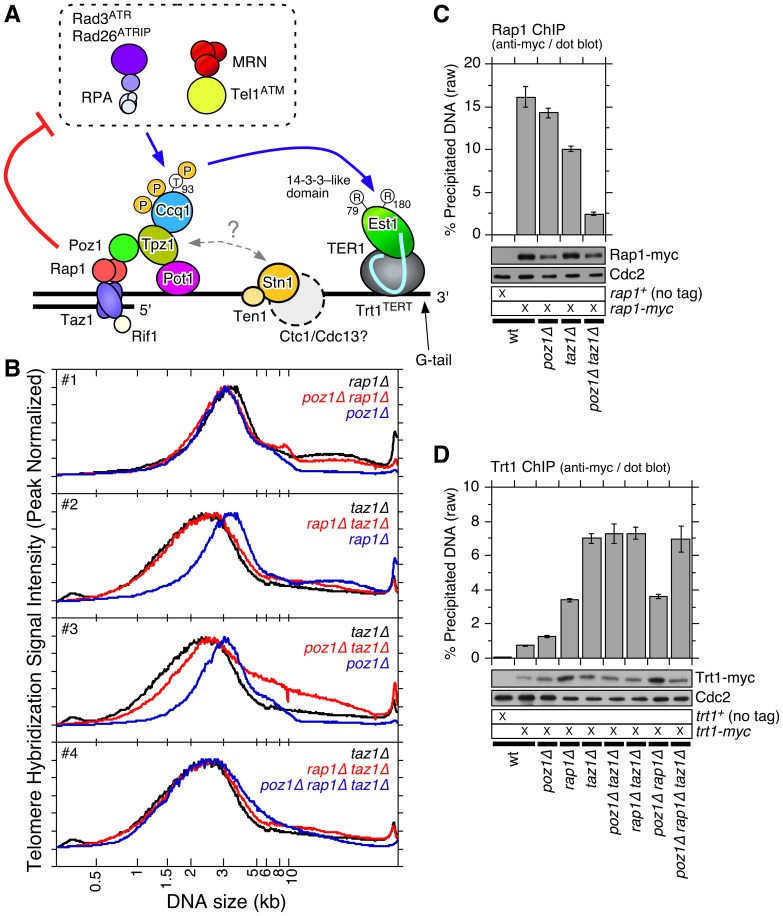
Epistasis analysis of *poz1Δ*, *rap1Δ*, and *taz1Δ* cells. (**A**) Schematic model of telomere maintenance regulation in fission yeast. (**B**) Quantification of telomere length distribution for Southern blot analysis shown in Figure S1A. (**C**, **D**) Recruitment of Rap1 (C) and Trt1^TERT^ (D) to telomeres, monitored by dot blot-based ChIP assays. Compared to wt cells, Rap1 showed statistically significant reductions in binding to telomeres in *taz1Δ* (p = 2.5×10^−3^) and *poz1Δ taz1Δ* (p = 2.6×10^−5^) cells. Rap1 still showed statistically significant binding to telomeres even in *poz1Δ taz1Δ* cells compared to no tag control strain (p = 1.4×10^−5^). Error bars correspond to the standard error of the mean (SEM) from multiple independent experiments. Anti-myc western blot analysis indicated comparable expression levels in different genetic backgrounds. Cdc2 western blot served as a loading control.

Identification of a telomere protection complex that closely resembles mammalian shelterin [6], coupled with the amenability to detailed genetic and molecular analysis, have made fission yeast *Schizosaccharomyces pombe* an attractive model organism to study telomere maintenance [7]. The shelterin complex in fission yeast consists of Taz1 (TRF1/TRF2 ortholog) that specifically recognizes double-stranded telomeres, the G-tail binding protein Pot1, Tpz1 (TPP1 ortholog), Rap1, Poz1 and Ccq1. In addition, Rif1 also interacts with Taz1 [8]. Similar to the way TIN2 and TPP1 connect TRF1/TRF2 to POT1 in mammalian shelterin, Rap1, Poz1 and Tpz1 connect Taz1 to Pot1 (Figure 1A). Ccq1, which directly interacts with both Tpz1 and the telomerase regulatory subunit Est1, plays a critical role in both recruitment of telomerase and attenuation of Rad3^ATR^-dependent DNA damage checkpoint responses [6], [9], [10]. Checkpoint kinases Rad3^ATR^ and Tel1^ATM^ are redundantly required for telomere maintenance and telomerase recruitment [11], [12], since the interaction between Ccq1 and the 14-3-3-like domain of Est1 is facilitated by Rad3^ATR^/Tel1^ATM^-dependent phosphorylation of Ccq1 on Thr93 [10], [13]. Poz1, Rap1, and Taz1 are necessary to limit Ccq1 phosphorylation and uncontrolled telomere extension by telomerase [10], but exactly how they prevent Rad3^ATR^/Tel1^ATM^-dependent phosphorylation of Ccq1 has not yet been established.

In addition to shelterin, another evolutionarily conserved ssDNA binding complex, known as CST (CTC1-STN1-TEN1 in mammalian cells and Cdc13-Stn1-Ten1 in budding yeast *Saccharomyces cerevisiae*), has been implicated in telomere maintenance [14]–[16]. CST interacts with the primase-DNA polymerase α complex [17]–[19], and regulates G-tail length by promoting lagging strand synthesis at telomeres [20]–[22]. Furthermore, CST may inhibit telomerase activity by interacting with TPP1 [23]. While a Cdc13/CTC1-like protein has not yet been identified in fission yeast (Figure 1A), deleting Stn1 or Ten1 resulted in immediate telomere fusion, highlighting the critical role of the Stn1-Ten1 complex in telomere maintenance [24].

Using Chromatin immunoprecipitation (ChIP) assays, we have previously established cell cycle-regulated changes in telomere association of telomere-specific proteins (telomerase catalytic subunit Trt1^TERT^, Taz1, Rap1, Pot1 and Stn1), DNA replication proteins (DNA polymerases, MCM and RPA), the checkpoint protein Rad26^ATRIP^ (a regulatory subunit of checkpoint kinase Rad3^ATR^) and DNA repair protein Nbs1 (a subunit of Mre11-Rad50-Nbs1 complex) in fission yeast [25]. Unexpectedly, the leading strand DNA polymerase Polε arrived at telomeres significantly earlier than the lagging strand DNA polymerases Polα and Polδ in late S-phase. Temporal recruitment of RPA and Rad26^ATRIP^ matched the arrival of Polε, while recruitment of Trt1^TERT^, Pot1 and Stn1 matched the arrival of Polα. However, it has not yet been established if the delayed arrival of Polα/Polδ represents a C-strand fill-in reaction after extension of the G-strand by telomerase, or if it might be part of the regulatory mechanism that controls recruitment of telomerase by regulating Rad3^ATR^/Tel1^ATM^ accumulation and Ccq1 Thr93 phosphorylation. While previous studies have established that Taz1 and mammalian TRF1 contribute to efficient replication of telomeric repeats [26], [27], very little is known how the loss of Taz1 or TRF1 affects behaviors of replicative DNA polymerases at telomeres. In addition, it is currently unknown how cell cycle-regulated dynamic binding patterns of checkpoint kinases, shelterin and CST are affected by challenges posed by replicating highly extended telomeric repeats as found in *poz1Δ*, *rap1Δ*, and *taz1Δ* cells.

Therefore, we investigated how loss of the shelterin subunits Poz1, Rap1 and Taz1 affects cell cycle-regulated recruitment timing of telomerase catalytic subunit Trt1^TERT^, DNA polymerases (Polα and Polε), the Replication Protein A (RPA) complex subunit Rad11, the Rad3^ATR^-Rad26^ATRIP^ checkpoint kinase complex, Tel1^ATM^ kinase, shelterin subunits (Tpz1 Ccq1 and Poz1), and Stn1. In addition, we investigated how telomere shortening, caused either by deletion of Trt1^TERT^ or introduction of catalytically dead Trt1^TERT^, affected cell cycle-regulated telomere association of telomerase and DNA polymerases. Our detailed ChIP analyses provide new insights into the dynamic coordination of DNA replication, DNA damage kinase recruitment, and telomerase recruitment in fission yeast.

## Results

### Epistasis analysis of telomerase inhibitors Poz1, Rap1 and Taz1

To better understand how Poz1, Rap1 and Taz1 function together in telomere maintenance, we performed epistasis analysis among single, double and triple deletion mutant cells for telomere length, cold sensitivity, protection of telomeres against telomere fusion in G_1_ arrested cells, and recruitment of Trt1^TERT^ to telomeres [6], [8], [28], [29]. Telomere length distribution of *poz1Δ*, *rap1Δ* and *poz1Δ rap1Δ* cells closely resembled one another (Figures S1A and 1B #1), suggesting that *poz1Δ* and *rap1Δ* cause similar defect(s) in telomere length regulation. The distribution of telomere length was broader and skewed toward shorter telomeres in *taz1Δ* cells than *rap1Δ* or *poz1Δ* cells (Figure 1B #2-3), and *rap1Δ taz1Δ* and *poz1Δ rap1Δ taz1Δ* cells showed identical telomere length distributions as *taz1Δ* cells (Figure 1B #4), suggesting that Taz1 carries out both Poz1/Rap1-dependent and -independent roles in telomere length regulation. Interestingly, since telomere length distribution in *poz1Δ taz1Δ* was much broader than in *poz1Δ rap1Δ taz1Δ* cells (Figure 1B #3-4), it appears that Rap1 could also affect telomere length independently of Poz1 and Taz1. In support for such independent function, Rap1 binding to telomeres was significantly reduced but not entirely eliminated in *poz1Δ taz1Δ* cells (Figure 1C). Previously, we have also found that Rap1 contributes to recombination-based telomere maintenance independently of Taz1 and Poz1 [30].

We also found that *poz1Δ* and *taz1Δ* cells, but not *rap1Δ* cells, show reduced cell growth at lower temperature (Figure S1B). Cold sensitivity of *taz1Δ* cells [28] was more severe than *poz1Δ* cells, and *poz1Δ taz1Δ* cells were more sensitive than *taz1Δ* cells. Interestingly, while *rap1Δ taz1Δ* cells showed the most severe cold sensitivity among all mutant combinations tested, cold sensitivity of *poz1Δ rap1Δ taz1Δ* cells was milder, suggesting that the presence of Poz1 in *rap1Δ taz1Δ* cells is detrimental to cell growth at low temperature. In addition, *rap1Δ* and *taz1Δ* cells, but not *poz1Δ* cells, showed telomere-telomere fusion [28], [31], [32] when cells are grown in low nitrogen media to arrest cells in G_1_ (Figure S1C). Among double and triple mutant cells, all cells that lack Rap1 and/or Taz1 underwent telomere fusion. Thus, only Rap1 and Taz1 (but not Poz1) are involved in protection of telomeres against fusions in G_1_ arrested cells.

Based on ChIP analysis utilizing the hybridization of a telomeric probe to dot blotted samples, we found that Trt1^TERT^ showed progressive increase in telomere association in the order of *poz1Δ*, *rap1Δ* and *taz1Δ* cells [10] (Figure 1D). Further analysis of double and triple mutant cells revealed that *poz1Δ rap1Δ* cells have similar levels of Trt1^TERT^ binding as *rap1Δ* cells, and *poz1Δ taz1Δ*, *rap1Δ taz1Δ* and *poz1Δ rap1Δ taz1Δ* cells have similar levels of Trt1^TERT^ binding as *taz1Δ* cells. Thus, regarding its inability to limit telomerase binding to telomeres, *taz1Δ* is epistatic over *rap1Δ* or *poz1Δ*, and *rap1Δ* is epistatic over *poz1Δ*.

### Changes in cell cycle-regulated telomere association of Trt1^TERT^ in wt, *poz1Δ*, *rap1Δ*, and *taz1Δ* cells

Trt1^TERT^ binding to telomeres is cell cycle-regulated, and maximal association of Trt1^TERT^ occurs in late-S phase when telomeres are replicated [25]. To better understand the roles of Poz1, Rap1 and Taz1 in limiting Trt1^TERT^ binding to telomeres, we examined changes in Trt1^TERT^ association in *poz1Δ*, *rap1Δ* and *taz1Δ* cells by ChIP using *cdc25-22* synchronized cell cultures. Since our asynchronous ChIP analysis indicated that Trt1^TERT^ recruitment to telomeres is similar in single and double/triple mutant cells, we limited our cell cycle ChIP analysis to single mutant cells. After incubating *cdc25-22* cells at non-permissive temperature (36°C) for 3 hours, late G_2_-phase arrested cells were shifted to permissive temperature (25°C) for synchronous cell cycle re-entry, and samples were collected every 20 minutes and processed for ChIP analysis.

In previous cell cycle ChIP analyses, we utilized quantitative real-time PCR with primers that amplify a unique sub-telomeric DNA sequence directly adjacent to telomeric repeats [12], [25]. Since wild-type (wt) telomeres are only ∼300 bp and the size of DNA fragments after sonication is estimated to be in the 0.5∼1 kb range, the use of sub-telomeric primers provides a convenient mean to determine the association of various factors to telomeres. However, since *poz1Δ*, *rap1Δ* and *taz1Δ* cells carry much longer telomeres, sub-telomeric PCR primer pairs would be too distant from the actual chromosome ends, and thus protein binding to telomeres had to be monitored using dot blotted samples and utilizing telomeric repeat DNA as hybridization probe [10].

For analysis of dot blot-based ChIP assays, we processed raw data (% precipitated DNA) in two different ways. First, to compare overall temporal binding patterns, we normalized ChIP data to the peak of binding within the first complete cell cycle (40–200 min) after release from the G_2_ arrest. Second, we attempted to obtain an approximate fold-increase in protein association “per chromosome end” by correcting for changes in telomeric tract length (Materials and Methods and Table S1). This correction was necessary as raw % precipitated DNA values reflect the density of a given protein within the telomeric tract, and thus significantly underestimate the actual increase in protein binding at chromosome ends for cells carrying long telomeric repeat tracts. Telomere length corrected ChIP data were normalized to values from wt cells for asynchronous ChIP assays, and normalized to the peak binding values of wt cells in late S/G_2_-phase for cell cycle ChIP assays. (See Figures S2 for telomere length correction of Trt1^TERT^ asynchronous ChIP data as example.)

Based on changes in % septated cells, *poz1Δ*, *rap1Δ* and *taz1Δ* cells showed similar re-entries into cell cycle as wt cells (Figure S3C), with the first S-phase occurring 60–140 min and the second S-phase starting 200–220 min after the temperature shift. BrdU incorporation data indicated that telomeres in wt, *poz1Δ* and *rap1Δ* cells are replicated in late S-phase (100–140 min after the temperature shift), while replication of telomeres in *taz1Δ* cells occurred much earlier (60–100 min after the temperature shift) (Figure S4B). Furthermore, hydroxyurea (HU) treatment completely abolished telomere replication in wt, *poz1Δ* and *rap1Δ* cells, but not in *taz1Δ* cells. These data are consistent with previous findings that Taz1 is required to enforce late S-phase replication at telomeres [33], [34].

**Figure 2:**
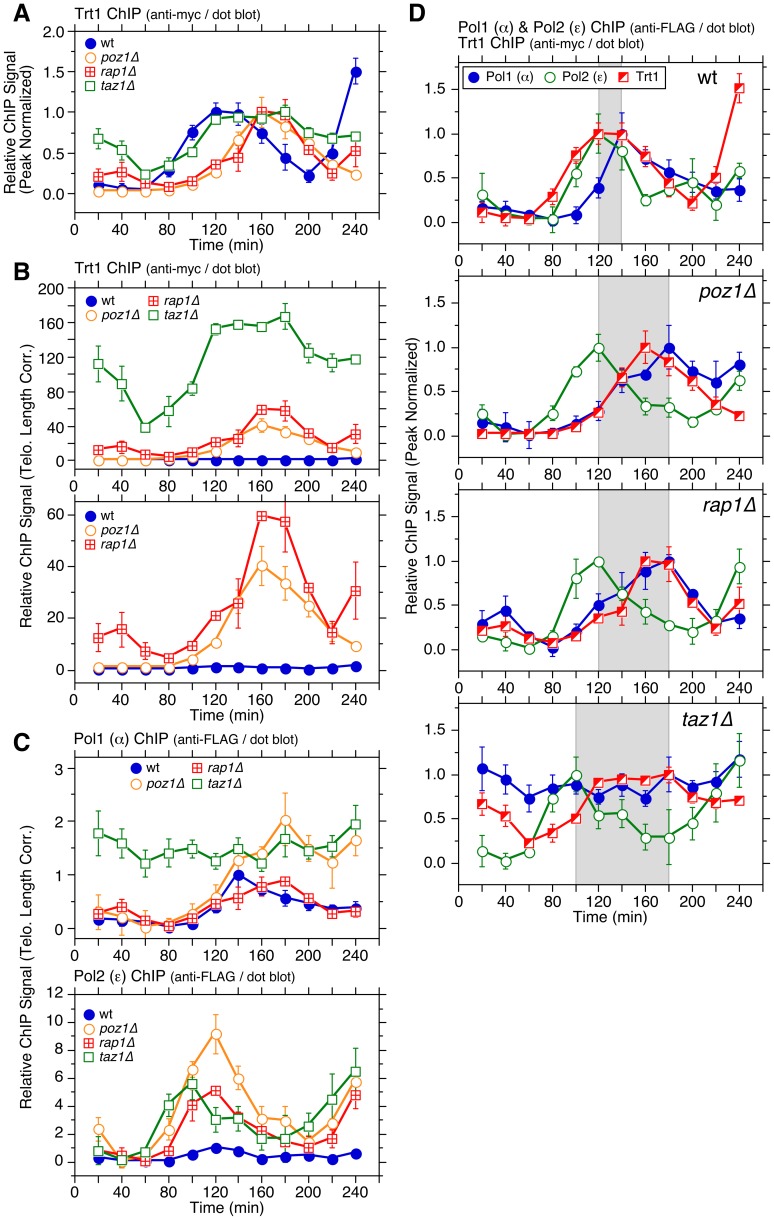
Cell cycle ChIP analysis to monitor association of Trt1^TERT^ and DNA polymerases with telomeres. (**A**) Peak normalized and (**B**) telomere length adjusted ChIP data for Trt1^TERT^ in wt, *poz1Δ*, *rap1Δ*, and *taz1Δ* cells. For (A), *poz1Δ* and *rap1Δ* cells showed statistically significant increases in Trt1^TERT^ association (p<0.03) compared to wt cells at 100 and 120 min. Raw ChIP data and % septated cells to monitor cell cycle progression are shown in Figure S3. (**C**) Telomere length adjusted ChIP data for Pol1 (α) and Pol2 (ε) in wt, *poz1Δ*, *rap1Δ*, and *taz1Δ* cells. Peak normalized ChIP data, raw ChIP data, and % septated cells to monitor cell cycle progression are shown in Figure S5. Anti-FLAG western blot analysis indicated comparable expression levels in different genetic backgrounds (Figure S5G). (**D**) Comparison of peak normalized ChIP data for Pol1 (α), Pol2 (ε) and Trt1^TERT^. Shaded area in wt, *poz1Δ*, and *rap1Δ* graphs corresponds to the interval between Pol2 (ε) and Pol1 (α) peaks, while shaded area in *taz1Δ* graph corresponds to the interval between Pol2 (ε) peak and the last time point of Trt1^TERT^ at peak binding before it shows reduction. Error bars correspond to SEM.

Consistent with our previous analysis [25], Trt1^TERT^ showed maximal binding to telomeres in late S-phase (120–140 min) in wt cells (Figure 2A). In *poz1Δ* and *rap1Δ* cells, Trt1^TERT^ showed nearly identical cell cycle-regulated association patterns with a substantial delay in maximal binding (160–180 min) (Figure 2A). In agreement with a recent report [34], we found that Trt1^TERT^ is bound to telomeres throughout the cell cycle in *taz1Δ* cells with much broader and persistent maximal binding at 120–180 min (Figures 2B and S3A–B). Consistent with asynchronous ChIP data, relative peak binding values (telomere length corrected) for Trt1^TERT^ increased in the order of *poz1Δ* (∼40-fold), *rap1Δ* (∼59-fold) and *taz1Δ* (∼167-fold) over wt cells (Figure 2B).

### Poz1, Rap1 and Taz1 control cell cycle-dependent association of DNA polymerases to telomeres

Real-time PCR-based ChIP assays have previously established that the leading strand DNA polymerase Polε arrives at telomeres significantly earlier than the lagging strand DNA polymerases Polα and Polδ, and that the timing of maximal Trt1^TERT^ association matches more closely to that of Polα and Polδ (∼140 min) than Polε (∼120 min) [25]. Our dot blot-based ChIP re-confirmed the differential timing in peak association for Polα and Polε in wt cells (Figures 2C and S5). In *poz1Δ* and *rap1Δ* cells, binding of Polα was delayed ∼40 min without affecting the temporal binding pattern of Polε. The delay of Polα appears to be restricted to telomeres, as the timing of Polα association with *ars2004* (early replication origin) was similar among wt, *poz1Δ* and *rap1Δ* cells (Figure S4C). Overall, the cell cycle-regulated association patterns for both polymerases were nearly identical in *poz1Δ* and *rap1Δ* cells, but both Polα and Polε showed increased association with telomeres in *poz1Δ* cells than *rap1Δ* cells (Figures 2C and S5A–B).

In *taz1Δ* cells, the difference in telomere binding patterns for the leading and lagging strand DNA polymerases was more dramatic. As expected based on the fact that *taz1Δ* cells replicate telomeres much earlier in S-phase [33] (Figure S4B), Polε was recruited to telomeres earlier (peak binding ∼100 min) (Figure S5B). When corrected for telomere length, we found a ∼6 fold increase in peak ChIP precipitation for Polε in *taz1Δ* cells over wt cells (Figure 2C). Surprisingly, Polα was constitutively bound to telomeres throughout the cell cycle in *taz1Δ* cells at ∼1.5 fold above the peak binding in wt cells (Figures 2C and S5A). On the other hand, overall cell cycle progression (Figure S5E–F) and association timing for Polα to *ars2004* (Figure S4C) were not affected in *taz1Δ* cells. Taken together, we concluded that Poz1 and Rap1 are required primarily to maintain timely recruitment of Polα to telomeres, and Taz1 is required to both (1) delay arrival of Polε to enforce late S-phase replication of telomeres and (2) enforce cell cycle-regulated association of Polα with telomeres.

### Comparison of cell cycle-regulated association patterns for telomerase and DNA polymerases

Previous ChIP analysis using real-time PCR found largely overlapping temporal association patterns for the telomerase catalytic subunit Trt1^TERT^ and Polα with both showing maximal binding at ∼140 min in wt cells [25]. However, the initial increase in detectable binding to telomeres was earlier for Trt1^TERT^ (∼80 min) than Polα (∼100 min) and treatment with HU caused much greater inhibition of Polα and Polε binding than Trt1^TERT^, suggesting that Trt1^TERT^ binding could occur prior to the arrival of replicative polymerases at telomeres [25].

With dot blot-based ChIP analysis, the overall binding pattern for Trt1^TERT^ was broader than in our previous analysis (Figure 2A) [25]. Thus, when data for Trt1^TERT^, Polα and Polε were plotted together (Figure 2D), the increase in Trt1^TERT^ binding prior to arrival of Polα became more evident. On the other hand, reductions in the binding of Trt1^TERT^ and Polα in G_2_/M phase occurred with very similar timing. In *poz1Δ* and *rap1Δ* cells, the peak of Trt1^TERT^ recruitment was dramatically delayed compared to Polε and its overall temporal association pattern largely overlapped with Polα (Figure 2D).

However, the initial increase in Trt1^TERT^ binding to telomeres occurred with similar timing as Polε in *poz1Δ*, *rap1Δ* or *taz1Δ* cells (Figure S6A), and the amount of Trt1^TERT^ binding was already significantly increased in early S-phase (80–100 min) and further elevated during late S/G_2_-phases (160–180 min) in these deletion mutants (Figure 2B). Thus, the delay in peak binding of Trt1^TERT^ in *poz1Δ* and *rapΔ* cells is caused primarily by the massive increase in Trt1^TERT^ binding during late S/G_2_-phases. Likewise, the broad and persistent binding of Trt1^TERT^ in *taz1Δ* cells can be attributed to both a massive increase in early S-phase and persistent binding in late S/G_2_-phases. Taken together, we thus concluded that Trt1^TERT^ binding to telomeres occurs around the time when Polε arrives at telomeres, and that its binding is massively increased throughout S-phase in cells that lack Poz1, Rap1 or Taz1, accompanied by delayed (*poz1Δ* and *rap1Δ*) or persistent (*taz1Δ*) binding of Polα.

### Poz1, Rap1 and Taz1 prevent accumulation of the Rad3^ATR^-Rad26^ATRIP^ complex at telomeres

**Figure 3:**
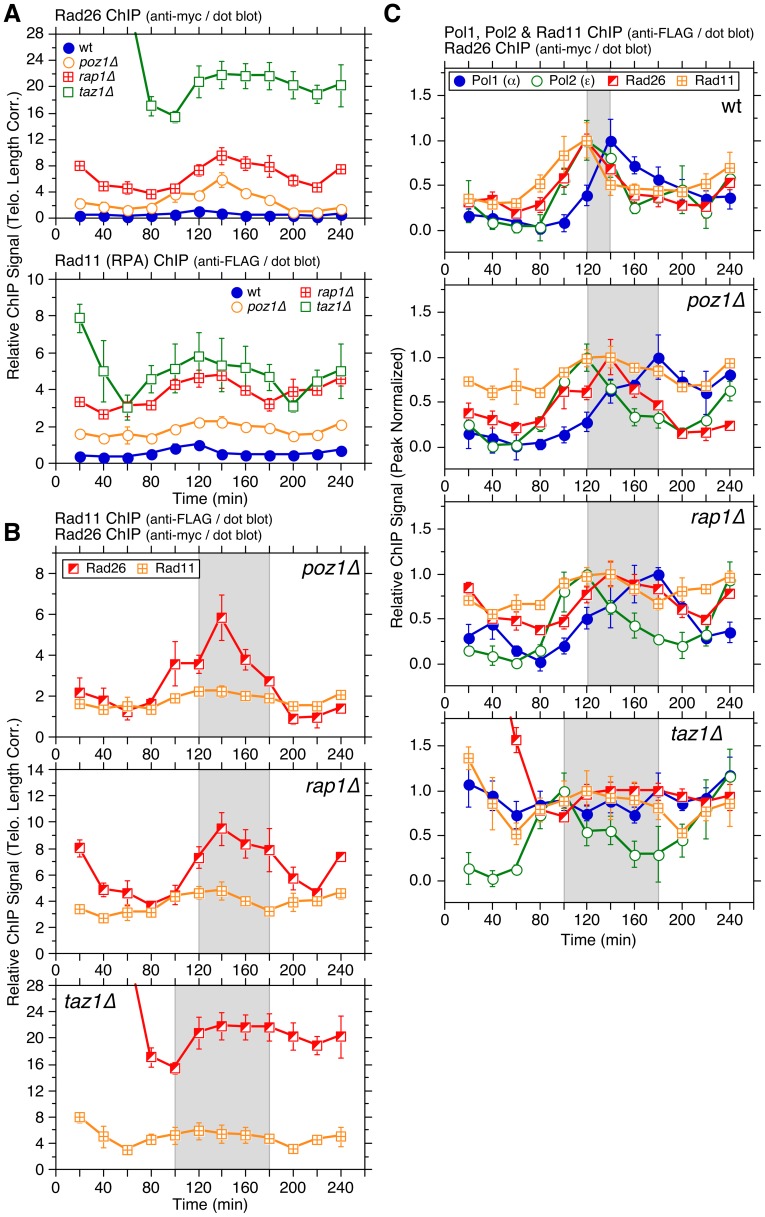
Cell cycle ChIP analysis to monitor association of Rad26^ATRIP^ and Rad11^RPA^ with telomeres. (**A**) Telomere length adjusted ChIP data for Rad26^ATRIP^ and Rad11^RPA^ in wt, *poz1Δ*, *rap1Δ*, and *taz1Δ* cells. Peak normalized ChIP data, raw ChIP data, and % septated cells to monitor cell cycle progression are shown in Figure S11. Anti-myc and anti-FLAG western blot analysis indicated comparable expression levels in different genetic backgrounds for Rad26 and Rad11, respectively (Figure S8D). (**B**) Comparison of telomere length adjusted ChIP data for Rad26^ATRIP^ and Rad11^RPA^ in *poz1Δ*, *rap1Δ* or *taz1Δ* cells. (**C**) Comparison of peak normalized ChIP data for Pol1, Pol2, Rad26^ATRIP^, and Rad11^RPA^. For (B) and (C), see Figure 2 legend for explanation of shaded areas. Error bars correspond to SEM.

The differential arrival of leading and lagging strand DNA polymerases could temporarily create extended ssDNA at telomeres that are then replicated by the lagging strand polymerase. Indeed, both the largest subunit of the ssDNA binding complex RPA (Rad11) and the checkpoint kinase regulatory subunit Rad26 (ATRIP ortholog) showed increased binding to telomeres as the leading strand DNA polymerase (Polε) arrives and reduced binding as the lagging strand DNA polymerases (Polα and Polδ) arrive at telomeres [25] (Figure 3C wt). Since Polα association is even more delayed in *poz1Δ* and *rap1Δ* cells and severely deregulated in *taz1Δ* cells (Figure 2C–D), we predicted that both the Rad3^ATR^-Rad26^ATRIP^ complex and Rad11^RPA^ to increase in telomere association upon loss of Poz1, Rap1 or Taz1.

Indeed, asynchronous ChIP assays found that Rad3^ATR^, Rad26^ATRIP^ and Rad11^RPA^ all show a significant increase in binding to telomeres in *poz1Δ*, *rap1Δ* and *taz1Δ* cells (Figures S7A and S8). The extent of increase was much greater for Trt1^TERT^ and Rad26^ATRIP^ than Rad3^ATR^ and Rad11^RPA^, but all showed a much greater degree of binding increase to telomeres than shelterin subunits (Tpz1, Ccq1 and Poz1) or Stn1 (Figures S7B and S9). In contrast to Rad3^ATR^-Rad26^ATRIP^, Tel1^ATM^ kinase did not show much increase in telomere association upon elimination of Poz1, Rap1 or Taz1, even though Rad3^ATR^ and Tel1^ATM^ play redundant role(s) in telomere protection and telomerase recruitment (Figure S10). These data are consistent with previous conclusions that Rad3^ATR^ plays a much greater role in regulation of telomere length and Ccq1 phosphorylation than Tel1^ATM^ in fission yeast [10], [12], [13].

While Rad26^ATRIP^ and Rad11^RPA^ association increased throughout the cell cycle in *poz1Δ* and *rap1Δ* cells compared to wt, the most noticeable change was their increased and persistent binding during the extended time period (80–200 min) between the arrival of Polε and dissociation of Polα (Figures 3 and S11A–B). While increases in telomere binding during S-phase were more dramatic for Rad26^ATRIP^ than Rad11^RPA^ (Figure 3B), both proteins showed significantly higher binding to telomeres in *rap1Δ* than in *poz1Δ* cells (Figure 3A), consistent with asynchronous ChIP data (Figure S7A) and our previous findings that *rap1Δ* cells show stronger induction of Ccq1 Thr93 phosphorylation and increased binding of Trt1^TERT^ than *poz1Δ* cells [10].

For *taz1Δ* cells, both Rad26^ATRIP^ and Rad11^RPA^ showed their strongest binding to telomeres immediately after release from *cdc25-22* induced G_2_ arrest (Figures 3A and S11A–D), suggesting that prolonged arrest in G_2_ might cause continued resection of telomeric ends and much higher levels of Rad3^ATR^-Rad26^ATRIP^ and Rad11^RPA^ accumulation specifically in *taz1Δ* cells. Nevertheless, both Rad26^ATRIP^ and Rad11^RPA^ showed significant reduction in telomere association as cells completed mitosis (∼80 min), increased and persistent binding during S/G_2_-phase, and slight reduction in binding in late G_2_/M-phase (Figures 3 and S11A–D). Thus, despite the lack of any observable cell cycle regulation for Polα association with telomeres in *taz1Δ* cells, there must be some changes at *taz1Δ* telomeres that allow a slight reduction in association of the Rad3^ATR^-Rad26^ATRIP^ kinase complex and RPA in late G_2_/M-phase.

### Ccq1 Thr93 phosphorylation during cell cycle in wt, *rap1Δ* and *taz1Δ* cells

Phosphorylation of Ccq1 Thr93 by Rad3^ATR^ and Tel1^ATM^ kinases is important for telomerase recruitment in fission yeast [10], [13]. Since Ccq1 is hyper-phosphorylated in *poz1Δ*, *rap1Δ*, or *taz1Δ* cells at Thr93 and additional unidentified phosphorylation sites [10], we next examined how Ccq1 phosphorylation is regulated during cell cycle.

**Figure 4:**
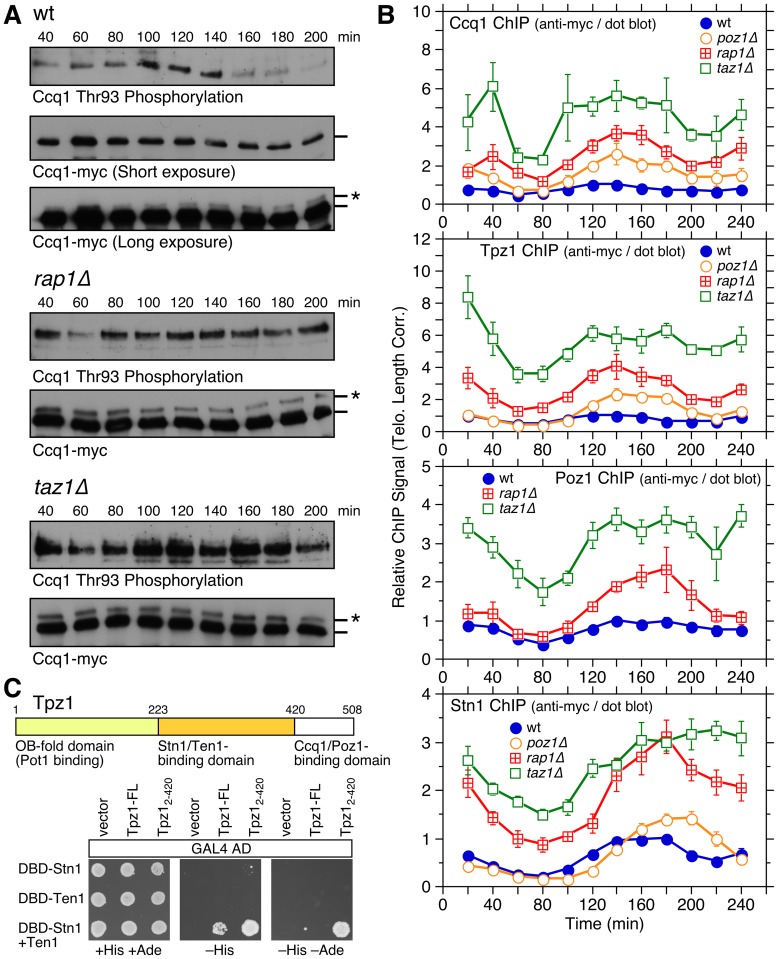
Cell cycle regulation of Ccq1 phosphorylation and cell cycle ChIP assays to monitor telomere association of Stn1 and shelterin subunits Ccq1, Tpz1, and Poz1. (**A**) Western blot analysis of Ccq1 during cell cycle. FLAG-tagged Ccq1 was immunoprecipitated using anti-FLAG antibody, and probed with phospho-(Ser/Thr) ATM/ATR substrate antibody to monitor Ccq1 Thr93 phosphorylation, or probed with anti-FLAG antibody to monitor IP efficiency and existence of slow mobility (marked with *) band corresponding to hyper-phosphorylated Ccq1 [10]. (**B**) Telomere length adjusted ChIP data for Ccq1, Tpz1, and Stn1 in wt, *poz1Δ*, *rap1Δ*, and *taz1Δ* cells, or for Poz1 in wt, *rap1Δ*, and *taz1Δ* cells. Error bars correspond to SEM. Raw ChIP data and % septated cells to monitor cell cycle progression are shown in Figure S12. Anti-myc western blot analyses indicated comparable expression levels in different genetic backgrounds for all proteins (Figure S9E). Comparison of peak normalized ChIP data among shelterin subunits and Stn1 are shown in Figures S13, S14, S15. (**C**) Yeast 3-hybrid assay to monitor Tpz1 to Stn1-Ten1 interaction. Co-expression of DBD-Stn1 and Ten1 was necessary to see interaction between Tpz1 and Stn1-Ten1. Additional truncation analysis of Tpz1 (Figure S16) established amino acids 224–420 of Tpz1 as the minimum binding-domain to the Stn1-Ten1 complex, while the strongest interaction between Tpz1 and Stn1-Ten1 was observed for C-terminally truncated Tpz1 encoding amino acids 2–420.

While massively increased in *rap1Δ* and *taz1Δ* over wt cells, the overall phosphorylation status of Ccq1, monitored by the presence of a slow mobility band of Ccq1 on SDS-PAGE (marked with *), was constant and did not show any cell cycle regulation in all genetic backgrounds tested (Figure 4A). In contrast, Thr93-dependent phosphorylation of Ccq1, detected by phospho-(Ser/Thr) ATM/ATR substrate antibody [10] (see comment in Materials and Methods), showed cell cycle-regulated changes. In wt cells, Thr93 phosphorylation peaked during late S-phase (100–140 min), but was quickly reduced at later time points and nearly abolished at 200 min before cells entered their next S-phase (Figure 4A). Thus, Thr93 phosphorylation was reduced with similar timing as Trt1^TERT^ (Figure 2A–B) and Rad26^ATRIP^ (Figure S11A) binding at 160–200 min. In *rap1Δ* and *taz1Δ* cells, Thr93 phosphorylation was increased throughout the entire cell cycle with slight reductions at 60 and 180–200 min (Figure 4A), but did not entirely match the temporal recruitment pattern of Trt1^TERT^ to telomeres, which showed a dramatic increase in binding in late S-phase. Thus, we concluded that there must be other cell cycle-regulated changes besides Ccq1 Thr93 phosphorylation that regulate Trt1^TERT^ recruitment to telomeres.

### Cell cycle-regulated telomere association of shelterin and Stn1 in wt, *poz1Δ*, *rap1Δ*, and *taz1Δ* cells

Previous ChIP analysis had revealed that the shelterin ssDNA-binding subunit Pot1 along with the CST-complex subunit Stn1 show significant late S-phase specific increases in telomere association that matched to the timing of Polα and Trt1^TERT^ recruitment [25]. We reasoned that cell cycle-regulated changes in shelterin and CST telomere association could dictate Trt1^TERT^ binding, and thus decided to monitor how loss of Poz1, Rap1 and Taz1 affect cell cycle-regulated association of shelterin and CST. We limited our analysis to three subunits of shelterin (Ccq1, Tpz1 and Poz1) and Stn1, and decided to exclude Pot1, since we found that addition of an epitope tag to Pot1 significantly altered telomere length of *poz1Δ*, *rap1Δ* and *taz1Δ* cells.

Consistent with asynchronous ChIP data (Figure S7B), Ccq1, Tpz1, Poz1 and Stn1 all showed gradual increases in overall binding to telomeres in the order of wt, *poz1Δ*, *rap1Δ* and *taz1Δ* when corrected for changes in telomere length (Figure 4B). Ccq1 and Tpz1 showed nearly identical temporal recruitment patterns in wt, *poz1Δ*, *rap1Δ*, and *taz1Δ* cells (Figure S13), while Poz1 recruitment was delayed compared to Ccq1 and Tpz1, and more closely resembled the pattern found for Stn1 (Figures S14 and S15).

We were initially surprised by the similarity of the temporal recruitment patterns for Poz1 and Stn1, as we previously failed to detect interaction between shelterin and Stn1-Ten1 by co-immunoprecipitation [25]. On the other hand, studies in mammalian cells have detected TPP1-CST interaction [23], [35], and we also found by 3-hybrid assay that Tpz1 can interact with Stn1-Ten1 (Figures 4C and S16). Intriguingly, the Tpz1 interaction with Stn1-Ten1 became stronger when the Ccq1/Poz1 interaction domain of Tpz1 (amino acids 421–508) was deleted, suggesting that this domain might negatively regulate the interaction between Tpz1 and Stn1-Ten1. Thus, it is possible that Tpz1-Poz1 interaction might facilitate the timely recruitment of Stn1-Ten1 by reducing the ability of the Tpz1 C-terminal domain to negatively regulate interaction between Tpz1 and Stn1-Ten1.

Comparison with DNA polymerases revealed that Ccq1 and Tpz1 show increases in telomere association along with Polε (80–120 min) and reduction in binding along with Polα (140–220 min) in wt cells (Figure S17). The onsets of increased binding in Ccq1 and Tpz1 remained similar (∼80 min) in the deletion mutants. However, Ccq1 and Tpz1 binding peaked at 140 min, between the peaks for Polε and Polα in *poz1Δ* and *rap1Δ* cells, while they sustained increased binding longer (120–180 min) in *taz1Δ* (Figures 4B and S17). Thus, analogous to Rad26^ATRIP^ (Figure 3), increased binding of Ccq1 and Tpz1 during S-phase in *poz1Δ*, *rap1Δ* and *taz1Δ* cells may be dictated by increased ssDNA caused by deregulated replication of telomeres.

**Figure 5:**
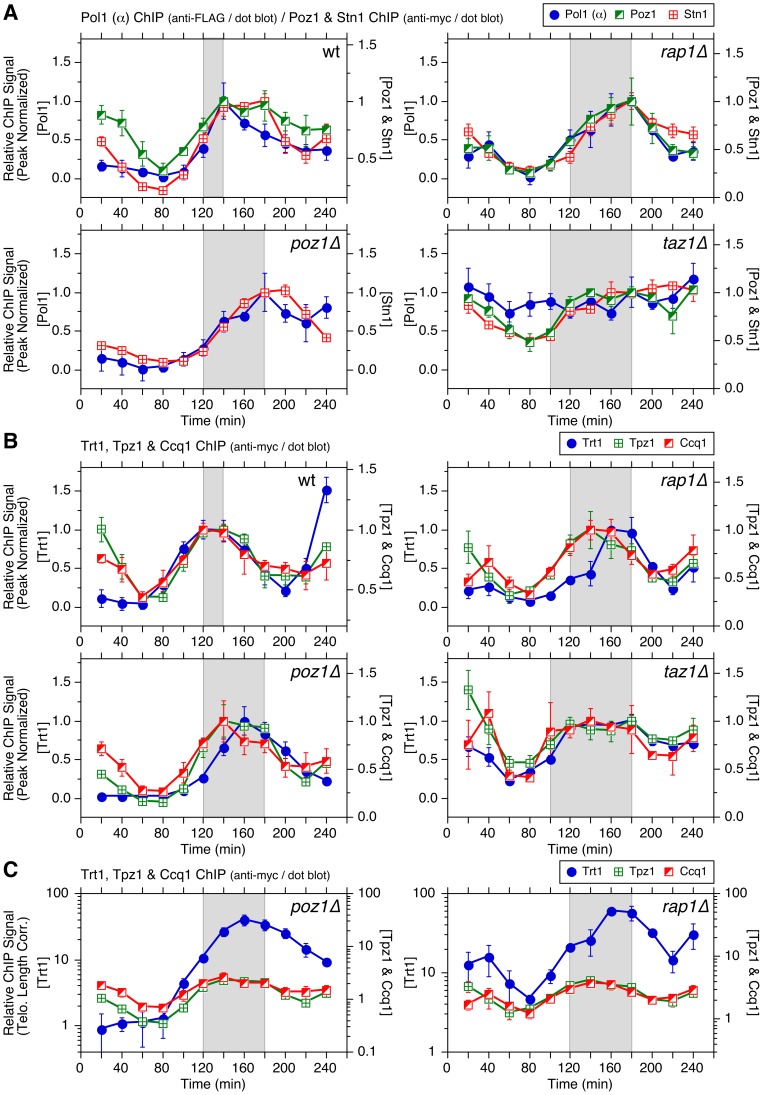
Comparison of cell cycle ChIP data for Trt1^TERT^, DNA polymerases, Poz1/Stn1, and Ccq1/Tpz1. (**A**) Comparison of peak normalized ChIP data for Pol1 (α), Poz1, and Stn1. Comparison for Poz1, Stn1 and Trt1^TERT^ are shown in Figure S18. (**B**) Comparison of peak normalized ChIP data for Trt1^TERT^, Ccq1, and Tpz1. Comparison for Ccq1, Tpz1, and DNA polymerases are shown in Figure S17. (**C**) Comparison of telomere length adjusted ChIP data for Trt1^TERT^, Ccq1, and Tpz1 in *poz1Δ* or *rap1Δ* cells, plotted in log scale. For explanation of shaded areas in graphs, see Figure 2 legend. Error bars correspond to SEM.

In contrast, the temporal binding patterns for Stn1 and Poz1 matched closely with the binding pattern for Polα (Figure 5A) in all genetic backgrounds tested, except for *taz1Δ*. This is consistent with the notion that Poz1 and Stn1 may closely collaborate in promoting the timely recruitment of Polα to telomeres. We also found that Stn1 in wt, *poz1Δ* and *rap1Δ* cells shows more persistent binding at later time points than Polα (Figure 5A), suggesting that Stn1 can sustain increased telomere binding even after Polα dissociates from telomeres. Consistently, we have previously observed increased binding of Stn1 to telomeres in S-phase, even when Polα recruitment was inhibited by HU treatment [25].

Ccq1, Tpz1 and Trt1^TERT^ showed nearly identical overall temporal binding patterns in wt and *taz1Δ* cells, consistent with the notion that cell cycle-regulated binding of Tpz1 and Ccq1 plays a major role in controlling Trt1^TERT^ association with telomeres (Figure 5B). In contrast, Trt1^TERT^ reached its maximal binding later than Ccq1 and Tpz1 in *poz1Δ* and *rap1Δ* cells (Figure 5B). However, this delay is a reflection of the dramatic increase in Trt1^TERT^ binding at 160–200 min in *poz1Δ* and *rap1Δ* cells (Figure 2A–B), a time period in which Ccq1 Thr93 phosphorylation is rapidly reduced in wt cells but remained constitutively high in *rap1Δ* or *taz1Δ* cells (Figure 4A). Indeed, while huge increases in Trt1^TERT^ binding over Tpz1 or Ccq1 had made it difficult to compare cell cycle-regulated patterns in linear scale plots, plotting data on log scale made it more clear that the initial increase in binding of Trt1^TERT^, Ccq1 and Tpz1 occurred with similar timing even in *poz1Δ* and *rap1Δ* cells (Figure 5C).

Poz1 and Stn1 binding to telomeres was delayed compared to Trt1^TERT^ in wt cells, but all three proteins showed very similar overall temporal binding patterns in deletion mutant cells except for more persistent Stn1 binding at later time points (Figure S18A–B). However, since Trt1^TERT^ binding in *poz1Δ*, *rap1Δ* and *taz1Δ* cells increased even in early S-phase, the initial increase in Trt1^TERT^ binding still preceded binding increases of Poz1 and Stn1 in deletion mutant backgrounds (Figure S18C). Taken together, our findings are consistent with the notion that the initial increase in binding of Tpz1 and Ccq1 to telomeres in S-phase contributes to Trt1^TERT^ recruitment, and that a subsequent increase in binding of Poz1 and Stn1 contributes to the timely recruitment of Polα, which limits ssDNA and Rad3^ATR^-Rad26^ATRIP^ accumulation, Ccq1 Thr93 phosphorylation, and telomerase binding at telomeres.

### Contribution of Trt1^TERT^ to regulation of differential temporal binding of DNA Polε and Polα to telomeres

**Figure 6:**
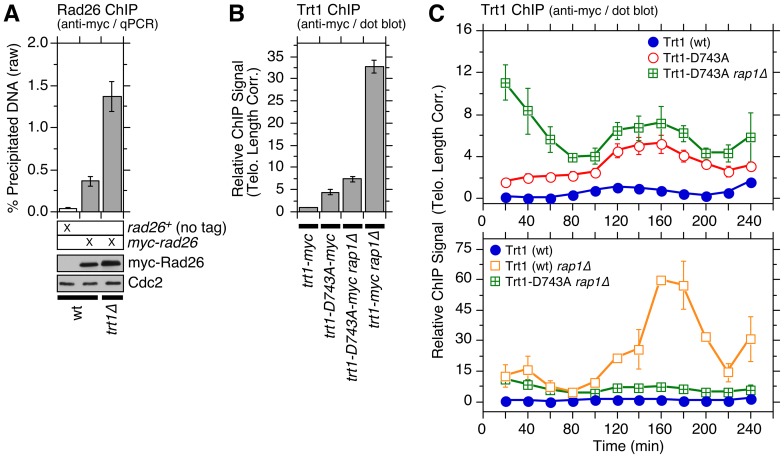
Characterization of telomere association for catalytically dead Trt1^TERT^. (**A**) Comparison of telomere association of Rad26^ATRIP^ in wt and *trt1Δ* cells, monitored by ChIP assay. Rad26^ATRIP^ showed a statistically significant increase in telomere association for *trt1Δ* vs. *trt1^+^* cells (p = 4.6×10^−4^). Anti-myc western blot found comparable Rad26 expression in wt and *trt1Δ* cells. (**B**) Telomere association of wt or catalytically dead (D743A) Trt1^TERT^ in *rap1^+^* or *rap1Δ* backgrounds, monitored by ChIP assay (corrected for telomere length). Trt1-D743A showed a statistically significant increase in telomere association compared to wt Trt1^TERT^ (p = 3.2×10^−5^). Raw ChIP data and expression level of Trt1^TERT^, monitored by anti-myc western blot analysis, are shown in Figure S19B. Data for Trt1-D743A ChIP samples, analyzed by qPCR, are also shown in Figure S19B. Telomere lengths of strains carrying *trt1Δ* or *trt1-D743A* were also monitored by Southern blot analysis (Figure S19A). (**C**) Telomere length corrected cell cycle ChIP assays to monitor association of Trt1^TERT^ with telomeres. Raw and peak normalized ChIP data and % septated cells to monitor cell cycle progression are shown in Figure S19C–E. Error bars correspond to SEM.

Ccq1 Thr93 phosphorylation is also increased in cells carrying short telomeres [10], [13]. As short telomeres would have less binding sites for Taz1 [36], [37], they may become less effective in excluding the Rad3^ATR^-Rad26^ATRIP^ complex from telomeres. Consistently, we found that Rad26^ATRIP^ binding is indeed significantly increased in *trt1Δ* cells (Figure 6A).

While the notion that telomerase is preferentially recruited to short telomeres, due to reduced binding of Taz1 and increased Ccq1 Thr93 phosphorylation, is an attractive model to explain telomere length homeostasis in fission yeast, there has been a lack of any direct evidence that Trt1^TERT^ binding is indeed increased at short telomeres [10]. The problem was difficult to address since mutations previously used to induce telomere shortening (*trt1Δ* or *ccq1-T93A*) eliminated telomerase or its recruitment [10]. We overcame this limitation by monitoring telomere binding of catalytically inactive Trt1^TERT^ (*trt1-D743A*), which causes telomere shortening [38]. Consistent with the prediction, we found that Trt1-D743A binds stronger than wt Trt1^TERT^ to telomeres in asynchronous cell cultures (Figure 6B and S19B), and binds constitutively throughout the cell cycle with increase in binding during S/G_2_-phase (Figure 6C).

Deletion of Rap1 further increased Trt1-D743A binding (Figure 6B–C), especially at the early time points after cell cycle re-entry (20–60 min), but did not greatly affect the temporal recruitment pattern of Trt1-D743A for the remainder of the cell cycle. We are not entirely sure why *trt1-D743A rap1Δ* shows increased Trt1^TERT^ binding at early time points, but binding levels were comparable to wt Trt1^TERT^ in *rap1Δ* cells at 20–80 min (Figure 6C). In contrast, far more wt Trt1^TERT^ was recruited to telomeres than Trt1-D743A for the remainder of the cell cycle in *rap1Δ* cells, suggesting that the massive increase in telomere association of Trt1^TERT^ in *rap1Δ* cells during late S/G_2_-phase is largely dependent on telomerase activity (Figure 6C).

**Figure 7:**
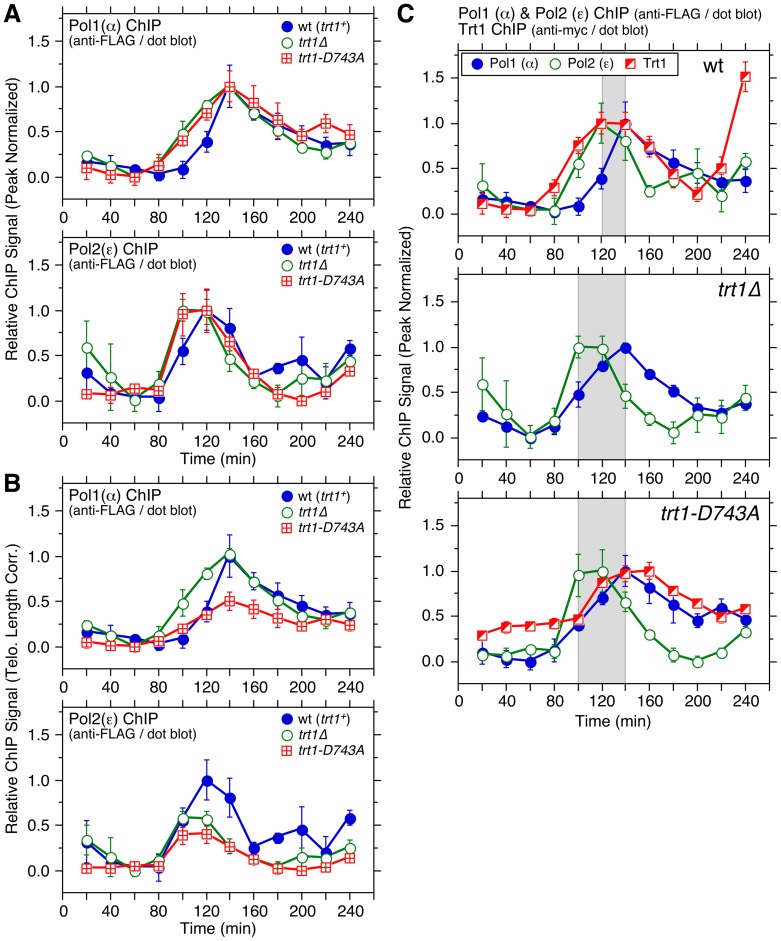
Cell cycle ChIP assays to monitor association of DNA polymerases with telomeres in *trt1Δ* and *trt1-D743A* cells. (**A, B**) Peak normalized (A) or telomere length corrected (B) ChIP data for DNA polymerases. Raw ChIP data and % septated cells to monitor cell cycle progression are shown in Figure S20A–D. For peak normalized Pol1 (α), Student's t-test found p = 0.06 at 100 min (94% confidence level) and p = 0.03 at 120 min (97% confidence level) for wt vs. *trt1Δ* cells, and p = 0.02 at 100 min (98% confidence level) and p = 0.05 at 120 min (95% confidence level) for wt vs. *trt1-D743A* cells. For peak normalized Pol2 (ε), Student's t-test found p = 0.07 at 100 min (93% confidence level) for wt vs. *trt1Δ* cells, and p = 0.21 at 100 min (79% confidence level) for wt vs. *trt1-D743A* cells. For telomere length corrected Pol1 (α), statistically significant differences were found at 120 (p = 4.1×10^−4^), 140 (p = 5.3×10^−3^) and 160 min (4.5×10^−2^) for *trt1Δ* vs. *trt1-D743A* cells. Anti-FLAG western blot analysis indicated comparable expression levels in different genetic backgrounds (Figure S20F). (**C**) Comparison of peak normalized ChIP data for Trt1^TERT^ and DNA polymerases in *wt*, *trt1Δ*, and *trt1-D743A* cells. (Data for wt is identical to Figure 2D, but shown again as a reference.) Statistically significant differences (p<0.04) in telomere binding between Pol1 (α) and Pol2 (ε) were found at 100 and 140–180 min for *trt1Δ* cells, and at 100, 200 and 220 min for *trt1-D743A* cells. Error bars correspond to SEM.

We next investigated how DNA polymerases were affected in *trt1Δ* or *trt1-D743A* cells. Interestingly, Polε binding to telomeres peaked slightly earlier, and the initial increase in Polα binding also occurred slightly earlier in *trt1Δ* and *trt1-D743A* (∼100 min) than wt cells (∼120 min) (Figure 7A). In contrast, we did not see any major change in Polα recruitment timing at *ars2004* in *trt1Δ* cells (Figure S20E). Since *taz1Δ* cells show earlier recruitment of Polε and telomere replication [33] (Figures S4 and S5), it is tempting to speculate that reduced binding of Taz1 at short telomeres might be responsible for earlier Polε recruitment (consistent with earlier telomere replication) for cells carrying short telomeres.

While there was no obvious difference between *trt1Δ* and *trt1-D743A* cells in overall temporal recruitment patterns of Polε and Polα (Figure 7A), when normalized for telomere length, *trt1-D743A* cells had significantly reduced binding of Polα compared to *trt1Δ* cells, suggesting that the presence of catalytically inactive Trt1^TERT^ may interfere with efficient recruitment of Polα (Figure 7B). Our data also indicated that Polε still arrives at telomeres significantly earlier than Polα in *trt1Δ* or *trt1-D743A* cells (Figure 7C), suggesting that telomerase-dependent telomere extension cannot solely be responsible for the differential arrival of Polε and Polα at telomeres.

**Figure 8:**
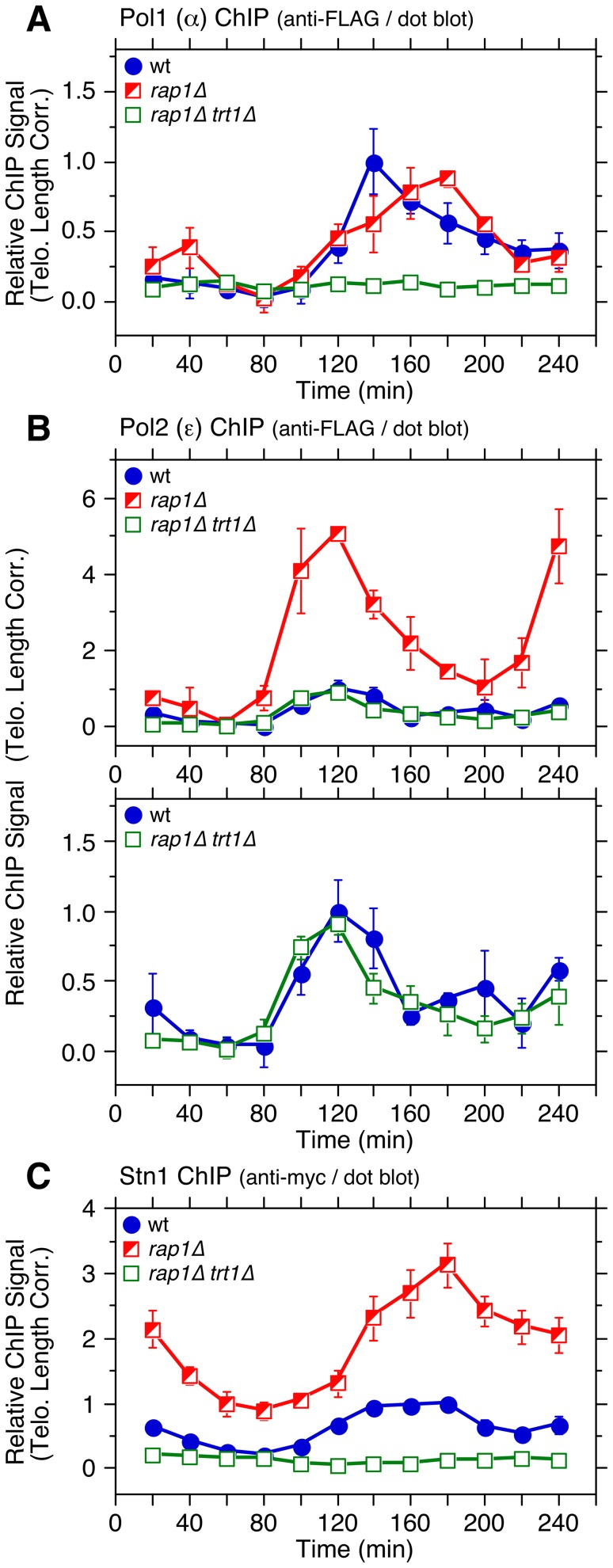
Cell cycle ChIP assays to monitor association of DNA polymerases and Stn1 with telomeres in *rap1Δ trt1Δ* cells. (**A–C**) Telomere length adjusted ChIP data for Pol1 (α) (A), Pol2 (ε) (B), and Stn1 (C). Error bars correspond to SEM. Raw ChIP data and % septated cells to monitor cell cycle progression are shown in Figure S21A–F. Anti-myc and anti-FLAG western blot analysis indicated comparable expression levels in different genetic backgrounds (Figure S21G).

By examining the temporal telomere association patterns of DNA polymerases in *rap1Δ trt1Δ* cells, we attempted to investigate if the delay of Polα arrival at telomeres in *rap1Δ* cells (Figure 2C–D) is dependent on telomerase. To our surprise, *rap1Δ trt1Δ* cells showed very little cell cycle-regulated Polα recruitment to telomeres (Figure 8A), suggesting that Trt1^TERT^ and Rap1 might play redundant roles in coordinating the lagging strand DNA synthesis at telomeres. However, since cells carrying Pol1-FLAG progressed substantially faster through the cell cycle in *trt1Δ rap1Δ* than wt cells (Figure S21D), epitope-tagging of Polα may have introduced unintended changes in telomere regulation that caused synergistic genetic interactions specifically in *rap1Δ trt1Δ* cells. In contrast, we did not see much change in the temporal association pattern of Polε or cell cycle progression between wt and *rap1Δ trt1Δ* for cells carrying Pol2-FLAG (Figures 8B and S21E).

Because studies in other organisms have implicated a connection between Polα and CST in telomere regulation [17]–[19] and our cell cycle ChIP data revealed very similar timings of telomere association for Polα and Stn1 (Figure 5A), we next examined the cell cycle-regulated association of Stn1 in *rap1Δ trt1Δ* cells. Much like Polα, S phase-induced increase in telomere binding of Stn1 was abolished in *rap1Δ trt1Δ* cells (Figure 8C). However, we also noticed that Stn1-myc cells progressed through cell cycle slower in *rap1Δ trt1Δ* (Figure S21F). Thus, epitope-tagging of Stn1 may have elicited unexplained additional telomere defects in *rap1Δ trt1Δ* cells. In any case, it was striking to find loss of cell cycle-regulated binding for both Polα and Stn1 without affecting Polε association in *rap1Δ trt1Δ* cells, and it might indicate that Rap1 and Trt1 play unexpected redundant roles in maintaining proper cell cycle-regulated localization of both Polα and Stn1-Ten1 to telomeres. It is worth noting that a recent study has found that inhibition of telomerase leads to reduced recruitment of Stn1 to telomeres in late S/G_2_-phase in mammalian cells, suggesting that mammalian telomerase also contributes to efficient recruitment of the CST complex to telomeres [23].

## Discussion

A static “shelterin” model [39] has provided a useful framework to understand how various telomere bound factors might be organized together to regulate telomerase action and telomere protection. However, since telomere maintenance regulation is coupled to cell cycle-regulated changes in telomere composition, especially in response to replication of telomeric DNA [1], [2], a new model of telomere regulation that takes cell cycle-regulated changes at telomeres into account must be developed.

In fact, since our current and previous cell cycle ChIP analyses [25] have shown that individual subunits of shelterin show distinct cell cycle-regulated dynamic telomere association patterns, it is likely that the commonly drawn “closed” configuration of the shelterin complex [6] (Figure 1A) that fully connects Taz1 to Pot1 via linker proteins Rap1 and Poz1 may never exist, or exist only in a very limited time window during cell cycle. It should also be noted that recent florescence microcopy analysis [40] and our new ChIP data (Figure 1C) indicate that Rap1 can still be localized to telomeres independently of both Poz1 and Taz1, contrary to a commonly held notion that fission yeast Rap1 recruitment is entirely dependent on Taz1 [6], [8].

**Figure 9:**
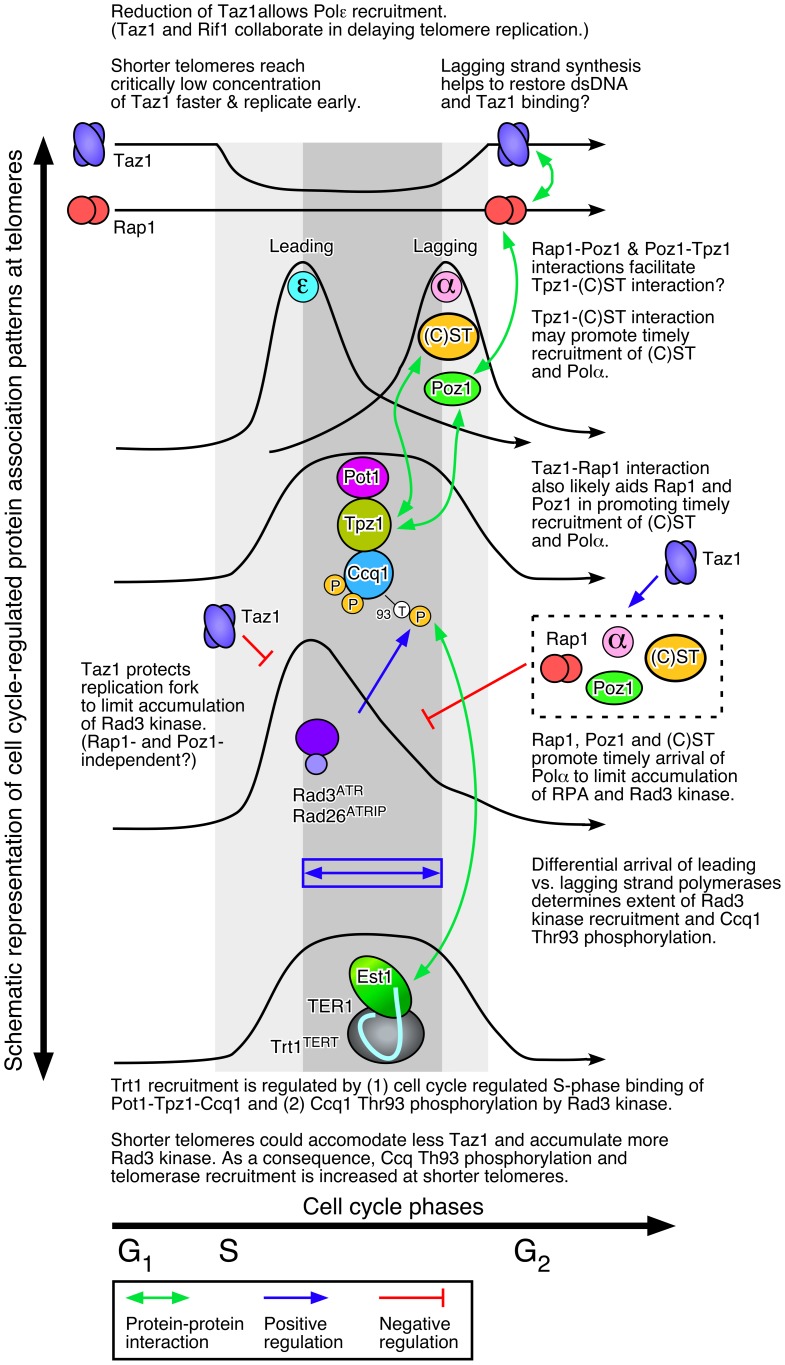
A working model of fission yeast telomere length control.

As a first step toward developing a more dynamic telomere regulation model, we determined detailed cell cycle-regulated telomere association patterns for various factors implicated in telomere regulation in fission yeast. Based on results from current and previous studies, we will propose and discuss a new and more dynamic model of telomere length regulation in fission yeast (Figure 9), which hopefully will serve as a useful framework to guide future investigations.

### Regulation of replicative DNA polymerases at telomeres by shelterin and Stn1-Ten1

While previous studies have implicated Taz1 and TRF1 in efficient replication of telomeric DNA [26], [27], very little was known how loss of Taz1/TRF1 affects replicative DNA polymerases at telomeres. We found that loss of telomerase inhibitors (Poz1, Rap1 and Taz1) differentially affect leading (Polε) and lagging (Polα) strand DNA polymerases (Figure 2C). For *poz1Δ* and *rap1Δ* cells, the peak of Polα binding to telomeres was significantly delayed without affecting Polε, suggesting that Poz1 and Rap1 primarily affect the timely recruitment of the lagging strand DNA polymerase. Consistent with previous studies that observed more severe defects in telomere replication in *taz1Δ* than *rap1Δ* cells [28], [34], [41], Polα binding to telomeres was severely deregulated in *taz1Δ* cells. In addition, loss of Taz1 (but not Rap1 or Poz1) caused earlier recruitment of Polε to telomeres, consistent with recent findings that Taz1 and Taz1-interacting protein Rif1 enforce late S-phase replication of telomeres in fission yeast [33], [42]. Intriguingly, telomerase deficient cells (*trt1Δ* or *trt1-D743A*), which carry shorter telomeres and thus can accommodate less Taz1, also showed slightly earlier recruitment of Polε to telomeres than wt cells, consistent with earlier replication of telomeres (Figure 7).

Taken together, we thus propose that (1) Taz1, likely in collaboration with Rif1 but independently of Poz1 and Rap1, enforces late S-phase replication of telomeres, and as a consequence, (2) shorter telomeres in fission yeast are replicated earlier (Figure 9). Previously, we have found that Taz1 binding is reduced by ∼2-fold during S-phase [25]. Therefore, we speculate that shorter telomeres may be able to reduce Taz1 (and Rif1) density faster to the level compatible with replication, and as a consequence, replicate earlier in S-phase than longer telomeres.

Interestingly, budding yeast cells also replicate shorter telomeres earlier [43], suggesting that early replication of short telomeres may be evolutionarily conserved. Moreover, even though replication timing of mammalian cells appears to be not strictly dependent on telomere length [44], TRF1 binding to telomeres is also reduced during S-phase [45] and Rif1, like fission yeast Rif1, contributes to genome-wide regulation of replication timing [46], [47]. It is thus possible that TRF1 might also collaborate with Rif1 in regulating replication timing at telomeres in mammalian cells.

Epistasis analysis of telomere length by Southern blot indicated that Taz1 carries out both Poz1/Rap1-independent and -dependent roles in regulation of telomere length maintenance (Figure 1B). Accordingly, we further suggest that Taz1, in collaboration with Rap1 and Poz1, also contributes to replication fork integrity at telomeres by promoting timely and cell cycle-regulated recruitment of the lagging strand DNA polymerases (Polα and Polδ) (Figure 9). Completion of the C-strand fill-in synthesis by lagging strand DNA polymerases would then restore dsDNA and Taz1 binding in late S/G_2_ phase, prior to the initiation of mitosis.

Although Rap1 binding to telomeres is not entirely dependent on Taz1, loss of Taz1 significantly reduces Rap1 binding at telomeres (Figure 1C), and disruption of the Taz1-Rap1 interaction causes massive elongation of telomeres, much like *rap1Δ* [40]. Thus, it is easy to imagine how Taz1, through direct interaction with Rap1, would affect Rap1-dependent promotion of lagging strand synthesis at telomeres. Previously identified Rap1-Poz1 and Poz1-Tpz1 interactions [6] would also likely be important in regulating the timely recruitment of lagging strand DNA polymerases (Figure 1A and Figure 9).

In addition, a newly identified interaction between Tpz1 and Stn1-Ten1 (Figure 4C) could play a critical role in allowing Rap1 and Poz1 to enforce the timely recruitment of Polα to telomeres, since our ChIP assays also implicated a close functional relationship among Poz1, Stn1 and Polα in telomere regulation (Figures 5A and 8). Our current findings are likely relevant to understand mammalian telomere regulation, since previous studies have found that mammalian TPP1 also interacts with CST [23], [35], and CST collaborates with Polα in regulating C-strand fill-in synthesis at telomeres [20]–[22].

### Regulation of Rad3^ATR^ kinase recruitment, Ccq1 Thr93 phosphorylation and telomerase recruitment by shelterin and CST

Since Rad3^ATR^/Tel1^ATM^-dependent phosphorylation of Ccq1 at Thr93 promotes Ccq1-Est1 interaction and telomerase recruitment, the mechanism that modulates Thr93 phosphorylation is critical for proper maintenance of telomeres in fission yeast [10], [13]. Poz1, Rap1 and Taz1 negatively regulate Ccq1 Thr93 phosphorylation and telomerase recruitment [10], but the underlying mechanism by which these factors limit Thr93 phosphorylation remained unclear.

Our ChIP data indicated that loss of Poz1, Rap1 and Taz1 causes large increases in telomere association for RPA and Rad3^ATR^-Rad26^ATRIP^, but not Tel1^ATM^ kinase (Figures 3, S7A and S10). Closely matching the extent of increase in Ccq1 Thr93 phosphorylation and Trt1^TERT^ binding [12], RPA and Rad3^ATR^-Rad26^ATRIP^ showed progressive increase in telomere binding in the order of *poz1Δ*, *rap1Δ* and *taz1Δ* cells, especially during S-phase. Therefore, we suggest that Poz1, Rap1 and Taz1 negatively regulate Rad3^ATR^-Rad26^ATRIP^ accumulation and Ccq1 Thr93 phosphorylation by controlling the differential arrival of leading and lagging strand polymerases at telomeres (Figure 9). Based on our cell cycle analysis, we further suggest that S-phase specific Trt1^TERT^ recruitment to telomeres is controlled by both (1) cell cycle-regulated binding of Pot1-Tpz1-Ccq1 and (2) Ccq1 Thr93 phosphorylation. Since Thr93 phosphorylation is quickly lost in wt cells soon after dissociation of Rad26^ATRIP^ from telomeres, it is likely that an unidentified phosphatase is involved in rapidly reducing Thr93 phosphorylation to promote the timely dissociation of Trt1^TERT^ from telomeres. In *poz1Δ*, *rap1Δ* and *taz1Δ* cells, increased accumulation of Rad3^ATR^ kinase results in constitutive Thr93 phosphorylation, hence persistent and high level binding of Trt1^TERT^ in G_2_ phase. We have also shown that catalytically inactive Trt1-D743A shows increased and constitutive binding to telomeres (Figure 6), consistent with the notion that telomerase is preferentially recruited to short telomeres.

The notion that fission yeast utilizes the differential arrival of leading and lagging strand polymerases to control Rad3^ATR^-dependent Ccq1 Thr93 phosphorylation and Trt1^TERT^ recruitment can explain why mutations in Polε lead to shorter telomeres while mutations in Polα and Polδ lead to longer telomeres [48]. Since mutations in Polε would likely delay leading but not lagging strand synthesis, cells would accumulate less ssDNA at telomeres, and as a result, recruit less Rad3^ATR^ and Trt1^TERT^. Conversely, mutations in Polα and Polδ would lead to increased ssDNA, and more robust recruitment of Rad3^ATR^ and telomerase. Effects on differential strand synthesis at telomeres could also explain why *rif1Δ rap1Δ* cells have longer telomeres than *rap1Δ* cells [8], since the loss of Rif1 is expected to advance the arrival of Polε [42], further expanding the differential strand synthesis over *rap1Δ* cells. Differences in Polα binding (Figure 2C) could also explain why *rap1Δ* cells retain S phase-specific G-tail elongation while *taz1Δ* cells show elongated G-tails throughout the cell cycle [34].

Even though budding yeast cells have significantly diverged in telomere protein composition from fission yeast or mammalian cells [4], mutations in Polε also cause telomere shortening while mutations in Polα cause telomere lengthening in budding yeast [49], [50]. Thus, differential regulation of leading and lagging strand synthesis could have evolutionarily conserved roles in telomerase regulation. Studies in mammalian cells have also found that lagging strand synthesis is significantly delayed [51] and regulated by CST [20], [21]. Thus, we believe that our current findings are also relevant in understanding how shelterin and CST regulate telomere maintenance in mammalian cells.

## Materials and Methods

### Yeast strains, plasmids and primers used in this study

Fission yeast strains used in this study were constructed by standard techniques [52], and they are listed in Table S2. For *taz1Δ::ura4^+^*, *taz1Δ::LEU2*, *rap1Δ::ura4^+^*, *poz1Δ::natMX6* and *trt1Δ::his3^+^*, original deletion strains were described previously [8], [30], [36], [53], [54]. For *rad3-kdΔ::kanMX4, ura4^+^* marker was swapped with kanMX4 by (1) PCR amplifying a *kanMX4* module from a pFA6a-kanMX4 plasmid [55] using DNA primers UraKan-T1 and UraKan-B1 (Table S3), and (2) transforming *rad3-kdΔ::ura4^+^* strain [56], [57] with the PCR product. For *rap1-myc*, *trt1-myc*, *pol1-FLAG*, *pol2-FLAG*, *myc-rad3*, *myc-rad26*, *myc-tel1*, *rad11-FLAG*, *ccq1-myc*, *ccq1-FLAG*, *tpz1-myc*, *poz1-myc* and *stn1-myc*, original tagged strains were described previously [9], [12], [25], [57]–[59]. A modified fission yeast strain with *leu1-32::[hENT1 leu1^+^]* and *his3-D1 his7-366::[hsv-tk his7^+^]* that can be used to efficiently incorporate BrdU has been described previously [60]. Strains that carry *trt1-D743A::LEU2* allele at endogenous locus were previously described [61]. A heterozygous diploid strain carrying unmarked *trt1-D743A* or *trt1^+^* was transformed with a PCR product (amplified from a *trt1-G8-13myc::kanMX6* strain using DNA primers trt1-B29 and trt1-T30) to generate cells carrying *trt1-D743A-myc*.

Yeast two/three hybrid assays were performed by mating *Saccharomyces cerevisiae MAT*a (Y2HGold: *MAT*a *trp1-901 leu2-3,-112 ura3-52 his3-200 LYS2::GAL1(UAS)-GAL1(TATA)-HIS3 GAL2(UAS)-GAL2(TATA)-ADE2 gal4Δ gal80Δ URA3::MEL1(UAS)-MEL1(TATA)-AUR1-C MEL1*) strains harboring GAL4-DBD (DNA-binding domain) plasmids with *MAT*α (Y187: *MAT*α *trp1-901 leu2-3,-112 ura3-52 his3-200 ade2-101 gal4Δ gal80Δ met^-^ URA3::GAL1(UAS)-GAL1(TATA)-LacZ MEL1*) strains harboring GAL4-AD (activation domain) plasmids, as described in the MATCHMAKER system manual (Clontech). Plasmids used in yeast two/three hybrid assays are listed in Table S4.

### Southern blot, western blot, and ChIP analyses

Pulsed-field gel electrophoresis to analyze telomere fusions in G_1_ was performed as previously described [31], [62]. Telomere probe used in Southern blot and dot blot-based ChIP was generated as previously described [54], and rDNA probe used to determine telomere length correction factor for dot blot-based ChIP analysis was generated using PCR with primers listed in Table S3 [63], [64]. Primers used in real-time PCR-based ChIP assays are also listed in Table S3. ChIP samples were analyzed with quantitative real-time PCR or dot blot with telomeric probe as previously described [10], [25]. BrdU incorporation was monitored as previously described [25], [65]. Error bars in all plots represent standard error of the mean (SEM) from multiple independent experiments.

For western blot and ChIP assays, either monoclonal anti-myc (9B11, Cell Signaling) or monoclonal anti-FLAG (M2-F1804, Sigma) antibodies were used. Anti-Cdc2 antibody (y100.4, Abcam) was used in western blot analysis as a loading control. Ccq1 Thr93 phosphorylation was monitored using phospho-(Ser/Thr) ATM/ATR substrate antibody (2851, Cell Signaling) as previously described [10]. While not specifically raised against a Ccq1 Thr93 phosphopeptide, our previous analysis indicated that the phospho-(Ser/Thr) ATM/ATR substrate antibody can specifically detect a Ccq1 Thr93 phosphopeptide, and detect a band corresponding to immunoprecipitated Ccq1 that is eliminated in *ccq1-T93A* mutant in western blot analysis [10]. Thus, although we cannot completely eliminate the possibility that this antibody recognizes phosphorylation on other site(s) that might be affected by *ccq1-T93A* mutation, for sake of simplicity, we denote the signal detected by this antibody as Ccq1 Thr93 phosphorylation in the text.

### Establishment of telomere length correction factors

Correction factors for telomere length were established by measuring the hybridization signal intensity of telomere versus rDNA repeats (telomere/rDNA) for *poz1Δ*, *rap1Δ* and *taz1Δ* cells compared to wt cells (Figure S2A and Table S1), using NaOH denatured genomic DNA samples spotted on Nylon membrane by dot blot apparatus. “Telomere length corrected” ChIP values were then calculated by multiplying the background subtracted % precipitated DNA values (raw % precipitated DNA – no tag control % precipitated DNA) with the correction factors, and normalizing them to wt. For telomere length corrected cell cycle ChIP, values were normalized to the peak binding level of wt cells in late S/G_2_-phase. While it may not be a perfect solution, the use of correction factors provided better estimates of changes in protein binding to chromosome ends for cells carrying significantly longer telomeres than wt cells. For telomere length corrected ChIP data, SEM of telomere length corrected ChIP (SEM_Q_) was calculated as 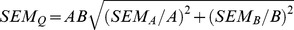 (A = background subtracted ChIP; SEM_A_ = SEM of background subtracted ChIP; B = telomere correction factor; SEM_B_ = SEM of telomere correction factor).

### Statistical analysis

In order to determine the statistical significance of our data, two-tailed Student's t-tests were performed, and p-values≤0.05 (≥95% confidence level) were considered as statistically significant differences.

## Supporting Information

Figure S1 Epistasis analysis of *poz1Δ*, *rap1Δ* and *taz1Δ* cells. (**A**) Telomere length analysis for indicated strains. Genomic DNA was prepared after extensive restreaks on YES plates to ensure telomere length equilibrium. After digestion with ApaI, DNA was fractionated on a 1% agarose gel and processed for Southern blot analysis with a telomere probe. Quantitative analysis of telomere length distribution for this gel is shown in Figure 1B. (**B**) Analysis of cell growth at lower temperatures. Five-fold serial dilution of the indicated strains are plated on YES, and grown at indicated temperatures. (**C**) Chromosome fusion analysis of G_1_ arrested cells. Genomic DNA was prepared in agarose plugs from G_1_ arrested cells, digested with NotI, fractionated on a 1% agarose gel by pulsed-field gel electrophoresis, and processed for Southern blot analysis with probes specific for C, I, L and M NotI chromosomal fragments. A NotI restriction map of *S. pombe* chromosomes is shown below, with telomeric C, I, L, and M fragments marked as black boxes.
(JPG)

Figure S2 Analysis of Trt1^TERT^ recruitment to telomeres by dot blot-based asynchronous ChIP assays with telomeric DNA probe. (**A**) Telomere correction factors for Trt1-myc strains were established by determining telomere/rDNA hybridization signal ratios relative to wt cells. Telomere correction factors for other epitope tagged strains are shown in Supplementary Table S1. (**B**) Raw % precipitated DNA values for dot blot-based Trt1-myc ChIP assays for the indicated genotypes. (**C**) Telomere length corrected ChIP data for Trt1-myc. (See Materials and Methods section for details.) Error bars correspond to SEM.
(JPG)

Figure S3 Raw data of dot blot-based cell cycle ChIP assays for Trt1^TERT^. (**A**, **B**) Cell cycle ChIP assays were performed with *cdc25-22* synchronized cell cultures for wt, *poz1Δ*, *rap1Δ* or *taz1Δ* cells, and % precipitated DNA was determined by hybridization of a telomeric probe to dot blotted input and ChIP samples. (**C**) % septated cells were measured to monitor cell cycle progression of *cdc25-22* synchronized cell cultures for the indicated genotypes. Error bars correspond to SEM.
(JPG)

Figure S4 DNA replication timing monitored by incorporation of BrdU in *cdc25-22* synchronized cells for (**A**) *ars2004* and (**B**) telomeres [25]. BrdU incorporation at telomeres is inhibited by addition of 15 mM HU for wt, *poz1Δ* and *rap1Δ* cells but not for *taz1Δ* cells. BrdU is incorporated into *ars2004* with similar kinetics in the presence or absence of HU for all genetic backgrounds tested. (**C**) Pol1 (α) showed similar timing of recruitment to *ars2004* in all genetic backgrounds tested. Error bars correspond to SEM.
(JPG)

Figure S5 Cell cycle ChIP assays for DNA polymerases. (**A**, **B**) Peak normalized cell cycle ChIP data for Pol1 (α) (A) and Pol2 (ε) (B). For Pol2 (ε), Student's t-test found a statistically significant difference in telomere binding at 80 min (p = 0.03) for wt vs. *taz1Δ* cells. (**C**, **D**) Raw data of dot blot-based cell cycle ChIP assays for Pol1 (α) (C) and Pol2 (ε) (D), performed with *cdc25-22* synchronized cell cultures and telomeric DNA probe. (**E**, **F**) % septated cells were measured to monitor cell cycle progression of *cdc25-22* synchronized cell cultures for Pol1 (α) (E) and Pol2 (ε) (F) ChIP assays. Error bars correspond to SEM. (**G**) Anti-FLAG western blot analysis indicated comparable expression levels in different genetic backgrounds for both Pol1 (α) and Pol2 (ε). Cdc2 western blot served as loading control.
(JPG)

Figure S6 Comparison of cell cycle ChIP data among DNA polymerases and Trt1^TERT^. Comparison of telomere length corrected ChIP data between Pol2 (ε) and Trt1 (**A**) or Pol1 (α) and Trt1 (**B**) in indicated genomic backgrounds. For explanation of shaded areas in graphs, see Figure 2 legend. Error bars correspond to SEM.
(JPG)

Figure S7 Telomere length corrected dot blot-based asynchronous ChIP data for indicated proteins in wt, *poz1Δ*, *rap1Δ* and *taz1Δ* cells. (**A**) Raw ChIP data from Supplementary Figures S8, S9 for Trt1^TERT^, Rad26^ATRIP^, Rad3^ATR^, Rad11^RPA^ and Tpz1 were corrected for telomere length and normalized to wt cells. Compared to wt cells, *poz1Δ*, *rap1Δ*, and *taz1Δ* cells all showed statistically significant increases in telomere association for Trt1^TERT^ (p<1.2×10^−11^), Rad26^ATRIP^ (p<6.4×10^−4^), Rad3^ATR^ (p<0.047 for *poz1Δ* while p<1.8×10^−5^ for *rap1Δ* and *taz1Δ*), Rad11^RPA^ (p<1.6×10^−3^) and Tpz1 (p<3.2×10^−7^). (**B**) Raw ChIP data from Supplementary Figure S9 for Tpz1, Ccq1, Poz1 and Stn1 were corrected for telomere length and normalized to wt cells. Compared to wt cells, *poz1Δ*, *rap1Δ*, and *taz1Δ* cells all showed statistically significant increases in telomere association for Ccq1 (p<1.8×10^−4^), Poz1 (p<1.5×10^−5^) and Stn1 (p<1.1×10^−5^). Error bars correspond to SEM.
(JPG)

Figure S8 Raw % precipitated DNA against input DNA for Rad26^ATRIP^ (**A**), Rad3^ATR^ (**B**) and Rad11^RPA^ (**C**) obtained by dot blot-based asynchronous ChIP assays with telomeric DNA probe. Error bars correspond to SEM. (**D**) Anti-myc (Rad26 and Rad3) and anti-FLAG (Rad11) western blot analysis indicated comparable expression levels in different genetic backgrounds. Cdc2 western blot served as a loading control.
(JPG)

Figure S9 Raw % precipitated DNA against input DNA for Ccq1 (**A**), Tpz1 (**B**), Poz1 (**C**) and Stn1 (**D**) obtained by dot blot-based asynchronous ChIP assays with telomeric DNA probe. Error bars correspond to SEM. (**E**) Anti-myc western blot analyses indicated comparable expression levels for all proteins in different genetic backgrounds. Cdc2 western blot served as a loading control.
(JPG)

Figure S10 Tel1^ATM^ does not show increased binding to telomeres in *poz1Δ*, *rap1Δ* and *taz1Δ* cells. (**A, B**) Raw % precipitated DNA against input DNA for Tel1^ATM^ obtained by dot blot-based asynchronous ChIP assays with telomeric DNA probe. For (A), none of the strains showed statistically significant Tel1^ATM^ binding over no tag controls. For (B), only *rad3-kdΔ* cells [57] showed statistically significant Tel1^ATM^ binding over no tag control (p = 6.0×10^−4^). (**C**) Raw data of dot blot-based cell cycle ChIP assays for Tel1^ATM^ in wt or *rap1Δ* cells, performed with *cdc25-22* synchronized cell cultures and telomeric DNA probe. Among all time points, only wt cells at 80 min showed statistically significant Tel1^ATM^ binding over no tag control (p = 4.0×10^−3^). Error bars correspond to SEM. (**D**) While myc-Tel1 expressed from its endogenous promoter could not be detected in whole cell extracts, comparable amounts of Tel1^ATM^ were immunoprecipitated (IP) with anti-myc antibody in different genetic backgrounds.
(JPG)

Figure S11 Cell cycle ChIP assays for Rad26^ATRIP^ and Rad11^RPA^. (**A**, **B**) Peak normalized cell cycle ChIP data for Rad26 (A) and Rad11 (B). (**C**, **D**) Raw data of dot blot-based cell cycle ChIP assays for Rad26 (C) and Rad11 (D), performed with *cdc25-22* synchronized cell cultures and telomeric DNA probe. (**E**, **F**) % septated cells were measured to monitor cell cycle progression of *cdc25-22* synchronized cell cultures for Rad26 (E) and Rad11 (F) ChIP assays. Error bars correspond to SEM.
(JPG)

Figure S12 Cell cycle ChIP assays for shelterin subunits and Stn1. (**A**–**D**) Raw data of dot blot-based cell cycle ChIP assays for Ccq1 (A), Tpz1 (B), Poz1 (C) and Stn1 (D), performed with *cdc25-22* synchronized cell cultures and telomeric DNA probe. (**E**–**H**) % septated cells were measured to monitor cell cycle progression of *cdc25-22* synchronized cell cultures for Ccq1 (E), Tpz1 (F), Poz1 (G) and Stn1 (H) ChIP assays. Error bars correspond to SEM.
(JPG)

Figure S13 Comparison of peak normalized cell cycle ChIP data between Ccq1 and Tpz1. (**A**) Peak normalized ChIP data for either Ccq1 or Tpz1 in different genetic backgrounds were plotted to compare changes in temporal association with telomeres. (**B**) Comparison of peak normalized ChIP data indicated that temporal changes in telomere association for Ccq1 and Tpz1 are nearly identical in all genetic backgrounds tested. For explanation of shaded areas in graphs, see Figure 2 legend. Error bars correspond to SEM.
(JPG)

Figure S14 Comparison of peak normalized cell cycle ChIP data between Poz1 and Stn1. (**A**) Peak normalized ChIP data for either Poz1 or Stn1 in different genetic backgrounds were plotted to compare changes in temporal association with telomeres. (**B**) Comparison of peak normalized ChIP data indicated that temporal changes in telomere association for Poz1 and Stn1 are nearly identical in wt, *rap1Δ* and *taz1Δ* cells. For explanation of shaded areas in graphs, see Figure 2 legend. Error bars correspond to SEM.
(JPG)

Figure S15 Comparison of cell cycle ChIP data among Ccq1, Tpz1, Poz1 and Stn1. (**A**) Comparison of peak normalized ChIP data for Poz1, Tpz1 and Ccq1 in wt, *rap1Δ* and *taz1Δ* cells. For Tpz1 vs. Poz1, Student's t-test found p = 0.053 at 120 min (94.7% confidence level) for wt cells, and p = 0.058 at 80 min (94.2% confidence level) and p = 0.09 at 100 min (91% confidence level) for *rap1Δ* cells. For Ccq1 vs. Poz1, Student's t-test found p = 0.045 at 100 min (95.5% confidence level) and p = 0.071 at 120 min (92.9% confidence level) for wt cells, and p = 0.082 at 100 min (91.8% confidence level) for *rap1Δ* cells. (**B**) Comparison of peak normalized ChIP data for Stn1, Tpz1 and Ccq1 in wt, *poz1Δ*, *rap1Δ* and *taz1Δ* cells. For Tpz1 vs. Stn1, differences were statistically significant at 60–120 min for wt cells (p<0.03), at 100, 120, 200 and 220 min for *poz1Δ* cells (p<0.04), and at 100, 120, 200 min for *rap1Δ* cells (p<0.01). For Ccq1 vs. Stn1, differences were statistically significant at 100, 120 and 180 min for wt cells (p<0.03), at 80 and 120 min for *poz1Δ* cells (p<0.04), and at 100, 120, 200 min for *rap1Δ* cells (p<0.02). For explanation of shaded areas in graphs, see Figure 2 legend. Error bars correspond to SEM.
(JPG)

Figure S16 Yeast 3-hybrid assay to monitor interaction between Tpz1 and Stn1-Ten1. Various truncation constructs of Tpz1 were tested for interaction with Stn1 and Ten1. Based on cell growth on –His selection plate, a Tpz1 fragment containing amino acids 224–420 was the smallest Tpz1 construct that retained interaction with Stn1 and Ten1. Based on growth on –His –Ade plate, a Tpz1 fragment containing amino acids 2–420 showed strongest interaction with Stn1 and Ten1.
(JPG)

Figure S17 Comparison of cell cycle ChIP data among DNA polymerases, Ccq1 and Tpz1. Comparison of peak normalized ChIP data for Pol1 (α), Pol2 (ε) and Ccq1 (**A**) or Pol1 (α), Pol2 (ε) and Tpz1 (**B**) in wt, *poz1Δ*, *rap1Δ*, and *taz1Δ* cells. For explanation of shaded areas in graphs, see Figure 2 legend. Error bars correspond to SEM.
(JPG)

Figure S18 Comparison of cell cycle ChIP data among Trt1^TERT^, Poz1 and Stn1. Comparison of peak normalized ChIP data between Trt1 and Poz1 (**A**) or Trt1 and Stn1 (**B**) for indicated genomic backgrounds. (**C**) Comparison of peak normalized ChIP data among Trt1, Poz1 and Stn1 in *poz1Δ* or *rap1Δ*, plotted on log scale. For explanation of shaded areas in graphs, see Figure 2 legend. Error bars correspond to SEM.
(JPG)

Figure S19 Cell cycle ChIP assays for catalytically dead Trt1-D743A. (**A**) Telomere length analysis for indicated strains used in ChIP analysis. Genomic DNA was prepared from early generation strains. After digestion with EcoRI, DNA was fractionated on a 1% agarose gel and processed for Southern blot analysis with a telomere probe. (**B**) Raw % precipitated DNA against input DNA for Trt1^TERT^ obtained by real-time quantitative PCR analysis (left) or dot blot-based asynchronous ChIP assays with telomeric DNA probe (right). Trt1-D743A showed a statistically significant increase in telomere association compared to wt Trt1^TERT^ (p = 5.4×10^−5^) for PCR-based ChIP assay, independently confirming our conclusion from telomere-length corrected dot blot-based ChIP assay (Figure 6B). Anti-myc western blot analysis indicated comparable expression levels of Trt1 in different genetic backgrounds. Cdc2 western blot served as a loading control. (**C**) Peak normalized cell cycle ChIP data for wt or catalytically dead Trt1^TERT^ in *rap1^+^* or *rap1Δ* cells. (**D**) Raw data of dot blot-based cell cycle ChIP assays for Trt1^TERT^, performed with *cdc25-22* synchronized cell cultures and telomeric DNA probe. (**E**) % septated cells were measured to monitor cell cycle progression of *cdc25-22* synchronized cell cultures for Trt1^TERT^ ChIP assays. Error bars correspond to SEM.
(JPG)

Figure S20 Cell cycle ChIP assays for DNA polymerases in *trt1* mutants. (**A**, **B**) Raw data of dot blot-based cell cycle ChIP assays for Pol1 (α) (A) and Pol2 (ε) (B), performed with *cdc25-22* synchronized cell cultures and telomeric DNA probe. (**C**, **D**) % septated cells were measured to monitor cell cycle progression of *cdc25-22* synchronized cell cultures for Pol1 (α) (C) and Pol2 (ε) (D) ChIP assays. (**E**) Pol1 (α) showed similar timing of recruitment to *ars2004* in wt and *trt1Δ* cells. Error bars correspond to SEM. (**F**) Anti-FLAG western blot analysis indicated comparable expression levels in different genetic backgrounds for both Pol1 and Pol2. Cdc2 western blot served as a loading control.
(JPG)

Figure S21 Cell cycle ChIP assays for DNA polymerases and Stn1 in *rap1Δ trt1Δ* cells. (**A**–**C**) Raw data of dot blot-based cell cycle ChIP assays for Pol1 (α) (A), Pol2 (ε) (B) and Stn1 (C), performed with *cdc25-22* synchronized cell cultures and telomeric DNA probe. (**D**–**F**) % septated cells were measured to monitor cell cycle progression of *cdc25-22* synchronized cell cultures for Pol1 (D), Pol2 (E) and Stn1 (F) ChIP assays. Error bars correspond to SEM. (**G**) Anti-FLAG (Pol1 and Pol2) and anti-myc (Stn1) western blot analyses indicated comparable expression levels in different genetic backgrounds for both Pol1 (α) and Pol2 (ε). Cdc2 western blot served as a loading control.
(JPG)

Table S1 Telomere length correction factors (telomere/rDNA) for dot blot-based ChIP.
(PDF)

Table S2 Fission yeast strains used in this study.
(PDF)

Table S3 DNA primers used in this study.
(PDF)

Table S4 Plasmids used in this study.
(PDF)

Supporting Information S1 A single PDF file containing all Supporting Information (Figures S1–S21 and Tables S1–S4).
(PDF)

## References

[1] Verdun RE, Karlseder J (2007) Replication and protection of telomeres. Nature 447: 924–931. 17581575 10.1038/nature05976

[2] Gilson E, Geli V (2007) How telomeres are replicated. Nat Rev Mol Cell Biol 8: 825–838. 17885666 10.1038/nrm2259

[3] Blackburn EH, Greider CW, Szostak JW (2006) Telomeres and telomerase: the path from maize, *Tetrahymena* and yeast to human cancer and aging. Nat Med 12: 1133–1138. 17024208 10.1038/nm1006-1133

[4] Palm W, de Lange T (2008) How shelterin protects mammalian telomeres. Annu Rev Genet 42: 301–334. 18680434 10.1146/annurev.genet.41.110306.130350

[5] Armanios M, Blackburn EH (2012) The telomere syndromes. Nat Rev Genet 13: 693–704. 22965356 10.1038/nrg3246 PMC3548426

[6] Miyoshi T, Kanoh J, Saito M, Ishikawa F (2008) Fission yeast Pot1-Tpp1 protects telomeres and regulates telomere length. Science 320: 1341–1344. 18535244 10.1126/science.1154819

[7] Moser BA, Nakamura TM (2009) Protection and replication of telomeres in fission yeast. Biochem Cell Biol 87: 747–758. 19898524 10.1139/o09-037 PMC2854563

[8] Kanoh J, Ishikawa F (2001) spRap1 and spRif1, recruited to telomeres by Taz1, are essential for telomere function in fission yeast. Curr Biol 11: 1624–1630. 11676925 10.1016/s0960-9822(01)00503-6

[9] Tomita K, Cooper JP (2008) Fission yeast Ccq1 is telomerase recruiter and local checkpoint controller. Genes Dev 22: 3461–3474. 19141478 10.1101/gad.498608 PMC2607071

[10] Moser BA, Chang YT, Kosti J, Nakamura TM (2011) Tel1^ATM^ and Rad3^ATR^ kinases promote Ccq1-Est1 interaction to maintain telomeres in fission yeast. Nat Struct Mol Biol 18: 1408–1413. 22101932 10.1038/nsmb.2187 PMC3230746

[11] Naito T, Matsuura A, Ishikawa F (1998) Circular chromosome formation in a fission yeast mutant defective in two ATM homologues. Nat Genet 20: 203–206. 9771717 10.1038/2517

[12] Moser BA, Subramanian L, Khair L, Chang YT, Nakamura TM (2009) Fission yeast Tel1^ATM^ and Rad3^ATR^ promote telomere protection and telomerase recruitment. PLoS Genet 5: e1000622. 19714219 10.1371/journal.pgen.1000622 PMC2726628

[13] Yamazaki H, Tarumoto Y, Ishikawa F (2012) Tel1^ATM^ and Rad3^ATR^ phosphorylate the telomere protein Ccq1 to recruit telomerase and elongate telomeres in fission yeast. Genes Dev 26: 241–246. 22302936 10.1101/gad.177873.111 PMC3278891

[14] Miyake Y, Nakamura M, Nabetani A, Shimamura S, Tamura M, et al (2009) RPA-like mammalian Ctc1-Stn1-Ten1 complex binds to single-stranded DNA and protects telomeres independently of the Pot1 pathway. Mol Cell 36: 193–206. 19854130 10.1016/j.molcel.2009.08.009

[15] Surovtseva YV, Churikov D, Boltz KA, Song X, Lamb JC, et al (2009) Conserved telomere maintenance component 1 interacts with STN1 and maintains chromosome ends in higher eukaryotes. Mol Cell 36: 207–218. 19854131 10.1016/j.molcel.2009.09.017 PMC2768651

[16] Price CM, Boltz KA, Chaiken MF, Stewart JA, Beilstein MA, et al (2010) Evolution of CST function in telomere maintenance. Cell Cycle 9: 3157–3165. 20697207 10.4161/cc.9.16.12547 PMC3041159

[17] Casteel DE, Zhuang S, Zeng Y, Perrino FW, Boss GR, et al (2009) A DNA polymerase-α•primase cofactor with homology to replication protein A-32 regulates DNA replication in mammalian cells. J Biol Chem 284: 5807–5818. 19119139 10.1074/jbc.M807593200 PMC2645831

[18] Puglisi A, Bianchi A, Lemmens L, Damay P, Shore D (2008) Distinct roles for yeast Stn1 in telomere capping and telomerase inhibition. EMBO J 27: 2328–2339. 19172739 10.1038/emboj.2008.158 PMC2529371

[19] Qi H, Zakian VA (2000) The *Saccharomyces* telomere-binding protein Cdc13p interacts with both the catalytic subunit of DNA polymerase α and the telomerase-associated Est1 protein. Genes Dev 14: 1777–1788. 10898792 PMC316788

[20] Wang F, Stewart JA, Kasbek C, Zhao Y, Wright WE, et al (2012) Human CST has independent functions during telomere duplex replication and C-strand fill-in. Cell Rep 2: 1096–1103. 23142664 10.1016/j.celrep.2012.10.007 PMC3513692

[21] Wu P, Takai H, de Lange T (2012) Telomeric 3′ overhangs derive from resection by Exo1 and Apollo and fill-in by POT1b-associated CST. Cell 150: 39–52. 22748632 10.1016/j.cell.2012.05.026 PMC3392515

[22] Nakaoka H, Nishiyama A, Saito M, Ishikawa F (2012) *Xenopus laevis* Ctc1-Stn1-Ten1 (xCST) protein complex is involved in priming DNA synthesis on single-stranded DNA template in Xenopus egg extract. J Biol Chem 287: 619–627. 22086929 10.1074/jbc.M111.263723 PMC3249116

[23] Chen LY, Redon S, Lingner J (2012) The human CST complex is a terminator of telomerase activity. Nature 488: 540–544. 22763445 10.1038/nature11269

[24] Martin V, Du LL, Rozenzhak S, Russell P (2007) Protection of telomeres by a conserved Stn1-Ten1 complex. Proc Natl Acad Sci U S A 104: 14038–14043. 17715303 10.1073/pnas.0705497104 PMC1955774

[25] Moser BA, Subramanian L, Chang YT, Noguchi C, Noguchi E, et al (2009) Differential arrival of leading and lagging strand DNA polymerases at fission yeast telomeres. EMBO J 28: 810–820. 19214192 10.1038/emboj.2009.31 PMC2670859

[26] Miller KM, Rog O, Cooper JP (2006) Semi-conservative DNA replication through telomeres requires Taz1. Nature 440: 824–828. 16598261 10.1038/nature04638

[27] Sfeir A, Kosiyatrakul ST, Hockemeyer D, MacRae SL, Karlseder J, et al (2009) Mammalian telomeres resemble fragile sites and require TRF1 for efficient replication. Cell 138: 90–103. 19596237 10.1016/j.cell.2009.06.021 PMC2723738

[28] Miller KM, Ferreira MG, Cooper JP (2005) Taz1, Rap1 and Rif1 act both interdependently and independently to maintain telomeres. EMBO J 24: 3128–3135. 16096639 10.1038/sj.emboj.7600779 PMC1201358

[29] Chikashige Y, Hiraoka Y (2001) Telomere binding of the Rap1 protein is required for meiosis in fission yeast. Curr Biol 11: 1618–1623. 11676924 10.1016/s0960-9822(01)00457-2

[30] Khair L, Subramanian L, Moser BA, Nakamura TM (2009) Roles of heterochromatin and telomere proteins in regulation of fission yeast telomere recombination and telomerase recruitment. J Biol Chem 285: 5327–5337. 20040595 10.1074/jbc.M109.078840 PMC2820761

[31] Ferreira MG, Cooper JP (2001) The fission yeast Taz1 protein protects chromosomes from Ku-dependent end-to-end fusions. Mol Cell 7: 55–63. 11172711 10.1016/s1097-2765(01)00154-x

[32] Fujita I, Tanaka M, Kanoh J (2012) Identification of the functional domains of the telomere protein Rap1 in *Schizosaccharomyces pombe* . PLoS One 7: e49151. 23133674 10.1371/journal.pone.0049151 PMC3487762

[33] Tazumi A, Fukuura M, Nakato R, Kishimoto A, Takenaka T, et al (2012) Telomere-binding protein Taz1 controls global replication timing through its localization near late replication origins in fission yeast. Genes Dev 26: 2050–2062. 22987637 10.1101/gad.194282.112 PMC3444731

[34] Dehe PM, Rog O, Ferreira MG, Greenwood J, Cooper JP (2012) Taz1 enforces cell-cycle regulation of telomere synthesis. Mol Cell 46: 797–808. 22633956 10.1016/j.molcel.2012.04.022

[35] Wan M, Qin J, Songyang Z, Liu D (2009) OB fold-containing protein 1 (OBFC1), a human homolog of yeast Stn1, associates with TPP1 and is implicated in telomere length regulation. J Biol Chem 284: 26725–26731. 19648609 10.1074/jbc.M109.021105 PMC2785360

[36] Cooper JP, Nimmo ER, Allshire RC, Cech TR (1997) Regulation of telomere length and function by a Myb-domain protein in fission yeast. Nature 385: 744–747. 9034194 10.1038/385744a0

[37] Tomaska L, Willcox S, Slezakova J, Nosek J, Griffith JD (2004) Taz1 binding to a fission yeast model telomere: formation of telomeric loops and higher order structures. J Biol Chem 279: 50764–50772. 15383525 10.1074/jbc.M409790200

[38] Haering CH, Nakamura TM, Baumann P, Cech TR (2000) Analysis of telomerase catalytic subunit mutants *in vivo* and *in vitro* in *Schizosaccharomycespombe* . Proc Natl Acad Sci U S A 97: 6367–6372. 10829083 10.1073/pnas.130187397 PMC18609

[39] de Lange T (2005) Shelterin: the protein complex that shapes and safeguards human telomeres. Genes Dev 19: 2100–2110. 16166375 10.1101/gad.1346005

[40] Chen Y, Rai R, Zhou ZR, Kanoh J, Ribeyre C, et al (2011) A conserved motif within RAP1 has diversified roles in telomere protection and regulation in different organisms. Nat Struct Mol Biol 18: 213–221. 21217703 10.1038/nsmb.1974 PMC3688267

[41] Miller KM, Cooper JP (2003) The telomere protein Taz1 is required to prevent and repair genomic DNA breaks. Mol Cell 11: 303–313. 12620220 10.1016/s1097-2765(03)00041-8

[42] Hayano M, Kanoh Y, Matsumoto S, Renard-Guillet C, Shirahige K, et al (2012) Rif1 is a global regulator of timing of replication origin firing in fission yeast. Genes Dev 26: 137–150. 22279046 10.1101/gad.178491.111 PMC3273838

[43] Bianchi A, Shore D (2007) Early replication of short telomeres in budding yeast. Cell 128: 1051–1062. 17382879 10.1016/j.cell.2007.01.041

[44] Arnoult N, Schluth-Bolard C, Letessier A, Drascovic I, Bouarich-Bourimi R, et al (2010) Replication timing of human telomeres is chromosome arm-specific, influenced by subtelomeric structures and connected to nuclear localization. PLoS Genet 6: e1000920. 20421929 10.1371/journal.pgen.1000920 PMC2858680

[45] Verdun RE, Crabbe L, Haggblom C, Karlseder J (2005) Functional human telomeres are recognized as DNA damage in G2 of the cell cycle. Mol Cell 20: 551–561. 16307919 10.1016/j.molcel.2005.09.024

[46] Yamazaki S, Ishii A, Kanoh Y, Oda M, Nishito Y, et al (2012) Rif1 regulates the replication timing domains on the human genome. EMBO J 31: 3667–3677. 22850674 10.1038/emboj.2012.180 PMC3442267

[47] Cornacchia D, Dileep V, Quivy JP, Foti R, Tili F, et al (2012) Mouse Rif1 is a key regulator of the replication-timing programme in mammalian cells. EMBO J 31: 3678–3690. 22850673 10.1038/emboj.2012.214 PMC3442270

[48] Dahlen M, Sunnerhagen P, Wang TS (2003) Replication proteins influence the maintenance of telomere length and telomerase protein stability. Mol Cell Biol 23: 3031–3042. 12697806 10.1128/MCB.23.9.3031-3042.2003 PMC153188

[49] Adams-Martin A, Dionne I, Wellinger RJ, Holm C (2000) The function of DNA polymerase alpha at telomeric G tails is important for telomere homeostasis. Mol Cell Biol 20: 786–796. 10629035 10.1128/mcb.20.3.786-796.2000 PMC85195

[50] Ohya T, Kawasaki Y, Hiraga S, Kanbara S, Nakajo K, et al (2002) The DNA polymerase domain of polε is required for rapid, efficient, and highly accurate chromosomal DNA replication, telomere length maintenance, and normal cell senescence in Saccharomyces cerevisiae. J Biol Chem 277: 28099–28108. 12015307 10.1074/jbc.M111573200

[51] Zhao Y, Sfeir AJ, Zou Y, Buseman CM, Chow TT, et al (2009) Telomere extension occurs at most chromosome ends and is uncoupled from fill-in in human cancer cells. Cell 138: 463–475. 19665970 10.1016/j.cell.2009.05.026 PMC2726829

[52] Alfa C, Fantes P, Hyams J, McLoed M, Warbrick E (1993) Experiments with Fission Yeast. Cold Spring Harbor, NY: Cold Spring Harbor Laboratory Press.

[53] Nakamura TM, Moser BA, Russell P (2002) Telomere binding of checkpoint sensor and DNA repair proteins contributes to maintenance of functional fission yeast telomeres. Genetics 161: 1437–1452. 12196391 10.1093/genetics/161.4.1437 PMC1462227

[54] Nakamura TM, Morin GB, Chapman KB, Weinrich SL, Andrews WH, et al (1997) Telomerase catalytic subunit homologs from fission yeast and human. Science 277: 955–959. 9252327 10.1126/science.277.5328.955

[55] Wach A, Brachat A, Pohlmann R, Philippsen P (1994) New heterologous modules for classical or PCR-based gene disruptions in *Saccharomyces cerevisiae* . Yeast 10: 1793–1808. 7747518 10.1002/yea.320101310

[56] Bentley NJ, Holtzman DA, Flaggs G, Keegan KS, DeMaggio A, et al (1996) The *Schizosaccharomyces pombe rad3* checkpoint gene. EMBO J 15: 6641–6651. 8978690 PMC452488

[57] Subramanian L, Nakamura TM (2010) A kinase-independent role for the Rad3^ATR^-Rad26^ATRIP^ complex in recruitment of Tel1^ATM^ to telomeres in fission yeast. PLoS Genet 6: e1000839. 20140190 10.1371/journal.pgen.1000839 PMC2816689

[58] Webb CJ, Zakian VA (2008) Identification and characterization of the *Schizosaccharomyces pombe* TER1 telomerase RNA. Nat Struct Mol Biol 15: 34–42. 18157149 10.1038/nsmb1354 PMC2703720

[59] Noguchi E, Noguchi C, McDonald WH, Yates JR (2004) Swi1 and Swi3 are components of a replication fork protection complex in fission yeast. Mol Cell Biol 24: 8342–8355. 15367656 10.1128/MCB.24.19.8342-8355.2004 PMC516732

[60] Hodson JA, Bailis JM, Forsburg SL (2003) Efficient labeling of fission yeast *Schizosaccharomyces pombe* with thymidine and BUdR. Nucleic Acids Res 31: e134. 14576334 10.1093/nar/gng134 PMC275491

[61] Subramanian L, Moser BA, Nakamura TM (2008) Recombination-based telomere maintenance is dependent on Tel1-MRN and Rap1 and inhibited by telomerase, Taz1, and Ku in fission yeast. Mol Cell Biol 28: 1443–1455. 18160711 10.1128/MCB.01614-07 PMC2258766

[62] Nakamura TM, Cooper JP, Cech TR (1998) Two modes of survival of fission yeast without telomerase. Science 282: 493–496. 9774280 10.1126/science.282.5388.493

[63] Kanoh J, Sadaie M, Urano T, Ishikawa F (2005) Telomere binding protein Taz1 establishes Swi6 heterochromatin independently of RNAi at telomeres. Curr Biol 15: 1808–1819. 16243027 10.1016/j.cub.2005.09.041

[64] Hayashi M, Katou Y, Itoh T, Tazumi A, Yamada Y, et al (2007) Genome-wide localization of pre-RC sites and identification of replication origins in fission yeast. EMBO J 26: 1327–1339. 17304213 10.1038/sj.emboj.7601585 PMC1817633

[65] Sivakumar S, Porter-Goff M, Patel PK, Benoit K, Rhind N (2004) *In vivo* labeling of fission yeast DNA with thymidine and thymidine analogs. Methods 33: 213–219. 15157888 10.1016/j.ymeth.2003.11.016 PMC5074384

