# Supplementary figures and images for: Fission Yeast Shelterin Regulates DNA Polymerases and Rad3ATR Kinase to Limit Telomere Extension
Source: PLoS Genet. 2013 Nov 7;9(11):e1003936. doi: 10.1371/journal.pgen.1003936 (PMC3820796; doi:10.1371/journal.pgen.1003936)

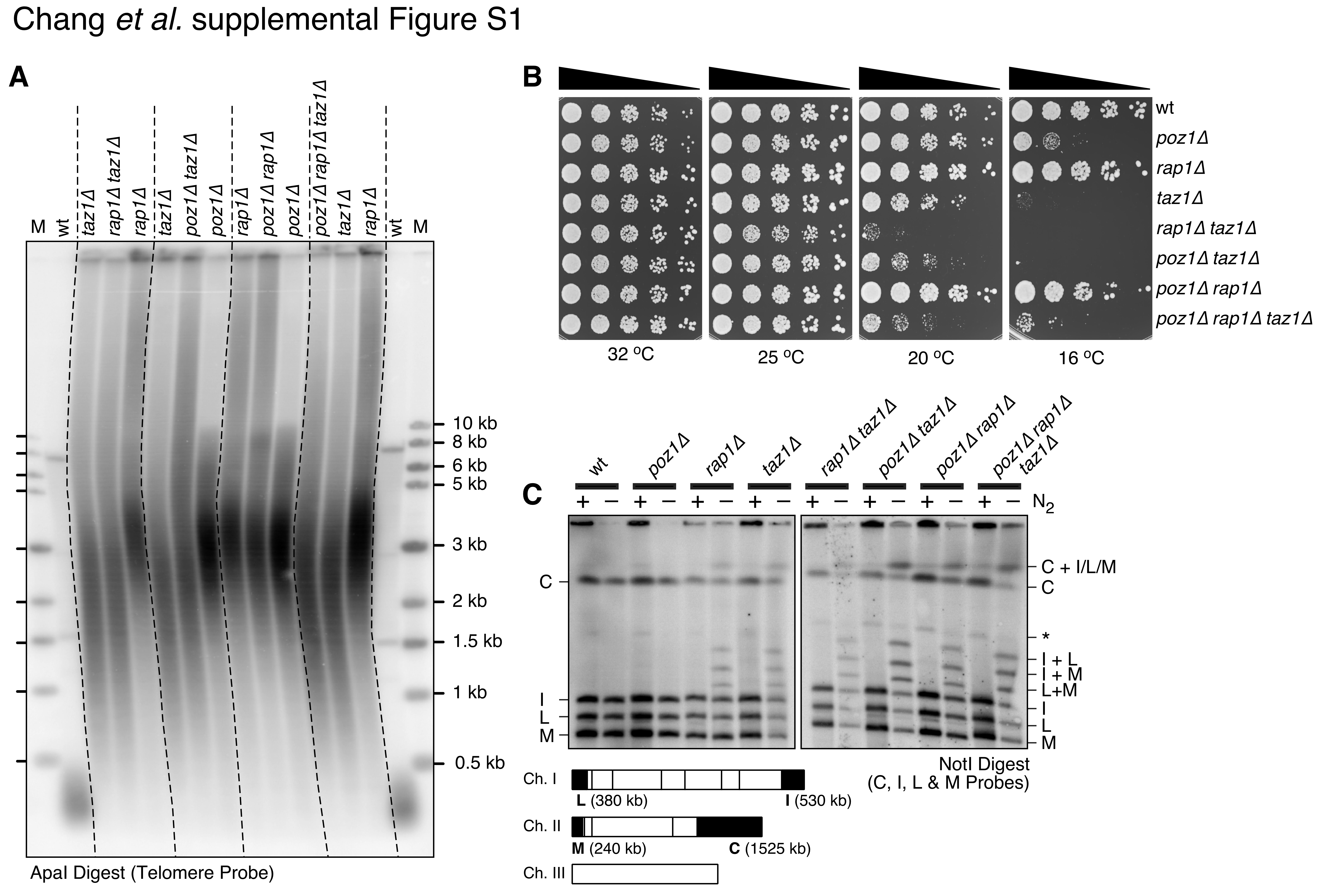

Supplement: Figure S1 — Epistasis analysis of poz1Δ, rap1Δ and taz1Δ cells. (A) Telomere length analysis for indicated strains. Genomic DNA was prepared after extensive restreaks on YES plates to ensure telomere length equilibrium. After digestion with ApaI, DNA was fractionated on a 1% agarose gel and processed for Southern blot analysis with a telomere probe. Quantitative analysis of telomere length distribution for this gel is shown in Figure 1B. (B) Analysis of cell growth at lower temperatures. Five-fold serial dilution of the indicated strains are plated on YES, and grown at indicated temperatures. (C) Chromosome fusion analysis of G1 arrested cells. Genomic DNA was prepared in agarose plugs from G1 arrested cells, digested with NotI, fractionated on a 1% agarose gel by pulsed-field gel electrophoresis, and processed for Southern blot analysis with probes specific for C, I, L and M NotI chromosomal fragments. A NotI restriction map of S. pombe chromosomes is shown below, with telomeric C, I, L, and M fragments marked as black boxes. (JPG) [file pgen.1003936.s001.jpg]

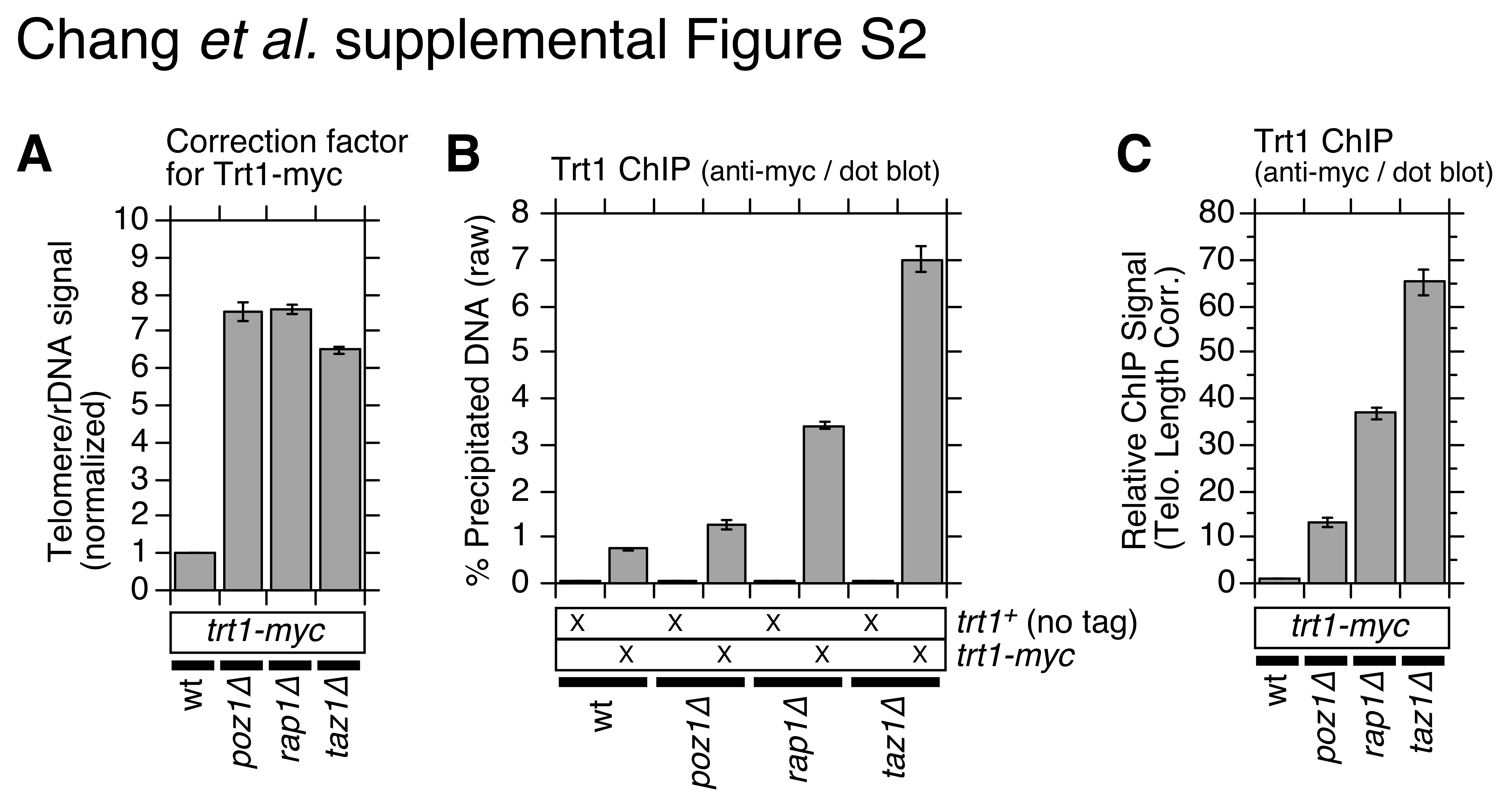

Supplement: Figure S2 — Analysis of Trt1TERT recruitment to telomeres by dot blot-based asynchronous ChIP assays with telomeric DNA probe. (A) Telomere correction factors for Trt1-myc strains were established by determining telomere/rDNA hybridization signal ratios relative to wt cells. Telomere correction factors for other epitope tagged strains are shown in Supplementary Table S1. (B) Raw % precipitated DNA values for dot blot-based Trt1-myc ChIP assays for the indicated genotypes. (C) Telomere length corrected ChIP data for Trt1-myc. (See Materials and Methods section for details.) Error bars correspond to SEM. (JPG) [file pgen.1003936.s002.jpg]

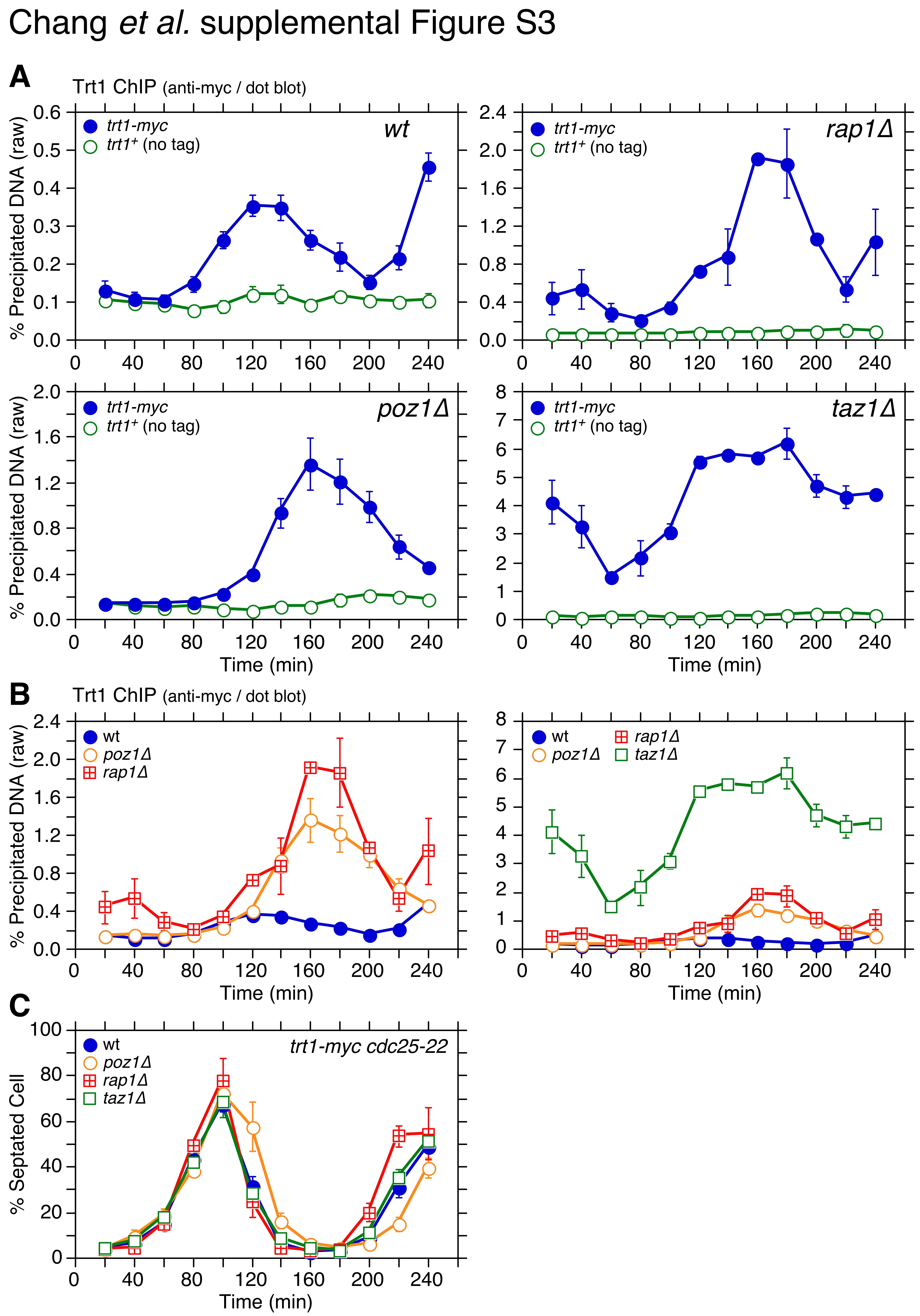

Supplement: Figure S3 — Raw data of dot blot-based cell cycle ChIP assays for Trt1TERT. (A, B) Cell cycle ChIP assays were performed with cdc25-22 synchronized cell cultures for wt, poz1Δ, rap1Δ or taz1Δ cells, and % precipitated DNA was determined by hybridization of a telomeric probe to dot blotted input and ChIP samples. (C) % septated cells were measured to monitor cell cycle progression of cdc25-22 synchronized cell cultures for the indicated genotypes. Error bars correspond to SEM. (JPG) [file pgen.1003936.s003.jpg]

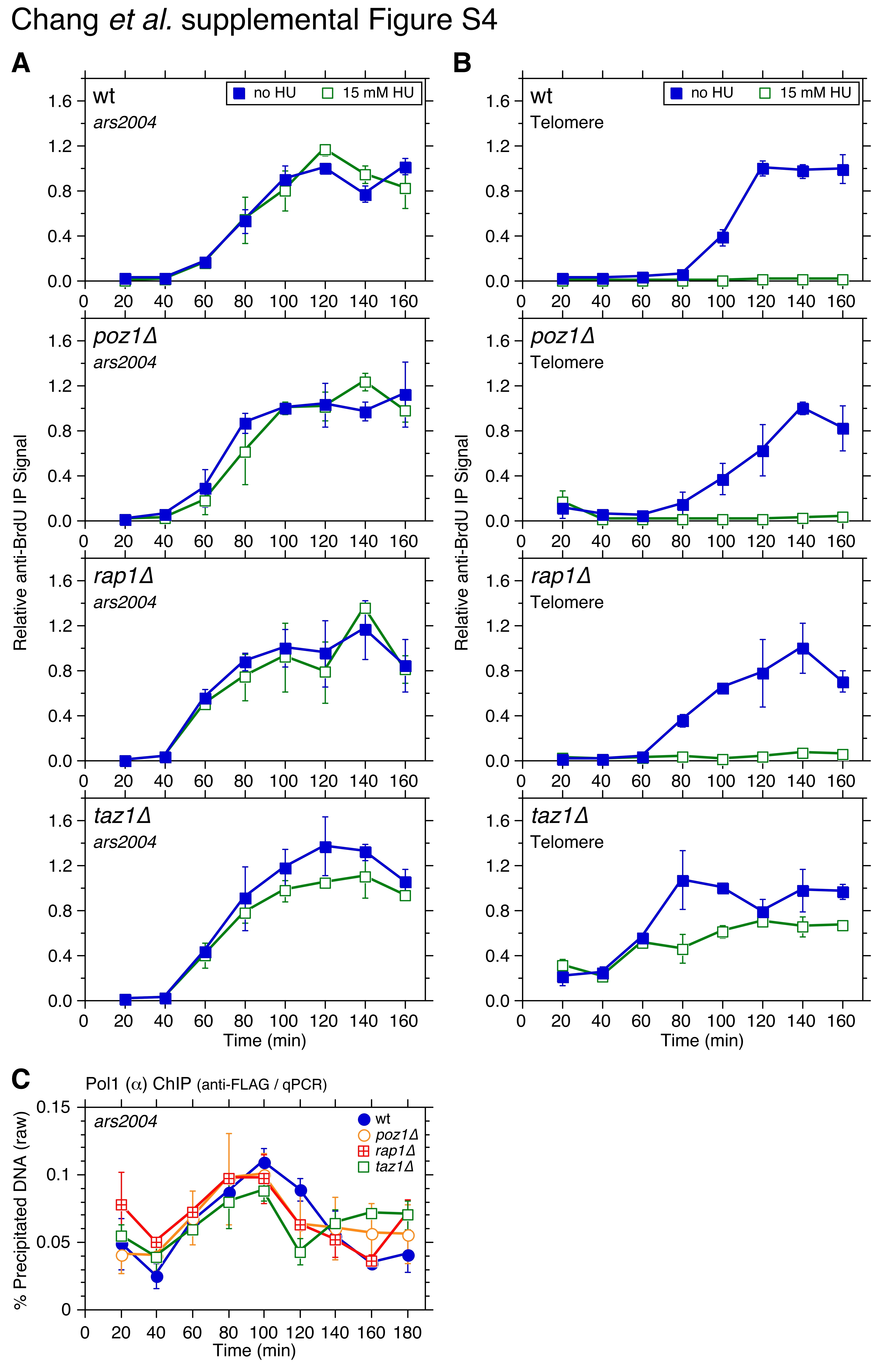

Supplement: Figure S4 — DNA replication timing monitored by incorporation of BrdU in cdc25-22 synchronized cells for (A) ars2004 and (B) telomeres [25]. BrdU incorporation at telomeres is inhibited by addition of 15 mM HU for wt, poz1Δ and rap1Δ cells but not for taz1Δ cells. BrdU is incorporated into ars2004 with similar kinetics in the presence or absence of HU for all genetic backgrounds tested. (C) Pol1 (α) showed similar timing of recruitment to ars2004 in all genetic backgrounds tested. Error bars correspond to SEM. (JPG) [file pgen.1003936.s004.jpg]

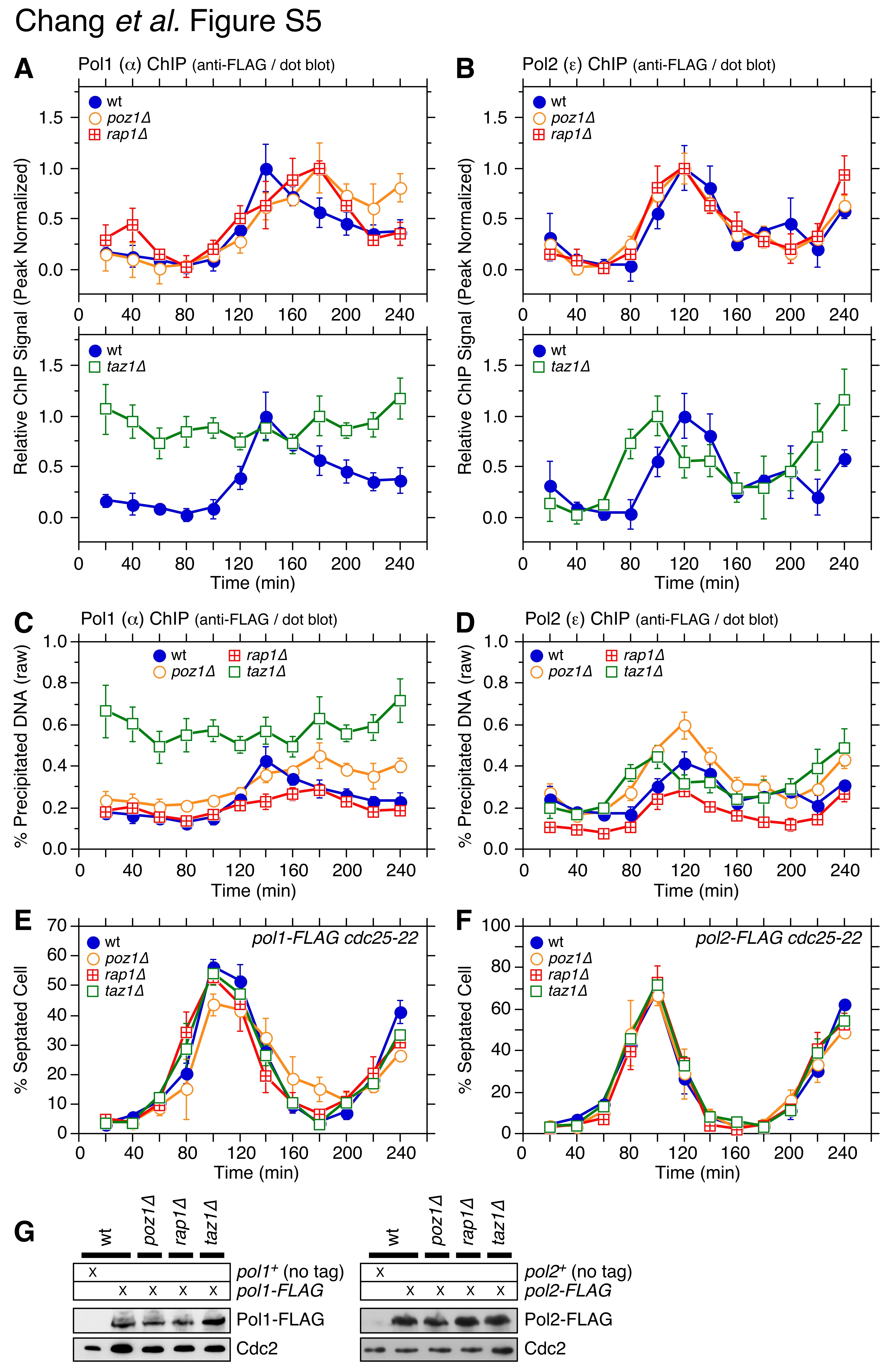

Supplement: Figure S5 — Cell cycle ChIP assays for DNA polymerases. (A, B) Peak normalized cell cycle ChIP data for Pol1 (α) (A) and Pol2 (ε) (B). For Pol2 (ε), Student's t-test found a statistically significant difference in telomere binding at 80 min (p = 0.03) for wt vs. taz1Δ cells. (C, D) Raw data of dot blot-based cell cycle ChIP assays for Pol1 (α) (C) and Pol2 (ε) (D), performed with cdc25-22 synchronized cell cultures and telomeric DNA probe. (E, F) % septated cells were measured to monitor cell cycle progression of cdc25-22 synchronized cell cultures for Pol1 (α) (E) and Pol2 (ε) (F) ChIP assays. Error bars correspond to SEM. (G) Anti-FLAG western blot analysis indicated comparable expression levels in different genetic backgrounds for both Pol1 (α) and Pol2 (ε). Cdc2 western blot served as loading control. (JPG) [file pgen.1003936.s005.jpg]

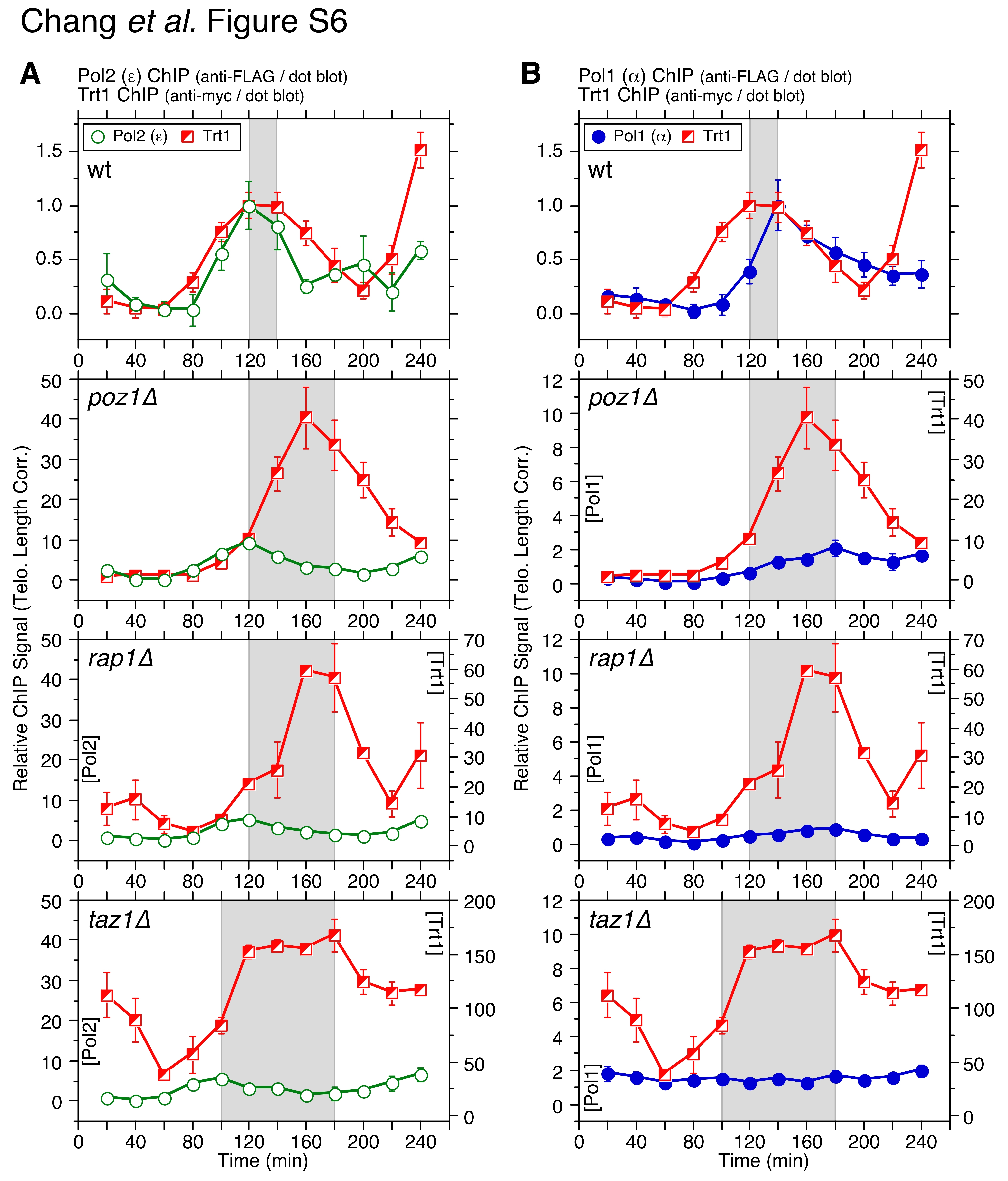

Supplement: Figure S6 — Comparison of cell cycle ChIP data among DNA polymerases and Trt1TERT. Comparison of telomere length corrected ChIP data between Pol2 (ε) and Trt1 (A) or Pol1 (α) and Trt1 (B) in indicated genomic backgrounds. For explanation of shaded areas in graphs, see Figure 2 legend. Error bars correspond to SEM. (JPG) [file pgen.1003936.s006.jpg]

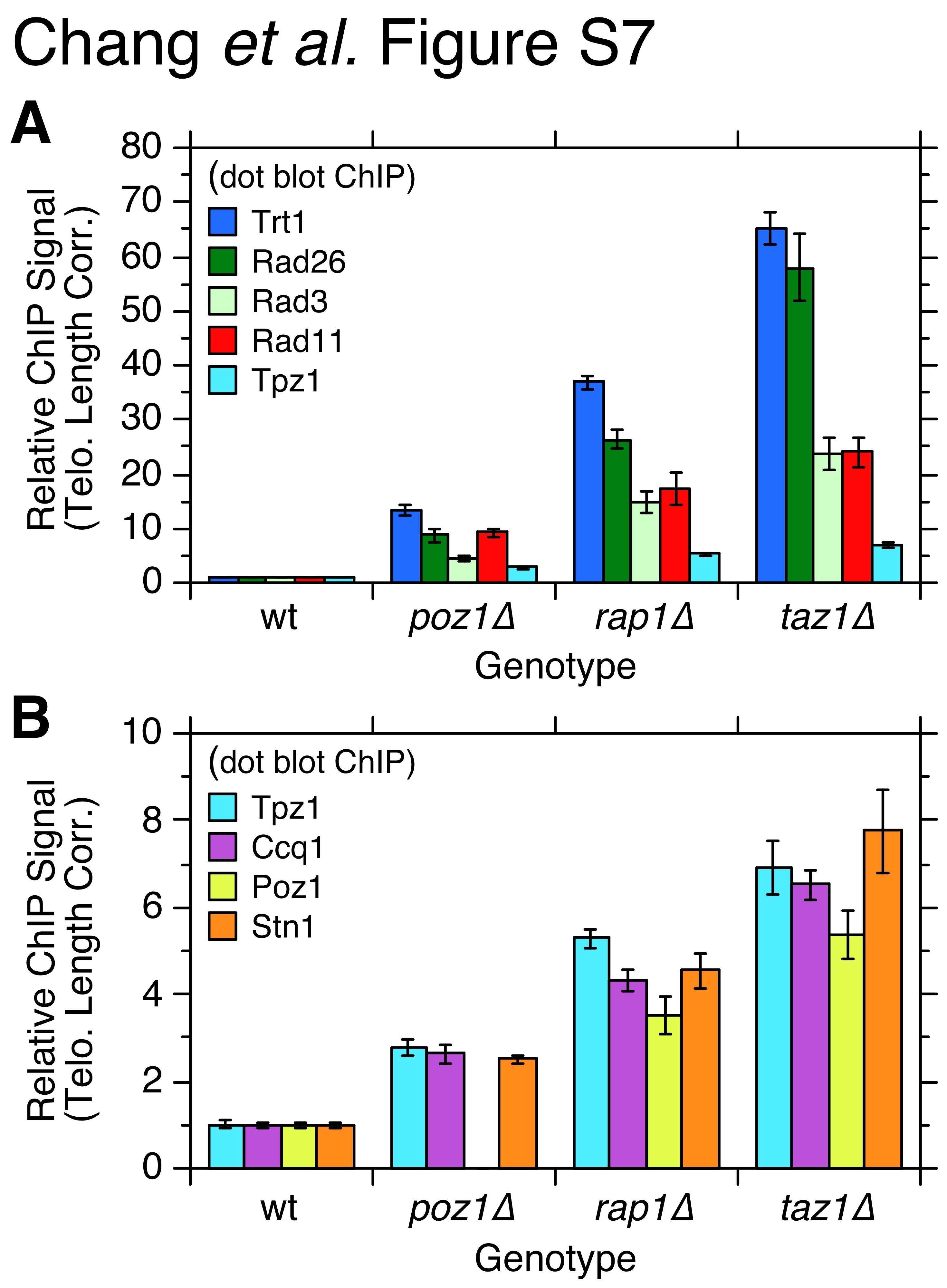

Supplement: Figure S7 — Telomere length corrected dot blot-based asynchronous ChIP data for indicated proteins in wt, poz1Δ, rap1Δ and taz1Δ cells. (A) Raw ChIP data from Supplementary Figures S8, S9 for Trt1TERT, Rad26ATRIP, Rad3ATR, Rad11RPA and Tpz1 were corrected for telomere length and normalized to wt cells. Compared to wt cells, poz1Δ, rap1Δ, and taz1Δ cells all showed statistically significant increases in telomere association for Trt1TERT (p<1.2×10^−11^), Rad26ATRIP (p<6.4×10^−4^), Rad3ATR (p<0.047 for poz1Δ while p<1.8×10^−5^ for rap1Δ and taz1Δ), Rad11RPA (p<1.6×10^−3^) and Tpz1 (p<3.2×10^−7^). (B) Raw ChIP data from Supplementary Figure S9 for Tpz1, Ccq1, Poz1 and Stn1 were corrected for telomere length and normalized to wt cells. Compared to wt cells, poz1Δ, rap1Δ, and taz1Δ cells all showed statistically significant increases in telomere association for Ccq1 (p<1.8×10^−4^), Poz1 (p<1.5×10^−5^) and Stn1 (p<1.1×10^−5^). Error bars correspond to SEM. (JPG) [file pgen.1003936.s007.jpg]

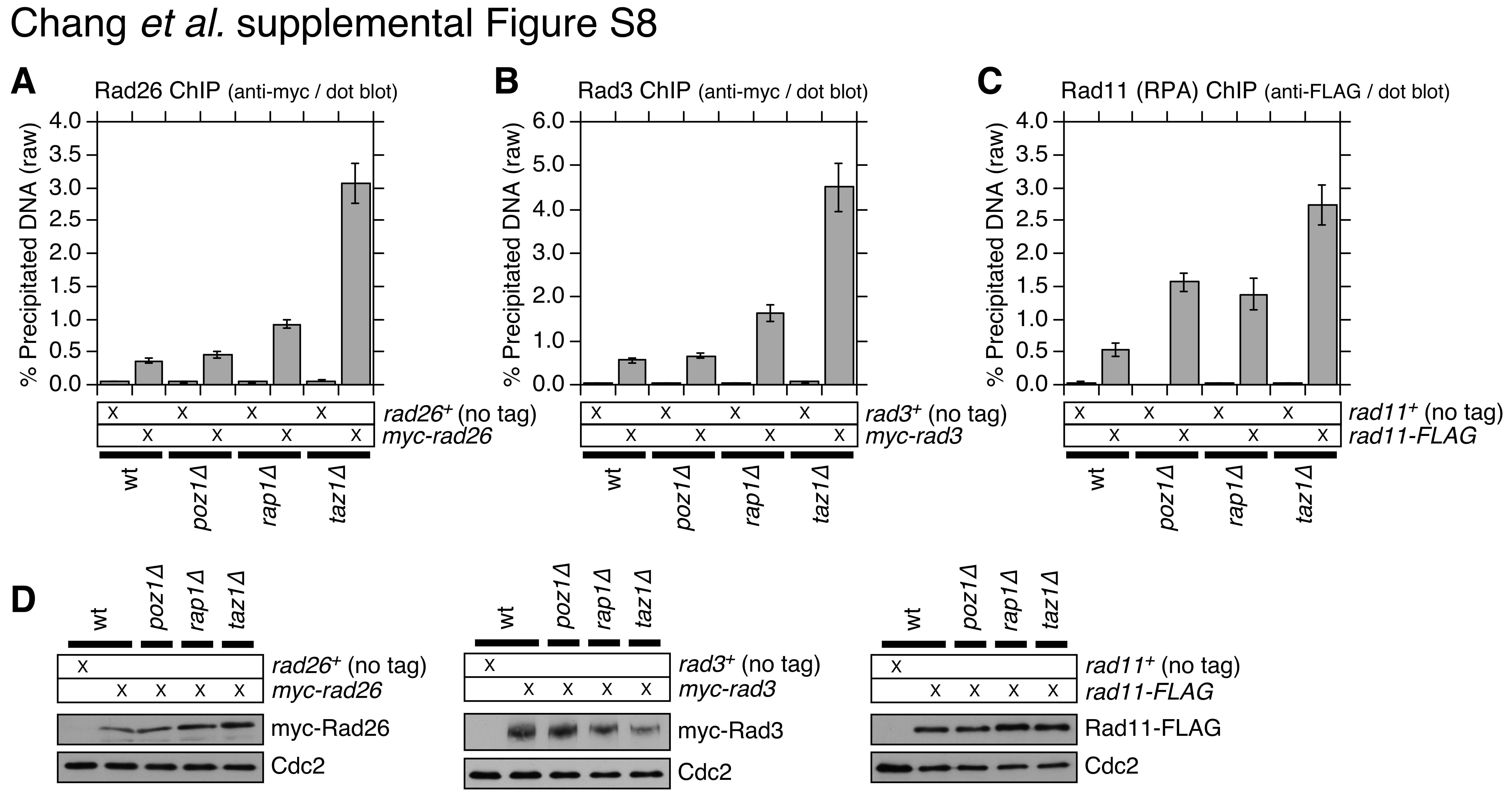

Supplement: Figure S8 — Raw % precipitated DNA against input DNA for Rad26ATRIP (A), Rad3ATR (B) and Rad11RPA (C) obtained by dot blot-based asynchronous ChIP assays with telomeric DNA probe. Error bars correspond to SEM. (D) Anti-myc (Rad26 and Rad3) and anti-FLAG (Rad11) western blot analysis indicated comparable expression levels in different genetic backgrounds. Cdc2 western blot served as a loading control. (JPG) [file pgen.1003936.s008.jpg]

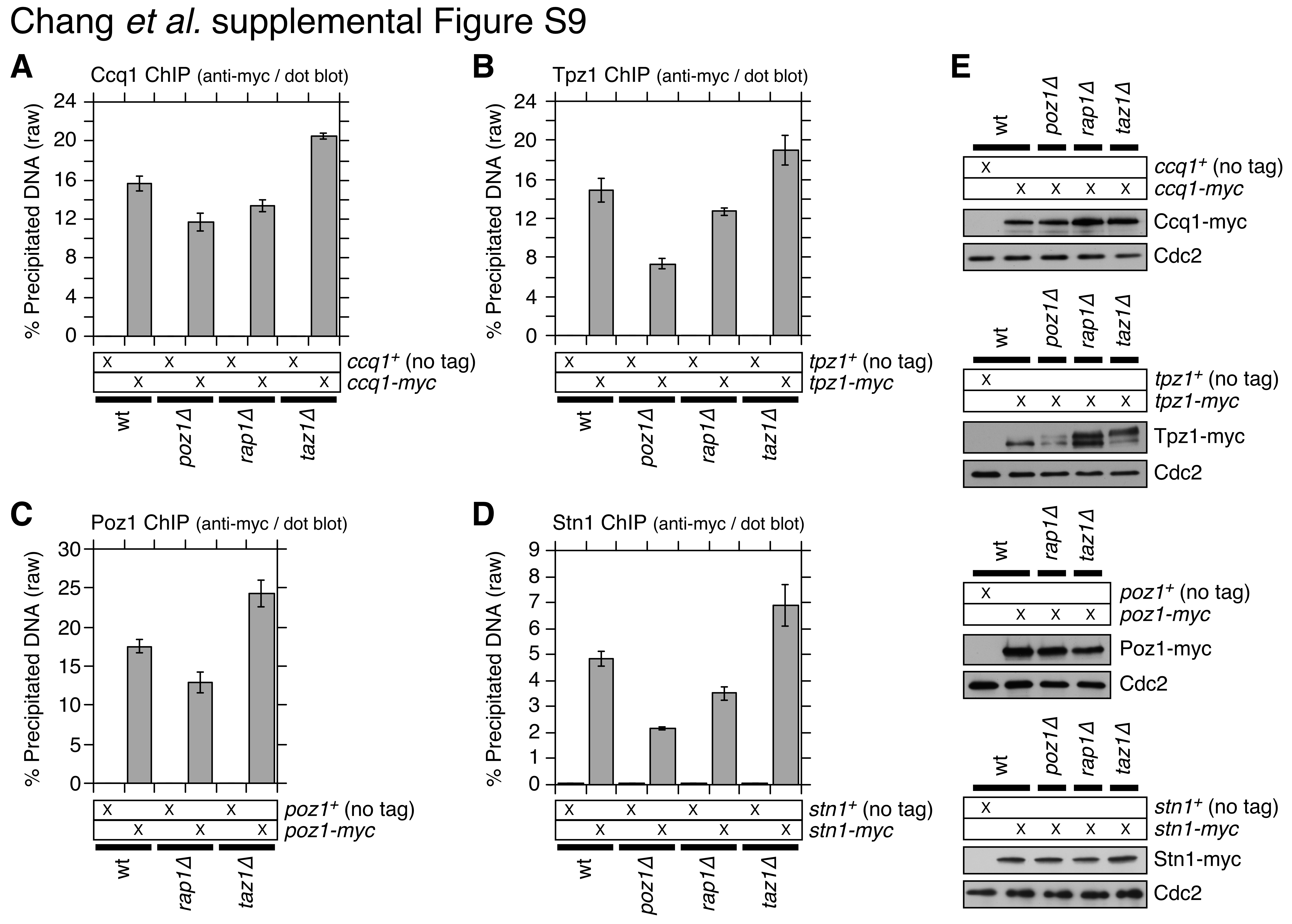

Supplement: Figure S9 — Raw % precipitated DNA against input DNA for Ccq1 (A), Tpz1 (B), Poz1 (C) and Stn1 (D) obtained by dot blot-based asynchronous ChIP assays with telomeric DNA probe. Error bars correspond to SEM. (E) Anti-myc western blot analyses indicated comparable expression levels for all proteins in different genetic backgrounds. Cdc2 western blot served as a loading control. (JPG) [file pgen.1003936.s009.jpg]

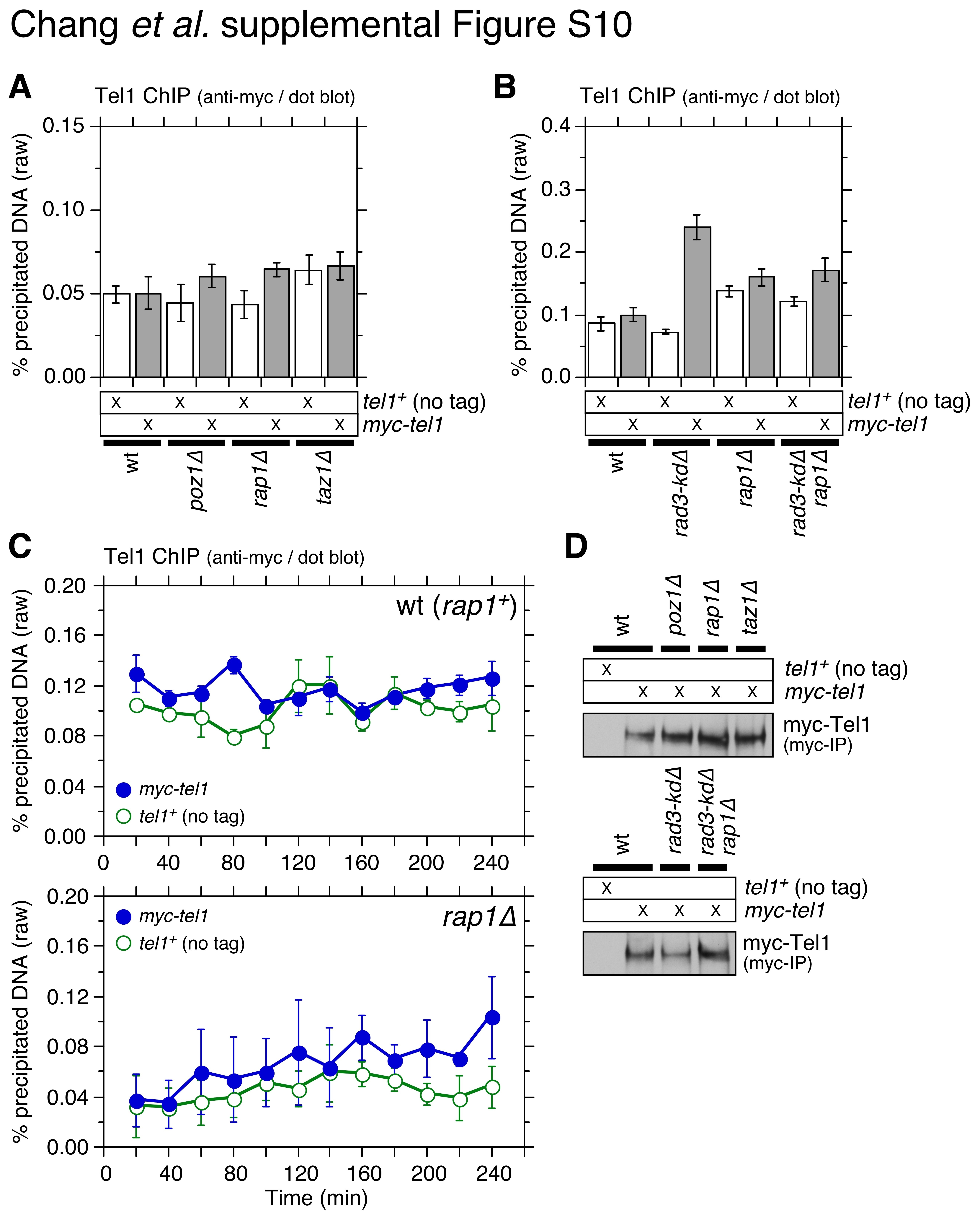

Supplement: Figure S10 — Tel1ATM does not show increased binding to telomeres in poz1Δ, rap1Δ and taz1Δ cells. (A, B) Raw % precipitated DNA against input DNA for Tel1ATM obtained by dot blot-based asynchronous ChIP assays with telomeric DNA probe. For (A), none of the strains showed statistically significant Tel1ATM binding over no tag controls. For (B), only rad3-kdΔ cells [57] showed statistically significant Tel1ATM binding over no tag control (p = 6.0×10^−4^). (C) Raw data of dot blot-based cell cycle ChIP assays for Tel1ATM in wt or rap1Δ cells, performed with cdc25-22 synchronized cell cultures and telomeric DNA probe. Among all time points, only wt cells at 80 min showed statistically significant Tel1ATM binding over no tag control (p = 4.0×10^−3^). Error bars correspond to SEM. (D) While myc-Tel1 expressed from its endogenous promoter could not be detected in whole cell extracts, comparable amounts of Tel1ATM were immunoprecipitated (IP) with anti-myc antibody in different genetic backgrounds. (JPG) [file pgen.1003936.s010.jpg]

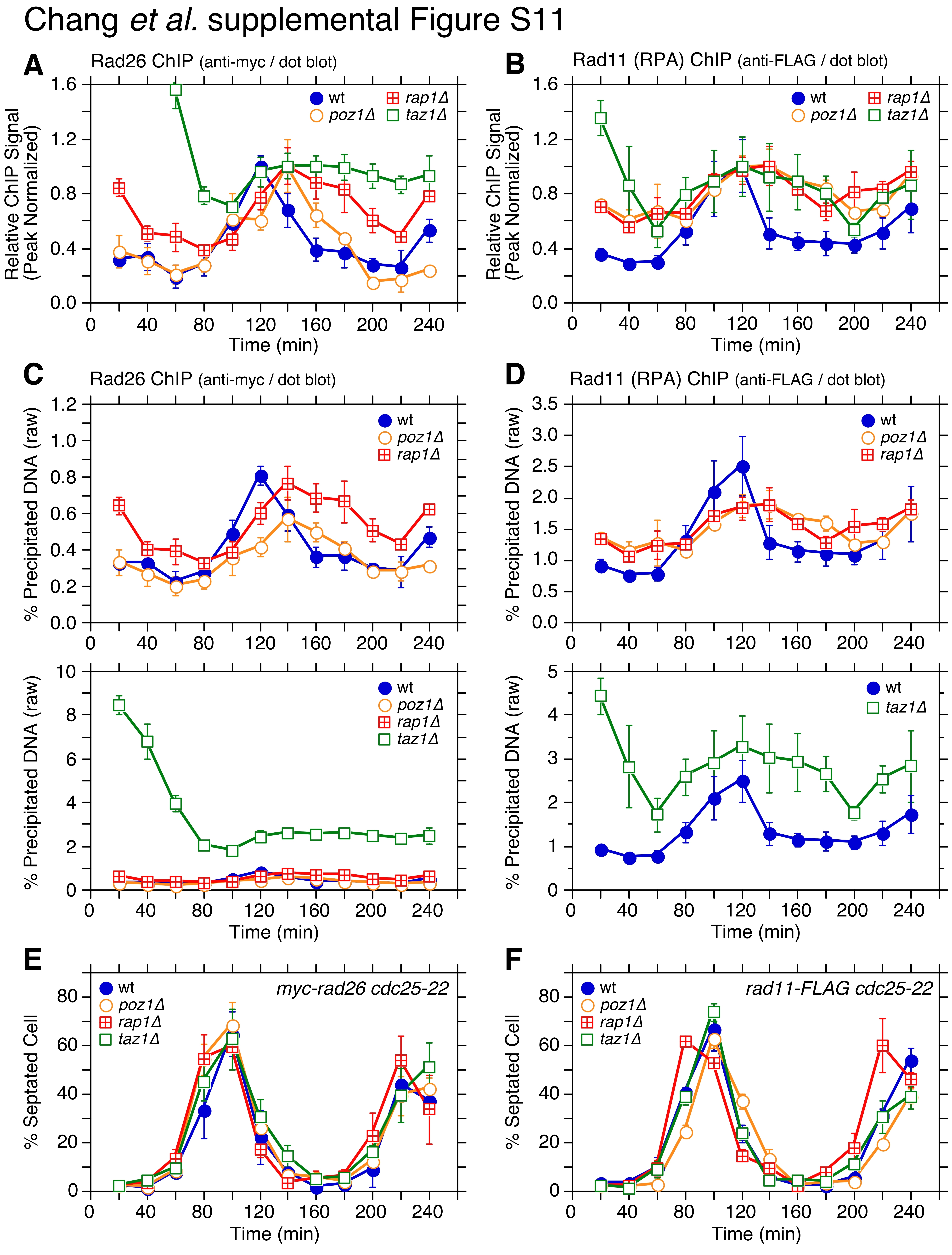

Supplement: Figure S11 — Cell cycle ChIP assays for Rad26ATRIP and Rad11RPA. (A, B) Peak normalized cell cycle ChIP data for Rad26 (A) and Rad11 (B). (C, D) Raw data of dot blot-based cell cycle ChIP assays for Rad26 (C) and Rad11 (D), performed with cdc25-22 synchronized cell cultures and telomeric DNA probe. (E, F) % septated cells were measured to monitor cell cycle progression of cdc25-22 synchronized cell cultures for Rad26 (E) and Rad11 (F) ChIP assays. Error bars correspond to SEM. (JPG) [file pgen.1003936.s011.jpg]

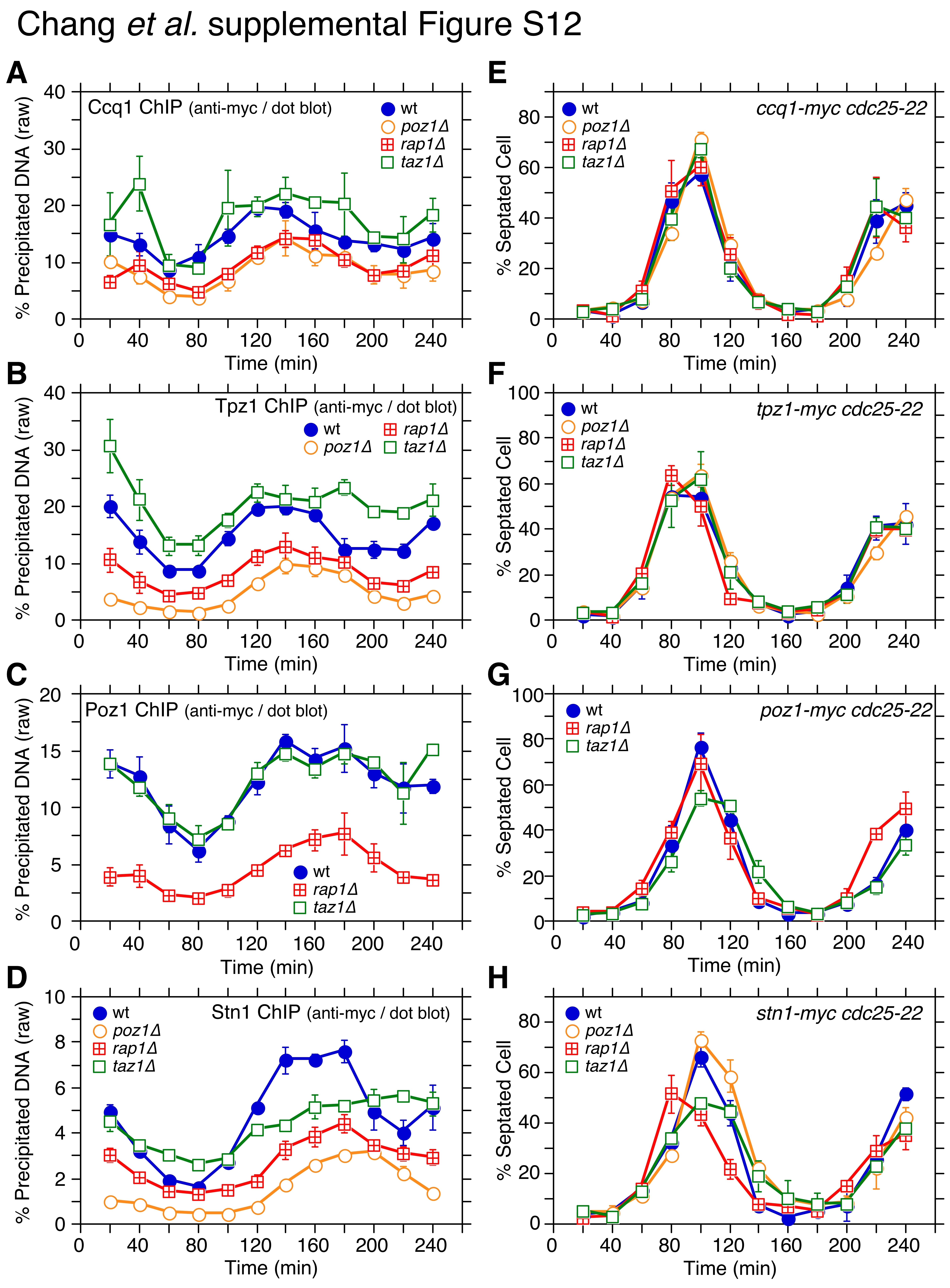

Supplement: Figure S12 — Cell cycle ChIP assays for shelterin subunits and Stn1. (A–D) Raw data of dot blot-based cell cycle ChIP assays for Ccq1 (A), Tpz1 (B), Poz1 (C) and Stn1 (D), performed with cdc25-22 synchronized cell cultures and telomeric DNA probe. (E–H) % septated cells were measured to monitor cell cycle progression of cdc25-22 synchronized cell cultures for Ccq1 (E), Tpz1 (F), Poz1 (G) and Stn1 (H) ChIP assays. Error bars correspond to SEM. (JPG) [file pgen.1003936.s012.jpg]

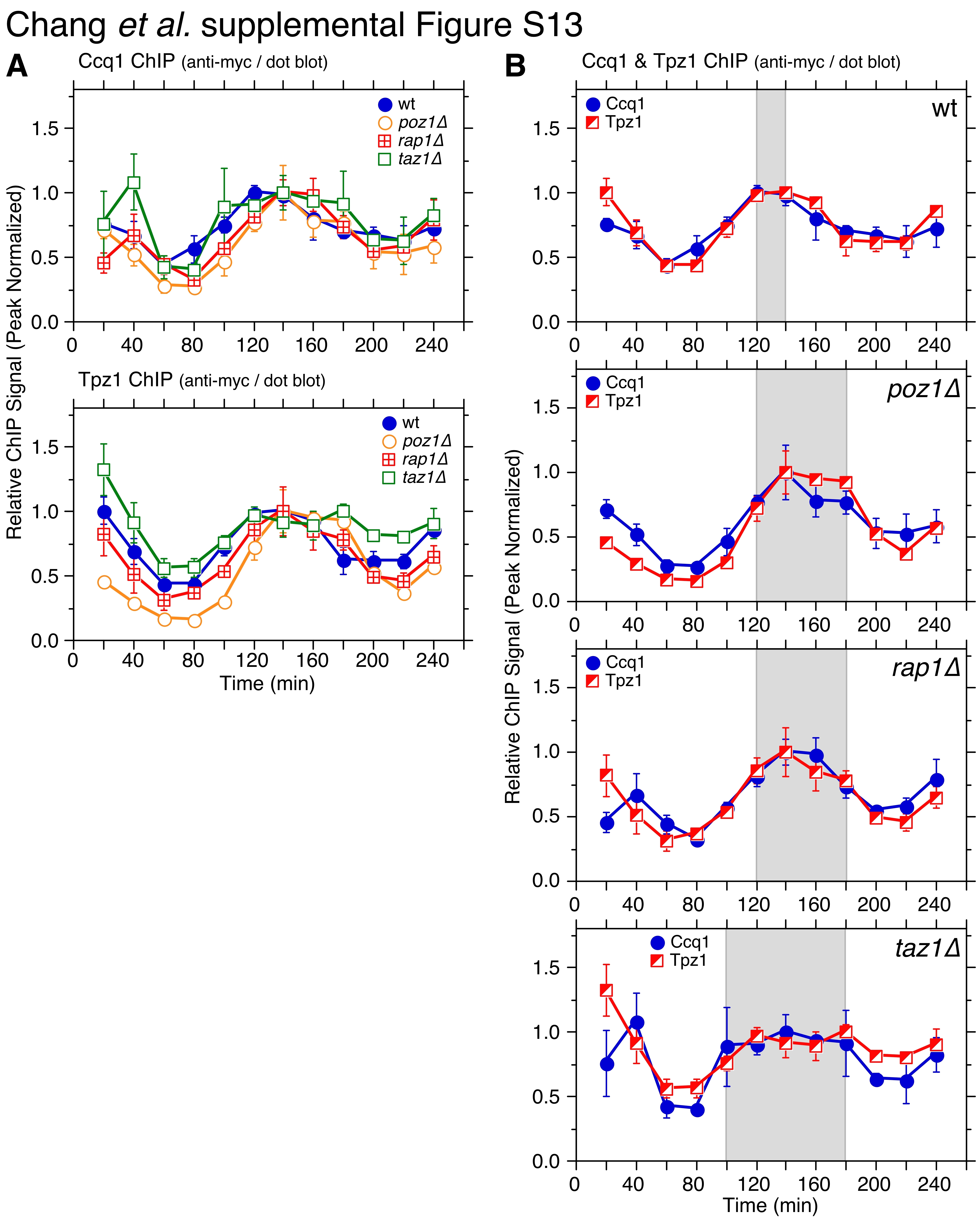

Supplement: Figure S13 — Comparison of peak normalized cell cycle ChIP data between Ccq1 and Tpz1. (A) Peak normalized ChIP data for either Ccq1 or Tpz1 in different genetic backgrounds were plotted to compare changes in temporal association with telomeres. (B) Comparison of peak normalized ChIP data indicated that temporal changes in telomere association for Ccq1 and Tpz1 are nearly identical in all genetic backgrounds tested. For explanation of shaded areas in graphs, see Figure 2 legend. Error bars correspond to SEM. (JPG) [file pgen.1003936.s013.jpg]

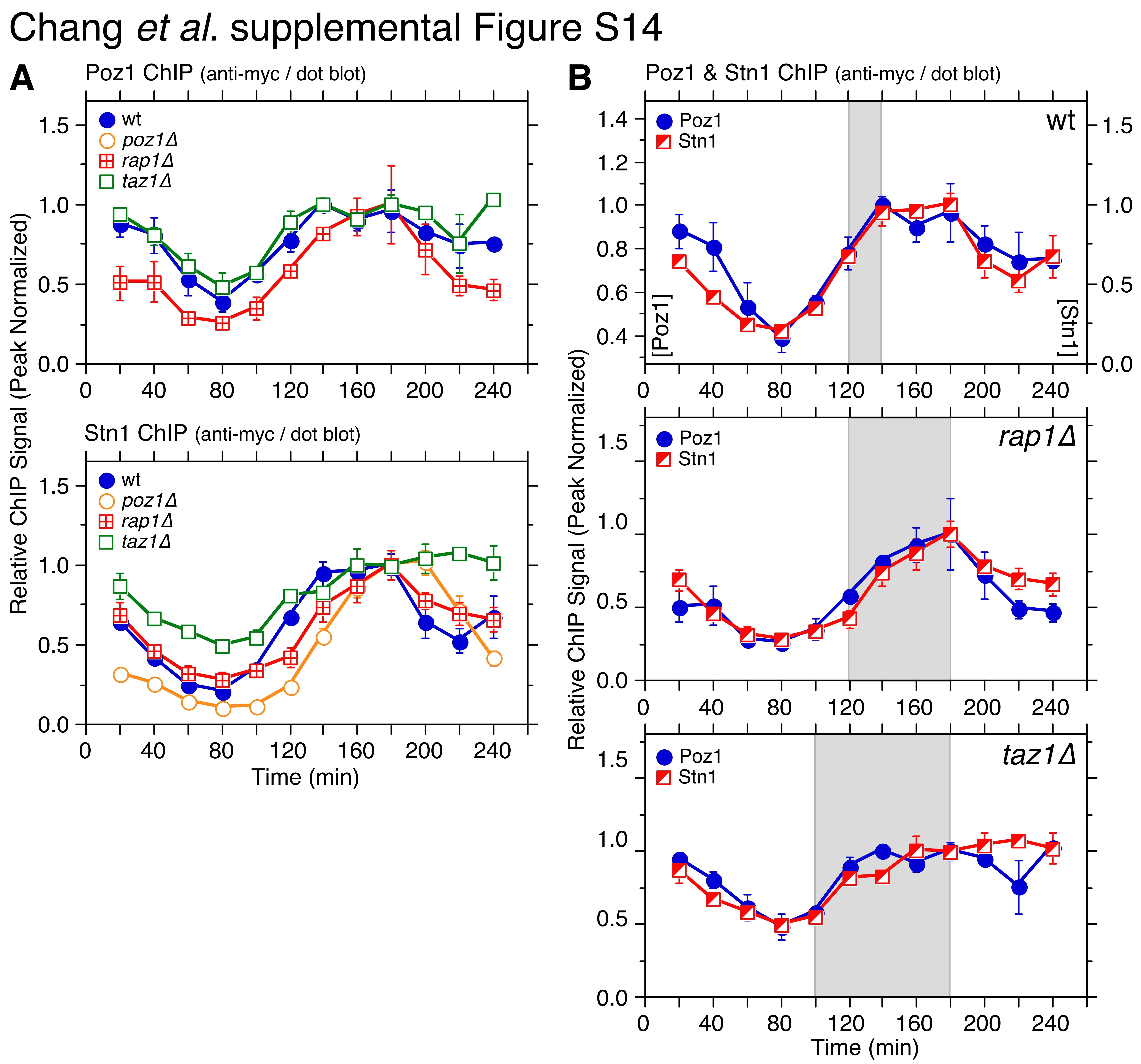

Supplement: Figure S14 — Comparison of peak normalized cell cycle ChIP data between Poz1 and Stn1. (A) Peak normalized ChIP data for either Poz1 or Stn1 in different genetic backgrounds were plotted to compare changes in temporal association with telomeres. (B) Comparison of peak normalized ChIP data indicated that temporal changes in telomere association for Poz1 and Stn1 are nearly identical in wt, rap1Δ and taz1Δ cells. For explanation of shaded areas in graphs, see Figure 2 legend. Error bars correspond to SEM. (JPG) [file pgen.1003936.s014.jpg]

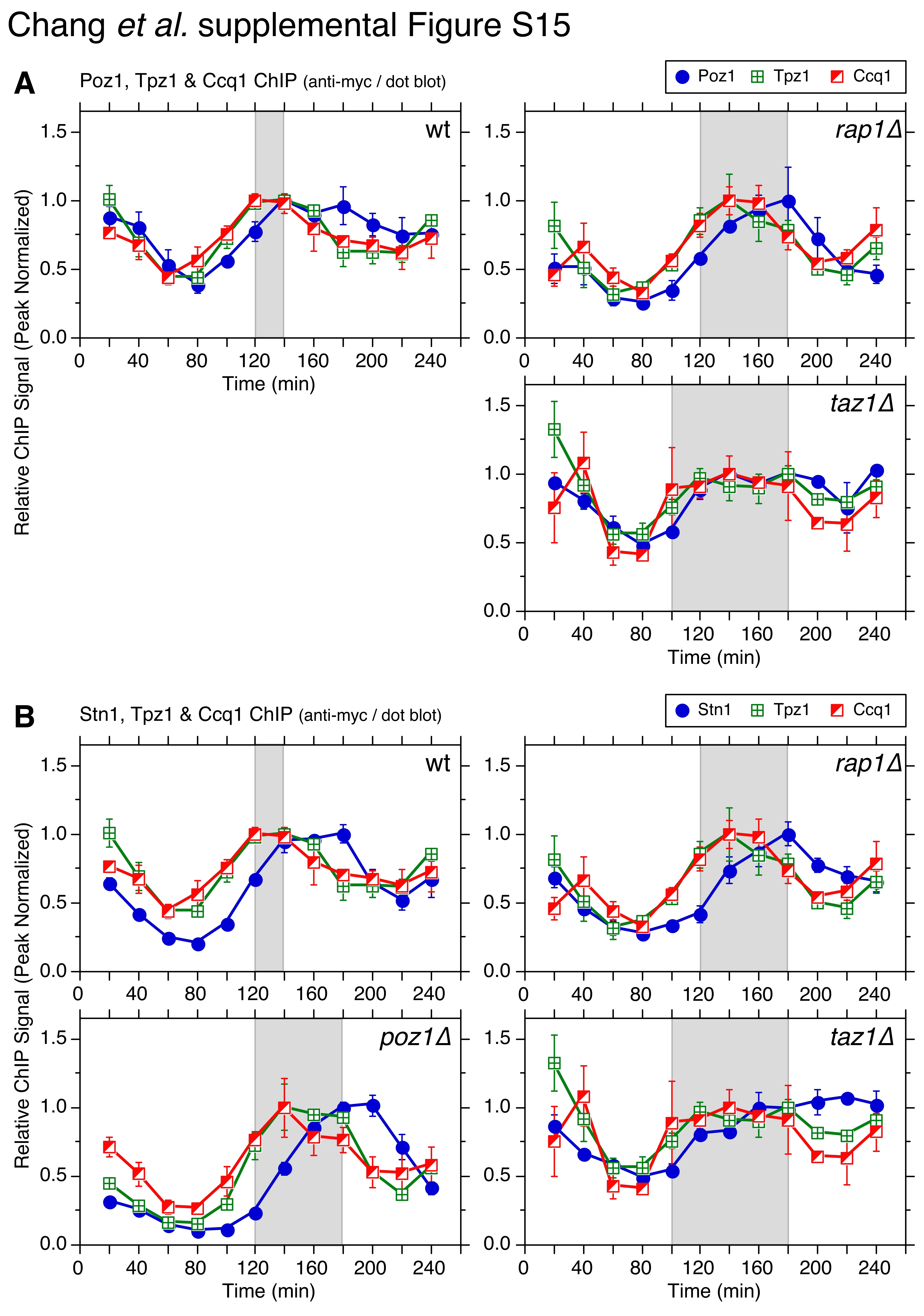

Supplement: Figure S15 — Comparison of cell cycle ChIP data among Ccq1, Tpz1, Poz1 and Stn1. (A) Comparison of peak normalized ChIP data for Poz1, Tpz1 and Ccq1 in wt, rap1Δ and taz1Δ cells. For Tpz1 vs. Poz1, Student's t-test found p = 0.053 at 120 min (94.7% confidence level) for wt cells, and p = 0.058 at 80 min (94.2% confidence level) and p = 0.09 at 100 min (91% confidence level) for rap1Δ cells. For Ccq1 vs. Poz1, Student's t-test found p = 0.045 at 100 min (95.5% confidence level) and p = 0.071 at 120 min (92.9% confidence level) for wt cells, and p = 0.082 at 100 min (91.8% confidence level) for rap1Δ cells. (B) Comparison of peak normalized ChIP data for Stn1, Tpz1 and Ccq1 in wt, poz1Δ, rap1Δ and taz1Δ cells. For Tpz1 vs. Stn1, differences were statistically significant at 60–120 min for wt cells (p<0.03), at 100, 120, 200 and 220 min for poz1Δ cells (p<0.04), and at 100, 120, 200 min for rap1Δ cells (p<0.01). For Ccq1 vs. Stn1, differences were statistically significant at 100, 120 and 180 min for wt cells (p<0.03), at 80 and 120 min for poz1Δ cells (p<0.04), and at 100, 120, 200 min for rap1Δ cells (p<0.02). For explanation of shaded areas in graphs, see Figure 2 legend. Error bars correspond to SEM. (JPG) [file pgen.1003936.s015.jpg]

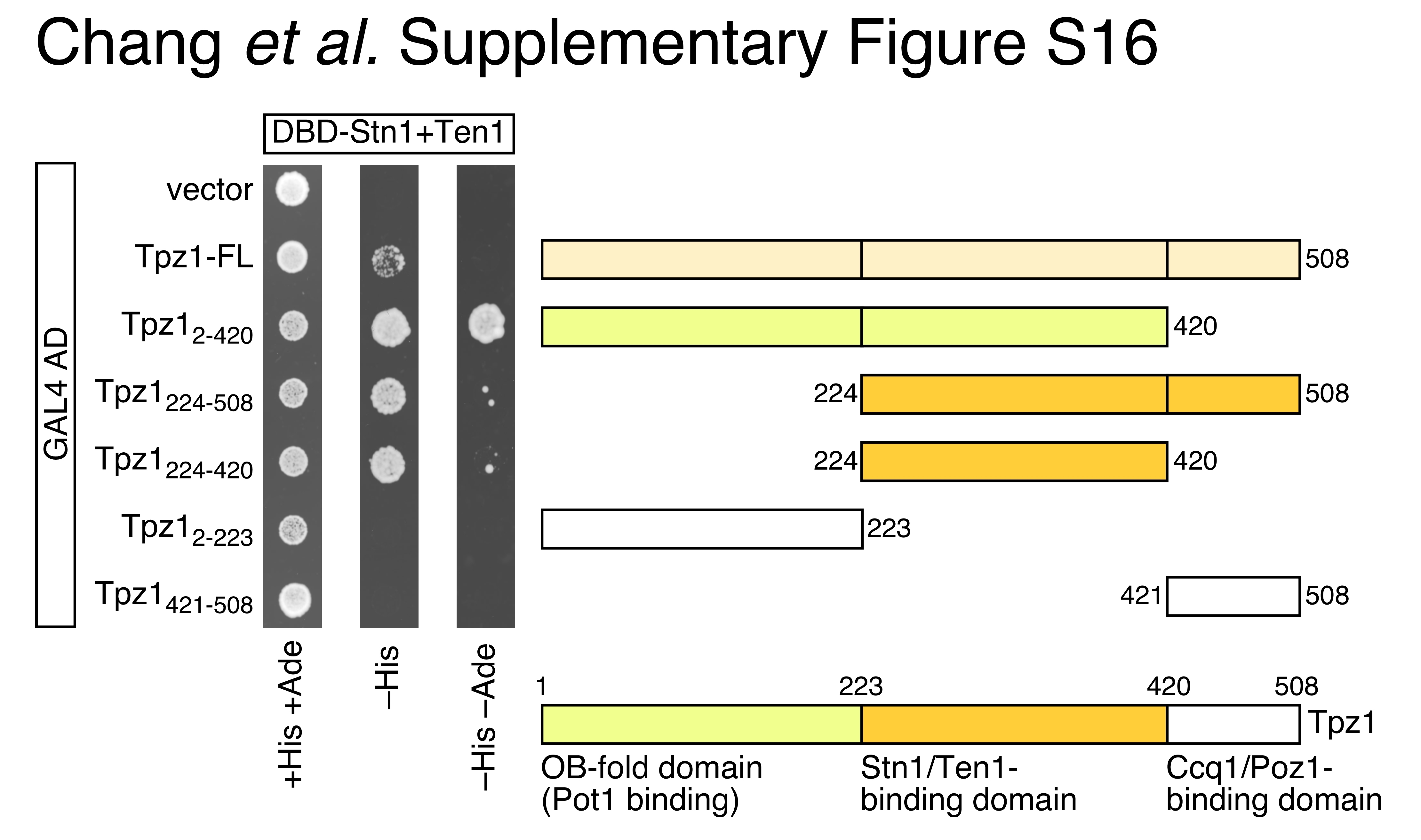

Supplement: Figure S16 — Yeast 3-hybrid assay to monitor interaction between Tpz1 and Stn1-Ten1. Various truncation constructs of Tpz1 were tested for interaction with Stn1 and Ten1. Based on cell growth on –His selection plate, a Tpz1 fragment containing amino acids 224–420 was the smallest Tpz1 construct that retained interaction with Stn1 and Ten1. Based on growth on –His –Ade plate, a Tpz1 fragment containing amino acids 2–420 showed strongest interaction with Stn1 and Ten1. (JPG) [file pgen.1003936.s016.jpg]

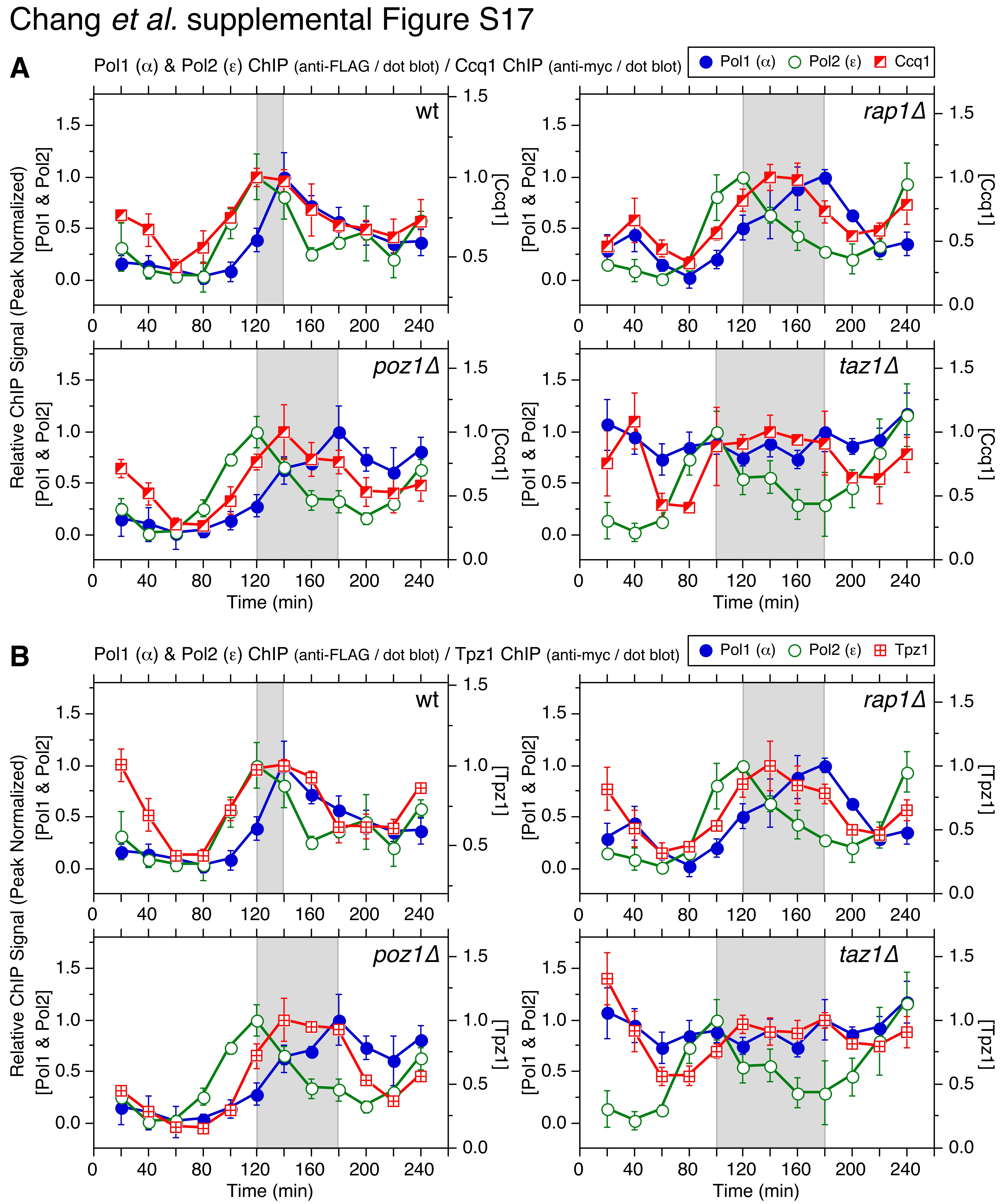

Supplement: Figure S17 — Comparison of cell cycle ChIP data among DNA polymerases, Ccq1 and Tpz1. Comparison of peak normalized ChIP data for Pol1 (α), Pol2 (ε) and Ccq1 (A) or Pol1 (α), Pol2 (ε) and Tpz1 (B) in wt, poz1Δ, rap1Δ, and taz1Δ cells. For explanation of shaded areas in graphs, see Figure 2 legend. Error bars correspond to SEM. (JPG) [file pgen.1003936.s017.jpg]

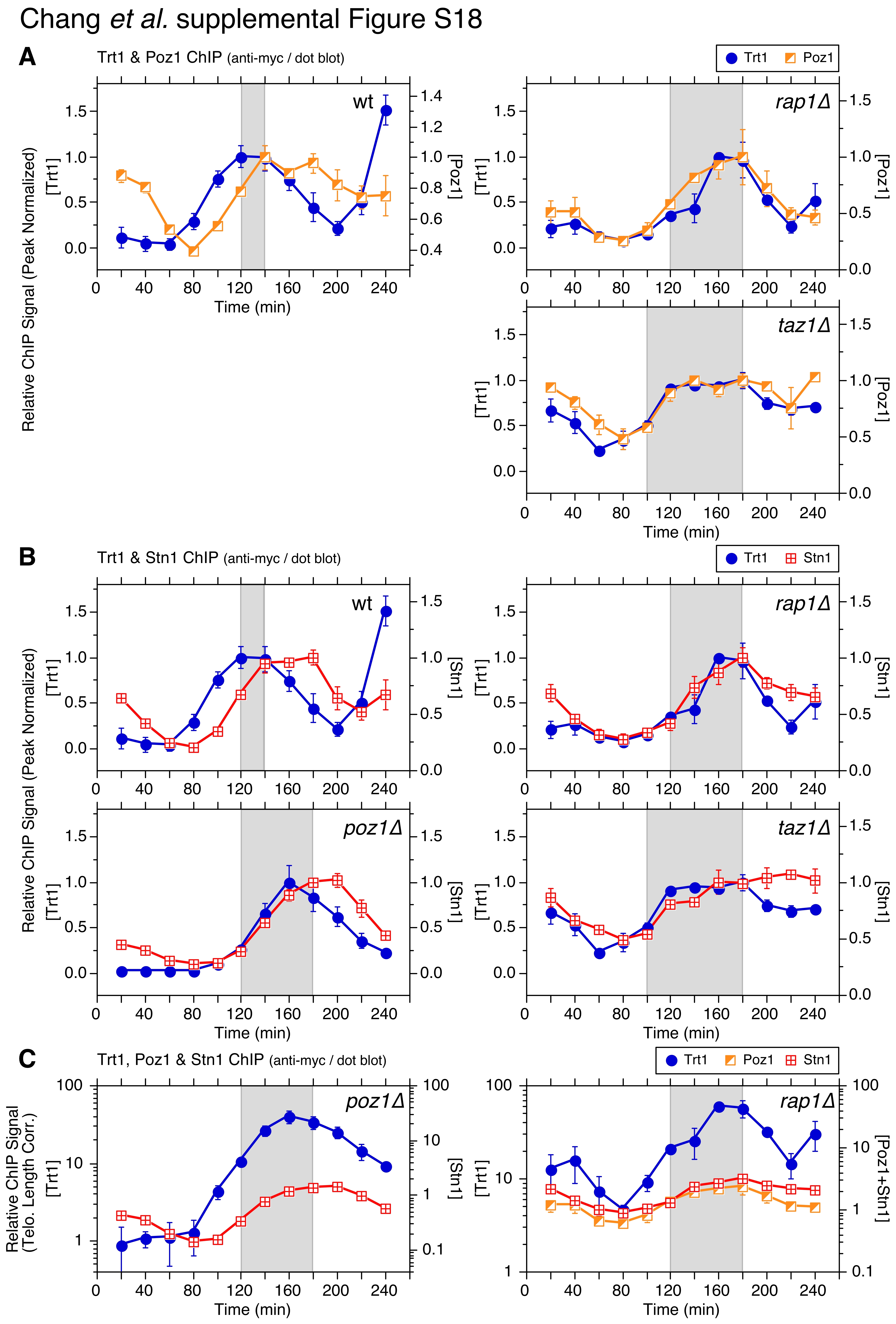

Supplement: Figure S18 — Comparison of cell cycle ChIP data among Trt1TERT, Poz1 and Stn1. Comparison of peak normalized ChIP data between Trt1 and Poz1 (A) or Trt1 and Stn1 (B) for indicated genomic backgrounds. (C) Comparison of peak normalized ChIP data among Trt1, Poz1 and Stn1 in poz1Δ or rap1Δ, plotted on log scale. For explanation of shaded areas in graphs, see Figure 2 legend. Error bars correspond to SEM. (JPG) [file pgen.1003936.s018.jpg]

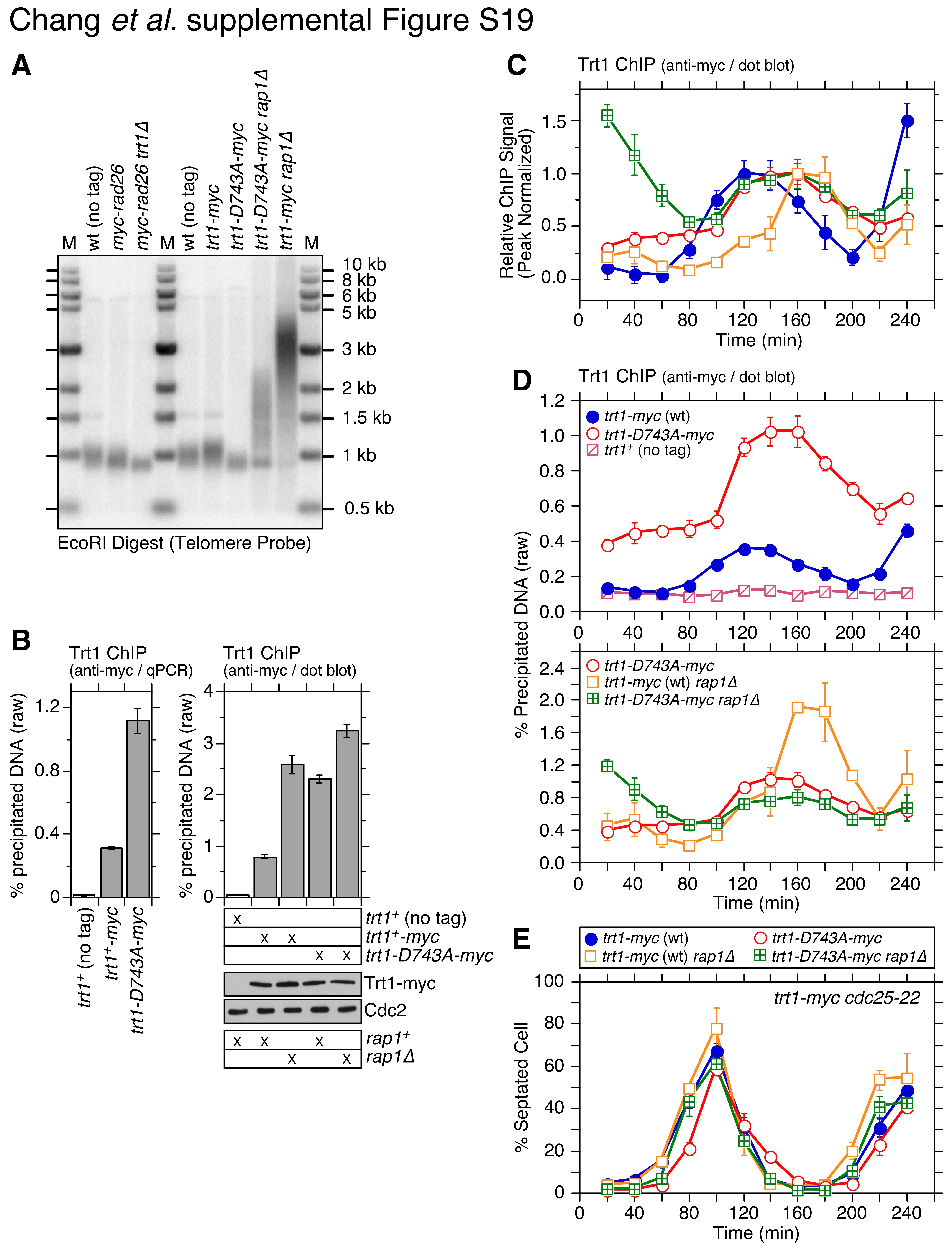

Supplement: Figure S19 — Cell cycle ChIP assays for catalytically dead Trt1-D743A. (A) Telomere length analysis for indicated strains used in ChIP analysis. Genomic DNA was prepared from early generation strains. After digestion with EcoRI, DNA was fractionated on a 1% agarose gel and processed for Southern blot analysis with a telomere probe. (B) Raw % precipitated DNA against input DNA for Trt1TERT obtained by real-time quantitative PCR analysis (left) or dot blot-based asynchronous ChIP assays with telomeric DNA probe (right). Trt1-D743A showed a statistically significant increase in telomere association compared to wt Trt1TERT (p = 5.4×10^−5^) for PCR-based ChIP assay, independently confirming our conclusion from telomere-length corrected dot blot-based ChIP assay (Figure 6B). Anti-myc western blot analysis indicated comparable expression levels of Trt1 in different genetic backgrounds. Cdc2 western blot served as a loading control. (C) Peak normalized cell cycle ChIP data for wt or catalytically dead Trt1TERT in rap1+ or rap1Δ cells. (D) Raw data of dot blot-based cell cycle ChIP assays for Trt1TERT, performed with cdc25-22 synchronized cell cultures and telomeric DNA probe. (E) % septated cells were measured to monitor cell cycle progression of cdc25-22 synchronized cell cultures for Trt1TERT ChIP assays. Error bars correspond to SEM. (JPG) [file pgen.1003936.s019.jpg]

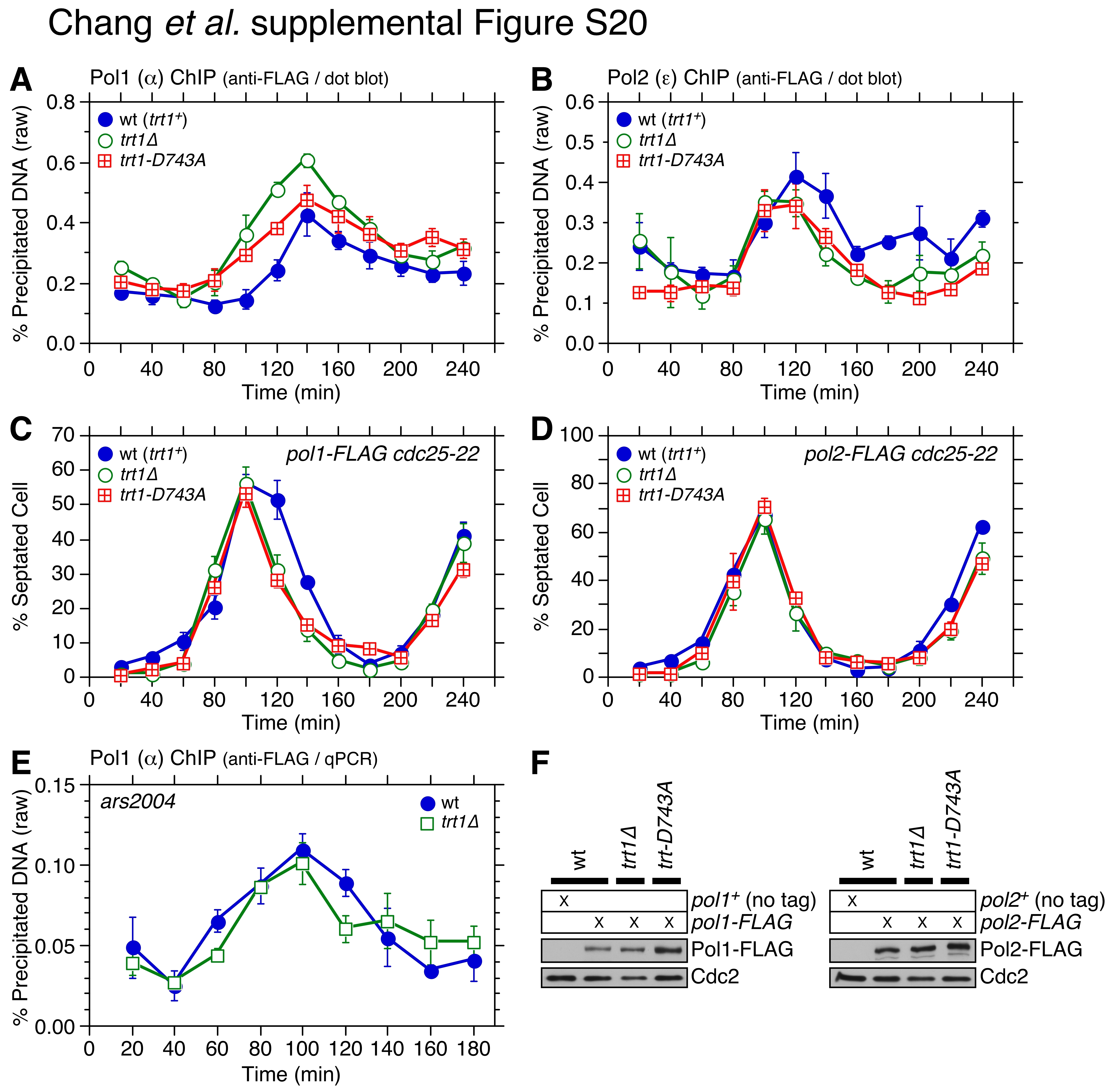

Supplement: Figure S20 — Cell cycle ChIP assays for DNA polymerases in trt1 mutants. (A, B) Raw data of dot blot-based cell cycle ChIP assays for Pol1 (α) (A) and Pol2 (ε) (B), performed with cdc25-22 synchronized cell cultures and telomeric DNA probe. (C, D) % septated cells were measured to monitor cell cycle progression of cdc25-22 synchronized cell cultures for Pol1 (α) (C) and Pol2 (ε) (D) ChIP assays. (E) Pol1 (α) showed similar timing of recruitment to ars2004 in wt and trt1Δ cells. Error bars correspond to SEM. (F) Anti-FLAG western blot analysis indicated comparable expression levels in different genetic backgrounds for both Pol1 and Pol2. Cdc2 western blot served as a loading control. (JPG) [file pgen.1003936.s020.jpg]

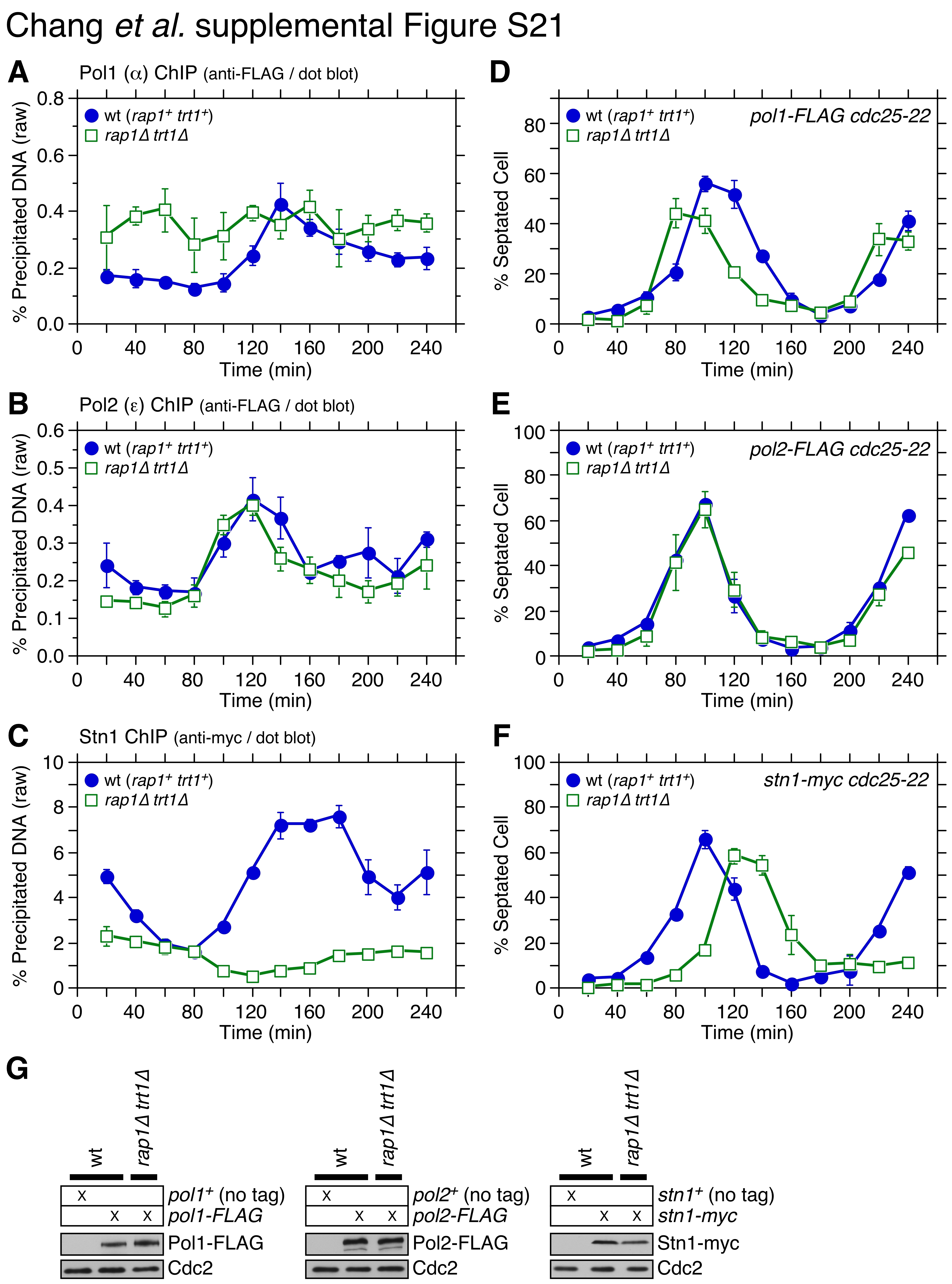

Supplement: Figure S21 — Cell cycle ChIP assays for DNA polymerases and Stn1 in rap1Δ trt1Δ cells. (A–C) Raw data of dot blot-based cell cycle ChIP assays for Pol1 (α) (A), Pol2 (ε) (B) and Stn1 (C), performed with cdc25-22 synchronized cell cultures and telomeric DNA probe. (D–F) % septated cells were measured to monitor cell cycle progression of cdc25-22 synchronized cell cultures for Pol1 (D), Pol2 (E) and Stn1 (F) ChIP assays. Error bars correspond to SEM. (G) Anti-FLAG (Pol1 and Pol2) and anti-myc (Stn1) western blot analyses indicated comparable expression levels in different genetic backgrounds for both Pol1 (α) and Pol2 (ε). Cdc2 western blot served as a loading control. (JPG) [file pgen.1003936.s021.jpg]
