# Supplementary material for: Fission Yeast Shelterin Regulates DNA Polymerases and Rad3ATR Kinase to Limit Telomere Extension
Source: PLoS Genet. 2013 Nov 7;9(11):e1003936. doi: 10.1371/journal.pgen.1003936 (PMC3820796; doi:10.1371/journal.pgen.1003936)
Supplement: Table S1 — Telomere length correction factors (telomere/rDNA) for dot blot-based ChIP. (PDF) [file pgen.1003936.s022.pdf]

Supplementary Table S1 Telomere length correction factors (telomere/rDNA) for dot blot-based ChIP

Tagged protein	Genetic background	Correction factor[a]	Tagged protein	Genetic background	Correction factor[a]
Trt1-myc	wt	1.000±0.011 (n=23)	Rad11-FLAG	wt	1.000±0.026 (n=17)
	poz1Δ	7.523±0.235 (n=22)		*poz1Δ*	2.950±0.063 (n=17)
	rap1Δ	7.576±0.133 (n=23)		*rap1Δ*	6.281±0.152 (n=17)
	taz1Δ	6.507±0.085 (n=22)		*taz1Δ*	4.407±0.093 (n=15)
	trt1-D743A	1.277±0.158 (n=9)			
	trt1-D743A rap1Δ	2.409±0.190 (n=7)			
Pol1-FLAG	wt	1.000±0.013 (n=18)	Tpz1-myc	wt	1.000±0.014 (n=18)
	poz1Δ	2.474±0.044 (n=18)		*poz1Δ*	5.671±0.107 (n=18)
	rap1Δ	1.802±0.032 (n=18)		*rap1Δ*	6.229±0.154 (n=18)
	taz1Δ	1.001±0.027 (n=18)		*taz1Δ*	5.420±0.194 (n=18)
	trt1Δ	0.669±0.035 (n=9)			
	trt1-D743A	0.508±0.049 (n=9)			
	rap1Δ trt1Δ	0.146±0.009 (n=9)			
Pol2-FLAG	wt	1.000±0.020 (n=18)	Ccq1-myc	wt	1.000±0.020 (n=36)
	poz1Δ	5.389±0.146 (n=18)		*poz1Δ*	3.544±0.140 (n=33)
	rap1Δ	6.362±0.151 (n=15)		*rap1Δ*	5.061±0.216 (n=35)
	taz1Δ	5.008±0.087 (n=15)		*taz1Δ*	4.975±0.258 (n=35)
	trt1Δ	0.632±0.015 (n=9)			
	trt1-D743A	0.450±0.014 (n=9)			
	rap1Δ trt1Δ	0.832±0.016 (n=9)			
myc-Rad3	wt	1.000±0.019 (n=9)	Poz1-myc	wt	1.000±0.025 (n=26)
	poz1Δ	3.730±0.097 (n=9)		*rap1Δ*	4.773±0.311 (n=24)
	rap1Δ	4.790±0.114 (n=9)		*taz1Δ*	3.892±0.282 (n=27)
	taz1Δ	2.750±0.101 (n=9)			
myc-Rad26	wt	1.000±0.017 (n=18)	Stn1-myc	wt	1.000±0.021 (n=25)
	poz1Δ	6.842±0.132 (n=18)		*poz1Δ*	3.624±0.237 (n=27)
	rap1Δ	9.516±0.165 (n=18)		*rap1Δ*	5.416±0.364 (n=27)
	taz1Δ	6.105±0.124 (n=16)		*taz1Δ*	4.497±0.260 (n=26)
				rap1Δ trt1Δ	0.666±0.013 (n=9)

[a]Mean ± standard error of the mean. Values are normalized to wt cells with indicated tagged proteins. Number of samples used to determine correction factors are also indicated as (n=#).
