# Supplementary material for: Fission Yeast Shelterin Regulates DNA Polymerases and Rad3ATR Kinase to Limit Telomere Extension
Source: PLoS Genet. 2013 Nov 7;9(11):e1003936. doi: 10.1371/journal.pgen.1003936 (PMC3820796; doi:10.1371/journal.pgen.1003936)
Supplement: Table S2 — Fission yeast strains used in this study. (PDF) [file pgen.1003936.s023.pdf]

Supplementary Table S2 Fission yeast strains used in this study

Figure		Strain	Full Genotype[a]
1B, S1	wt	TN2411	h$^-$ his3-D1
	poz1Δ	YTC8489	h$^-$ ade6-M216 his3-D1 poz1Δ::natMX6
	rap1Δ	YTC9370	h$^+$ his3-D1 rap1Δ::ura4$^+$
	taz1Δ	YTC9369	h$^+$ ade6-M216 his3-D1 taz1Δ::ura4$^+$
	poz1Δ rap1Δ	YTC9371	h$^+$ his3-D1 poz1Δ::natMX6 rap1Δ::ura4$^+$
	poz1Δ taz1Δ	YTC9372	h$^+$ ade6-M216 his3-D1 poz1Δ::natMX6 taz1Δ::ura4$^+$
	rap1Δ taz1Δ	YTC9373	h$^+$ ade6-M210 his3-D1 rap1Δ::ura4$^+$ taz1Δ::LEU2
	poz1Δ rap1Δ taz1Δ	YTC9374	h$^+$ his3-D1 poz1Δ::natMX6 rap1Δ::ura4$^+$ taz1Δ::LEU2
1C	rap1$^+$ (no tag)	TN2411	h$^-$ his3-D1
	rap1-myc	YTC9493	h$^+$ ade6-M210 rap1$^+$-7myc::kanMX
	rap1-myc poz1Δ	YTC9929	h$^-$ ade6-M210 his3-D1 rap1$^+$-7myc::kanMX poz1Δ::natMX6
	rap1-myc taz1Δ	YTC9934	h$^+$ ade6-M210 rap1$^+$-7myc::kanMX taz1Δ::ura4$^+$
	rap1-myc poz1Δ taz1Δ	YTC10060	h$^-$ ade6-M210 his3-D1 rap1$^+$-7myc::kanMX poz1Δ::natMX6 taz1Δ::ura4$^+$
1D	trt1$^+$ (no tag)	SS5264	h$^-$ his3-D1 cdc25-22
	trt1-myc	TN7708	h$^-$ his3-D1 trt1$^+$-G$_8$-13myc::kanMX6 cdc25-22
	trt1-myc poz1Δ	YTC8558	h$^-$ his3-D1 trt1$^+$-G$_8$-13myc::kanMX6 poz1Δ::natMX6 cdc25-22
	trt1-myc rap1Δ	YTC8969	h$^-$ his3-D1 trt1$^+$-G$_8$-13myc::kanMX6 rap1Δ::ura4$^+$ cdc25-22
	trt1-myc taz1Δ	TN8601	h$^-$ his3-D1 trt1$^+$-G$_8$-13myc::kanMX6 taz1Δ::ura4$^+$ cdc25-22
	trt1-myc poz1Δ taz1Δ	YTC9404	h$^-$ his3-D1 trt1$^+$-G$_8$-13myc::kanMX6 poz1Δ::natMX6 taz1Δ::ura4$^+$ cdc25-22
	trt1-myc rap1Δ taz1Δ	YTC9411	h$^-$ ade6-M210 his3-D1 trt1$^+$-G$_8$-13myc::kanMX6 rap1Δ::ura4$^+$ taz1Δ::LEU2 cdc25-22
	trt1-myc poz1Δ rap1Δ	YTC9453	h$^-$ his3-D1 trt1$^+$-G$_8$-13myc::kanMX6 poz1Δ::natMX6 rap1Δ::ura4$^+$ cdc25-22
	trt1-myc poz1Δ rap1Δ taz1Δ	YTC9452	h$^-$ his3-D1 trt1$^+$-G$_8$-13myc::kanMX6 poz1Δ::natMX6 rap1Δ::ura4$^+$ taz1Δ::LEU2 cdc25-22
2, 3C, 5,	trt1-myc	TN7708	h$^-$ his3-D1 trt1$^+$-G$_8$-13myc::kanMX6 cdc25-22
7C, S2, S3,	trt1-myc poz1Δ	YTC8558	h$^-$ his3-D1 trt1$^+$-G$_8$-13myc::kanMX6 poz1Δ::natMX6 cdc25-22
S5, S6,	trt1-myc rap1Δ	YTC8969	h$^-$ his3-D1 trt1$^+$-G$_8$-13myc::kanMX6 rap1Δ::ura4$^+$ cdc25-22
S17, S18	trt1-myc taz1Δ	TN8601	h$^-$ his3-D1 trt1$^+$-G$_8$-13myc::kanMX6 taz1Δ::ura4$^+$ cdc25-22
	pol1-FLAG	TN4781	h$^-$ his3-D1 pol1$^+$-5FLAG::kanMX cdc25-22
	pol1-FLAG poz1Δ	YTC8705	h$^-$ his3-D1 pol1$^+$-5FLAG::kanMX poz1Δ::natMX6 cdc25-22
	pol1-FLAG rap1Δ	YTC9589	h$^-$ his3-D1 pol1$^+$-5FLAG::kanMX rap1Δ::ura4$^+$ cdc25-22
	pol1-FLAG taz1Δ	YTC9375	h$^-$ his3-D1 pol1$^+$-5FLAG::kanMX taz1Δ::ura4$^+$ cdc25-22
	pol2-FLAG	TN4782	h$^+$ ade6-M210 his3-D1 pol2$^+$-5FLAG::kanMX cdc25-22
	pol2-FLAG poz1Δ	YTC8693	h$^-$ ade6-M210 his3-D1 pol2$^+$-5FLAG::kanMX poz1Δ::natMX6 cdc25-22
	pol2-FLAG rap1Δ	YTC9423	h$^-$ ade6-M210 his3-D1 pol2$^+$-5FLAG::kanMX rap1Δ::ura4$^+$ cdc25-22
	pol2-FLAG taz1Δ	YTC9280	h$^-$ ade6-M210 his3-D1 pol2$^+$-5FLAG::kanMX taz1Δ::ura4$^+$ cdc25-22
	wt (no tag)	SS5264	h$^-$ his3-D1 cdc25-22
	poz1Δ (no tag)	YTC8929	h$^-$ his3-D1 poz1Δ::natMX6 cdc25-22
	rap1Δ (no tag)	YTC8938	h$^+$ his3-D1 rap1Δ::ura4$^+$ cdc25-22
	taz1Δ (no tag)	YTC8933	h$^-$ his3-D1 taz1Δ::ura4$^+$ cdc25-22
3, S11	myc-rad26	TN7840	h$^-$ his3-D1 9myc-rad26$^+$::hphMX6 cdc25-22
	myc-rad26 poz1Δ	YTC10349	h$^-$ his3-D1 9myc-rad26$^+$::hphMX6 poz1Δ::natMX6 cdc25-22
	myc-rad26 rap1Δ	YTC10338	h$^-$ his3-D1 9myc-rad26$^+$::hphMX6 rap1Δ::ura4$^+$ cdc25-22
	myc-rad26 taz1Δ	YTC10420	h$^+$ his3-D1 9myc-rad26$^+$::hphMX6 taz1Δ::ura4$^+$ cdc25-22
	rad11-FLAG	BAM5875	h$^+$ his3-D1 rad11$^+$-5FLAG::kanMX cdc25-22
	rad11-FLAG poz1Δ	YTC10334	h$^+$ his3-D1 rad11$^+$-5FLAG::kanMX poz1Δ::natMX6 cdc25-22
	rad11-FLAG rap1Δ	YTC10341	h$^+$ his3-D1 rad11$^+$-5FLAG::kanMX rap1Δ::ura4$^+$ cdc25-22
	rad11-FLAG taz1Δ	YTC10319	h$^-$ his3-D1 rad11$^+$-5FLAG::kanMX taz1Δ::ura4$^+$ cdc25-22

4A	*ccq1-FLAG*	TN6847	h^- his3-D1 ccq1$^+$-5FLAG::kanMX6 cdc25-22
	ccq1-FLAG rap1Δ	YTC10947	h^- ccq1$^+$-5FLAG::kanMX6 rap1Δ::ura4$^+$ cdc25-22
	ccq1-FLAG taz1Δ	YTC10950	h^+ his3-D1 ccq1$^+$-5FLAG::kanMX6 taz1Δ::ura4$^+$ cdc25-22
4B, 5, S12-S15, S17, S18	*ccq1-myc*	TN7456	h^- his3-D1 ccq1$^+$-13myc::kanMX6 cdc25-22
	ccq1-myc poz1Δ	YTC8709	h^- his3-D1 ccq1$^+$-13myc::kanMX6 poz1Δ::natMX6 cdc25-22
	ccq1-myc rap1Δ	YTC9384	h^+ his3-D1 ccq1$^+$-13myc::kanMX6 rap1Δ::ura4$^+$ cdc25-22
	ccq1-myc taz1Δ	YTC9292	h^- his3-D1 ccq1$^+$-13myc::kanMX6 taz1Δ::ura4$^+$ cdc25-22
	tpz1-myc	TN7467	h^- his3-D1 tpz1$^+$-13myc::kanMX6 cdc25-22
	tpz1-myc poz1Δ	YTC9362	h^- his3-D1 tpz1$^+$-13myc::kanMX6 poz1Δ::natMX6 cdc25-22
	tpz1-myc rap1Δ	YTC9307	h^- his3-D1 tpz1$^+$-13myc::kanMX6 rap1Δ::ura4$^+$ cdc25-22
	tpz1-myc taz1Δ	YTC9327	h^- his3-D1 tpz1$^+$-13myc::kanMX6 taz1Δ::ura4$^+$ cdc25-22
	poz1-myc	TN6843	h^- his3-D1 poz1$^+$-13myc::kanMX6 cdc25-22
	poz1-myc rap1Δ	YTC9314	h^- his3-D1 poz1$^+$-13myc::kanMX6 rap1Δ::ura4$^+$ cdc25-22
	poz1-myc taz1Δ	YTC9285	h^- his3-D1 poz1$^+$-13myc::kanMX6 taz1Δ::ura4$^+$ cdc25-22
	stn1-myc	TN6886	h^- his3-D1 stn1$^+$-13myc::kanMX6 cdc25-22
	stn1-myc poz1Δ	YTC8717	h^- his3-D1 stn1$^+$-13myc::kanMX6 poz1Δ::natMX6 cdc25-22
	stn1-myc rap1Δ	YTC9299	h^- his3-D1 stn1$^+$-13myc::kanMX6 rap1Δ::ura4$^+$ cdc25-22
	stn1-myc taz1Δ	YTC9414	h^+ his3-D1 stn1$^+$-13myc::kanMX6 taz1Δ::ura4$^+$ cdc25-22
6A, S19A	*rad26*$^+$ (no tag)	TN2411	h^- his3-D1
	myc-rad26	LS7680	h^- his3-D1 9myc-rad26$^+$::hphMX6
	myc-rad26 trt1Δ	YTC12785	h^- ade6-M216 his3-D1 9myc-rad26$^+$::hphMX6 trt1Δ::his3$^+$
6B-C, 7C, S19	*trt1*$^+$ (no tag)	SS5264	h^- his3-D1 cdc25-22
	trt1-myc	TN7708	h^- his3-D1 trt1$^+$-G$_8$-13myc::kanMX6 cdc25-22
	trt1-myc rap1Δ	YTC8969	h^- his3-D1 trt1$^+$-G$_8$-13myc::kanMX6 rap1Δ::ura4$^+$ cdc25-22
	trt1-D743A-myc	YTC12787	h^- ade6-M210 his3-D1 trt1-D743A-G$_8$-13myc::kanMX6 cdc25-22
	trt1-D743A-myc rap1Δ	YTC12788	h^- ade6-M210 his3-D1 trt1-D743A-G$_8$-13myc::kanMX6 rap1Δ::ura4$^+$ cdc25-22
7, S20	*pol1-FLAG trt1*$^+$	TN4781	h^- his3-D1 pol1$^+$-5FLAG::kanMX cdc25-22
	pol1-FLAG trt1Δ	TN8388[b]	h^- ade6-M210 his3-D1 pol1$^+$-5FLAG::kanMX trt1Δ::his3$^+$ cdc25-22 //pNR210-trt1$^+$(ade6$^+$; P$_{adh}$::tk; trt1$^+$)
	pol1-FLAG trt1-D743A	YTC10066[b]	h^- ade6-M210 his3-D1 pol1$^+$-5FLAG::kanMX trt1-D743A::LEU2 cdc25-22 //pNR210-trt1$^+$ (ade6$^+$; P$_{adh}$::tk; trt1$^+$)
	pol2-FLAG trt1$^+$	TN4782	h^+ ade6-M210 his3-D1 pol2$^+$-5FLAG::kanMX cdc25-22
	pol2-FLAG trt1Δ	TN8393[b]	h^- ade6-M210 his3-D1 pol2$^+$-5FLAG::kanMX trt1Δ::his3$^+$ cdc25-22 //pNR210-trt1$^+$ (ade6$^+$; P$_{adh}$::tk; trt1$^+$)
	pol2-FLAG trt1-D743A	YTC10064[b]	h^- ade6-M210 his3-D1 pol2$^+$-5FLAG::kanMX trt1-D743A::LEU2 cdc25-22 //pNR210-trt1$^+$ (ade6$^+$; P$_{adh}$::tk; trt1$^+$)
8, S21	*pol1-FLAG*	TN4781	h^- his3-D1 pol1$^+$-5FLAG::kanMX cdc25-22
	pol1-FLAG rap1Δ	YTC9589	h^- his3-D1 pol1$^+$-5FLAG::kanMX rap1Δ::ura4$^+$ cdc25-22
	pol1-FLAG rap1Δ trt1Δ	BAM10118[b]	h^- ade6-M210 his3-D1 pol1$^+$-5FLAG::kanMX rap1Δ::ura4$^+$ trt1Δ::his3$^+$ cdc25-22 //pNR210-trt1$^+$ (ade6$^+$; P$_{adh}$::tk; trt1$^+$)
	pol2-FLAG	TN4782	h^+ ade6-M210 his3-D1 pol2$^+$-5FLAG::kanMX cdc25-22
	pol2-FLAG rap1Δ	YTC9423	h^- ade6-M210 his3-D1 pol2$^+$-5FLAG::kanMX rap1Δ::ura4$^+$ cdc25-22
	pol2-FLAG rap1Δ trt1Δ	BAM10114[b]	h- ade6-M210 his3-D1 pol2$^+$-5FLAG::kanMX rap1Δ::ura4$^+$ trt1Δ::his3$^+$ cdc25-22 //pNR210-trt1$^+$ (ade6$^+$; P$_{adh}$::tk; trt1$^+$)
	stn1-myc	TN6886	h^- his3-D1 stn1$^+$-13myc::kanMX6 cdc25-22
	stn1-myc rap1Δ	YTC9299	h^- his3-D1 stn1$^+$-13myc::kanMX6 rap1Δ::ura4$^+$ cdc25-22
	stn1-myc rap1Δ trt1Δ	YTC13543[b]	h- ade6-M210 his3-D1 stn1$^+$-13myc::kanMX6 rap1Δ::ura4$^+$ trt1Δ::his3$^+$ cdc25-22 //pNR210-trt1$^+$ (ade6$^+$; P$_{adh}$::tk; trt1$^+$)
	wt (no tag)	SS5264	h^- his3-D1 cdc25-22

S4	wt	TN4777	*h^- leu1-32::[hENT1 leu1$^+$] his3-D1 his7-366::[hsv-tk his7+] cdc25-22*
	poz1Δ	YTC9777	*h^- leu1-32::[hENT1 leu1$^+$] his3-D1 his7-366::[hsv-tk his7$^+$] poz1Δ::natMX6 cdc25-22*
	rap1Δ	YTC6430	*h^- leu1-32::[hENT1 leu1$^+$] his3-D1 his7-366::[hsv-tk his7$^+$] rap1Δ::ura4$^+$ cdc25-22*
	taz1Δ	YTC6479	*h^- leu1-32::[hENT1 leu1$^+$] his3-D1 his7-366::[hsv-tk his7$^+$] taz1Δ::ura4$^+$ cdc25-22*
S7-S9	*trt1-myc*	TN7708	*h^- his3-D1 trt1$^+$-G_8-13myc::kanMX6 cdc25-22*
	trt1-myc poz1Δ	YTC8558	*h^- his3-D1 trt1$^+$-G_8-13myc::kanMX6 poz1Δ::natMX6 cdc25-22*
	trt1-myc rap1Δ	YTC8969	*h^- his3-D1 trt1$^+$-G_8-13myc::kanMX6 rap1Δ::ura4$^+$ cdc25-22*
	trt1-myc taz1Δ	TN8601	*h^- his3-D1 trt1$^+$-G_8-13myc::kanMX6 taz1Δ::ura4$^+$ cdc25-22*
	myc-rad26	TN7840	*h^- his3-D1 9myc-rad26$^+$::hphMX6 cdc25-22*
	myc-rad26 poz1Δ	YTC10349	*h^- his3-D1 9myc-rad26$^+$::hphMX6 poz1Δ::natMX6 cdc25-22*
	myc-rad26 rap1Δ	YTC10338	*h^- his3-D1 9myc-rad26$^+$::hphMX6 rap1Δ::ura4$^+$ cdc25-22*
	myc-rad26 taz1Δ	YTC10420	*h^+ his3-D1 9myc-rad26$^+$::hphMX6 taz1Δ::ura4$^+$ cdc25-22*
	myc-rad3	YTC13163	*h^+ his3-D1 9myc-rad3$^+$ cdc25-22*
	myc-rad3 poz1Δ	YTC13047	*h^+ his3-D1 9myc-rad3$^+$ poz1Δ::natMX6 cdc25-22*
	myc-rad3 rap1Δ	YTC13053	*h^+ his3-D1 9myc-rad3$^+$ rap1Δ::ura4$^+$ cdc25-22*
	myc-rad3 taz1Δ	YTC13057	*h^+ his3-D1 9myc-rad3$^+$ taz1Δ::ura4$^+$ cdc25-22*
	rad11-FLAG	BAM5875	*h^+ his3-D1 rad11$^+$-5FLAG::kanMX cdc25-22*
	rad11-FLAG poz1Δ	YTC10334	*h^+ his3-D1 rad11$^+$-5FLAG::kanMX poz1Δ::natMX6 cdc25-22*
	rad11-FLAG rap1Δ	YTC10341	*h^+ his3-D1 rad11$^+$-5FLAG::kanMX rap1Δ::ura4$^+$ cdc25-22*
	rad11-FLAG taz1Δ	YTC10319	*h^- his3-D1 rad11$^+$-5FLAG::kanMX taz1Δ::ura4$^+$ cdc25-22*
	tpz1-myc	TN7467	*h^- his3-D1 tpz1$^+$-13myc::kanMX6 cdc25-22*
	tpz1-myc poz1Δ	YTC9362	*h^- his3-D1 tpz1$^+$-13myc::kanMX6 poz1Δ::natMX6 cdc25-22*
	tpz1-myc rap1Δ	YTC9307	*h^- his3-D1 tpz1$^+$-13myc::kanMX6 rap1Δ::ura4$^+$ cdc25-22*
	tpz1-myc taz1Δ	YTC9327	*h^- his3-D1 tpz1$^+$-13myc::kanMX6 taz1Δ::ura4$^+$ cdc25-22*
	ccq1-myc	TN7456	*h^- his3-D1 ccq1$^+$-13myc::kanMX6 cdc25-22*
	ccq1-myc poz1Δ	YTC8709	*h^- his3-D1 ccq1$^+$-13myc::kanMX6 poz1Δ::natMX6 cdc25-22*
	ccq1-myc rap1Δ	YTC9384	*h^+ his3-D1 ccq1$^+$-13myc::kanMX6 rap1Δ::ura4$^+$ cdc25-22*
	ccq1-myc taz1Δ	YTC9292	*h^- his3-D1 ccq1$^+$-13myc::kanMX6 taz1Δ::ura4$^+$ cdc25-22*
	poz1-myc	TN6843	*h^- his3-D1 poz1$^+$-13myc::kanMX6 cdc25-22*
	poz1-myc rap1Δ	YTC9314	*h^- his3-D1 poz1$^+$-13myc::kanMX6 rap1Δ::ura4$^+$ cdc25-22*
	poz1-myc taz1Δ	YTC9285	*h^- his3-D1 poz1$^+$-13myc::kanMX6 taz1Δ::ura4$^+$ cdc25-22*
	stn1-myc	TN6886	*h^- his3-D1 stn1$^+$-13myc::kanMX6 cdc25-22*
	stn1-myc poz1Δ	YTC8717	*h^- his3-D1 stn1$^+$-13myc::kanMX6 poz1Δ::natMX6 cdc25-22*
	stn1-myc rap1Δ	YTC9299	*h^- his3-D1 stn1$^+$-13myc::kanMX6 rap1Δ::ura4$^+$ cdc25-22*
	stn1-myc taz1Δ	YTC9414	*h^+ his3-D1 stn1$^+$-13myc::kanMX6 taz1Δ::ura4$^+$ cdc25-22*
	wt (no tag)	SS5264	*h^- his3-D1 cdc25-22*
	poz1Δ (no tag)	YTC8929	*h^- his3-D1 poz1Δ::natMX6 cdc25-22*
	rap1Δ (no tag)	YTC8938	*h^+ his3-D1 rap1Δ::ura4$^+$ cdc25-22*
	taz1Δ (no tag)	YTC8933	*h^- his3-D1 taz1Δ::ura4$^+$ cdc25-22*
S10A, C, D	*tel1$^+$* (no tag)	SS5264	*h^- his3-D1 cdc25-22*
	myc-tel1	YTC13049	*h^+ ade6-704 his3-D1 9myc-tel1$^+$ cdc25-22*
	myc-tel1	LS8284	*h^- ade6-704 his3-D1 9myc-tel1$^+$*
	poz1Δ (no tag)	YTC8930	*h^+ his3-D1 poz1Δ::natMX6 cdc25-22*
	myc-tel1 poz1Δ	YTC10940	*h^- ade6-704 his3-D1 9myc-tel1$^+$ poz1Δ::natMX6 cdc25-22*
	rap1Δ (no tag)	YTC8938	*h^+ his3-D1 rap1Δ::ura4$^+$ cdc25-22*
	myc-tel1 rap1Δ	YTC10935	*h^+ ade6-704 his3-D1 9myc-tel1$^+$ rap1Δ::ura4$^+$ cdc25-22*
	taz1Δ (no tag)	YTC8933	*h^- his3-D1 taz1Δ::ura4$^+$ cdc25-22*
	myc-tel1 taz1Δ	YTC10932	*h^+ ade6-704 his3-D1 9myc-tel1$^+$ taz1Δ::ura4$^+$ cdc25-22*
S10B, D	*tel1$^+$* (no tag)	TN2411	*h^- his3-D1*
	myc-tel1	LS8284	*h^- ade6-704 his3-D1 9myc-tel1$^+$*
	rap1Δ (no tag)	TN5346	*h^+ his3-D1 rap1Δ::ura4$^+$*

myc-tel1 rap1Δ	YTC10938	*h*$^{-}$ *his3-D1 9myc-tel1*$^{+}$ *rap1Δ::ura4*$^{+}$
rad3-kdΔ (no tag)	TN1678	*h*$^{-}$ *ade6-M216 his3-D1 rad3-kdΔ::kanMX4*
myc-tel1 rad3-kdΔ	YTC12932	*h*$^{-}$ *ade6-704 his3-D1 9myc-tel1*$^{+}$ *rad3-kdΔ::kanMX4*
rad3-kdΔ rap1Δ (no tag)	YTC13045	*h*$^{+}$ *ade6*$^{-}$ *his3-D1 rad3-kdΔ::kanMX4 rap1Δ::ura4*$^{+}$
myc-tel1 rad3-kdΔ rap1Δ	YTC12931	*h*$^{+}$ *ade6-704 his3-D1 9myc-tel1*$^{+}$ *rad3-kdΔ::kanMX4 rap1Δ::ura4*$^{+}$

[a]All strains are *leu1-32 ura4-D18*, except for strains expressing hENT1, which are *leu1-32::[hENT1 leu1*$^{+}$*] ura4-D18.*

[b]Early generation strains that have just lost *trt1*$^{+}$ plasmid (*pNR210-trt1*$^{+}$) were used to ensure that ChIP assays monitored *trt1Δ* or *trt1-D743A* cells carrying longest telomeres as possible.
