# Supplementary material for: Fission Yeast Shelterin Regulates DNA Polymerases and Rad3ATR Kinase to Limit Telomere Extension
Source: PLoS Genet. 2013 Nov 7;9(11):e1003936. doi: 10.1371/journal.pgen.1003936 (PMC3820796; doi:10.1371/journal.pgen.1003936)
Supplement: Table S3 — DNA primers used in this study. (PDF) [file pgen.1003936.s024.pdf]

Supplementary Table S3 DNA primers used in this study

Primer Name	Primer Sequence (5' to 3')	Description
jk380	TATTTCTTTATTCAACTTACCGCACTTC	Used as real-time PCR primers for telomere ChIP [63].
jk381	CAGTAGTGCAGTGTATTATGATAATTAAAATGG	
ars2004-66-F	CGGATCCGTAATCCCAACAA	Used as real-time PCR primers for *ars2004* ChIP [64].
ars2004-66-R	TTTGCTTACATTTTCGGGAACTTA	
BAM140	TTTTCAGGGTCGGTAGAGTCAGAG	Used to PCR amplify a region near *ars3001* within rDNA repeats. The PCR product was used as template to generate rDNA region probe to establish telomere correction factors.
BAM141	CCTCCTTACTTCTCCTTATTCCACG	
trt1-B29	CTTATTCTAAATGAAAGGAGATTAGC	Used to PCR amplify *trt1-G_8-13myc::kanMX6* construct to generate cells carrying *trt1-D743A-G_8-13myc::kanMX6* allele.
trt1-T30	TAGGCTAGGATACTCTATGTGTATGAGAGC	
UraKan-T1	CCCACTGGCTATATGTATGCATTTGTGTTAAAAAAGTTTGTATAGATTATTTAATCTACTCAGCATTCTTTCTCTAA<u>CGCGCCAGATCTGTTTAGCTTGC</u>	Used to swap marker from *ura4*$^+$ to *kanMX4*. 5' end anneals to *ura4*$^+$ and 3' end (underlined) anneals to *kanMX4* module in pFA6a-kanMX4 plasmid.
UraKan-B1	GATATTGACGAAACTTTTTGACATCTAATTTATTCTGTTCCAACACCAATGTTTATAACCAAGTTTTATCTTGTTTG<u>GGCGGCGTTAGTATCGAATCGAC</u>	
