# Supplementary material for: Fission Yeast Shelterin Regulates DNA Polymerases and Rad3ATR Kinase to Limit Telomere Extension
Source: PLoS Genet. 2013 Nov 7;9(11):e1003936. doi: 10.1371/journal.pgen.1003936 (PMC3820796; doi:10.1371/journal.pgen.1003936)
Supplement: Table S4 — Plasmids used in this study. (PDF) [file pgen.1003936.s025.pdf]

Supplementary Table S4 Plasmids used in this study

Plasmid (Lab stock #)	Genes	Description
pTELO (254)	Fission yeast telomere fragment; *ampR*	Carries a telomeric repeat fragment (ApaI-SacI) used in generating a telomere probe for Southern blot analysis [54].
pNR210-trt1$^+$ (290)	*ade6*$^+$; *trt1*$^+$; P_{adh}*::tk*; *ampR*	Trt1 plasmid used to maintain telomeres. It also expresses *tk* (herpes simplex virus thymidine kinase) gene to allow counter selection of the plasmid on media containing 5FdUR [65].
pFA6a-kanMX4 (4)	*kanMX4*; *ampR*	Used as a PCR template for *kanMX4* [55] to swap marker to generate *rad3-kdΔ::kanMX4* strain.
pTM580 (461)	Full length *GAD-tpz1*; *LEU2*; *ampR*	pGAD-GH-Tpz1 full length plasmid from Ishikawa lab [6].
pGAD-GH-tpz1$_{2\text{-}420}$ (838)	*GAD-tpz1*$_{2\text{-}420}$; *LEU2*; *ampR*	Expresses truncated GAL4 AD-Tpz1 (2-420).
pGAD-GH-tpz1$_{224\text{-}508}$ (814)	*GAD-tpz1*$_{224\text{-}508}$; *LEU2*; *ampR*	Expresses truncated GAL4 AD-Tpz1 (224-508).
pGAD-GH-tpz1$_{224\text{-}420}$ (818)	*GAD-tpz1*$_{224\text{-}420}$; *LEU2*; *ampR*	Expresses truncated GAL4 AD-Tpz1 (224-420).
pGAD-GH-tpz1$_{2\text{-}223}$ (812)	*GAD-tpz1*$_{2\text{-}223}$; *LEU2*; *ampR*	Expresses truncated GAL4 AD-Tpz1 (2-223).
pGAD-GH-tpz1$_{421\text{-}508}$ (816)	*GAD-tpz1*$_{421\text{-}508}$; *LEU2*; *ampR*	Expresses truncated GAL4 AD-Tpz1 (421-508).
pGBKT7-stn1 (476)	*DBD-stn1*; *TRP1*; *kanR*	Expresses GAL4 DNA BD-Stn1.
pGBKT7-ten1 (474)	*DBD-ten1*; *TRP1*; *kanR*	Expresses GAL4 DNA BD-Ten1.
pGBKT7-stn1+ten1 (570)	*DBD-stn1*; *ten1*; *TRP1*; *kanR*	Expresses GAL4 DNA BD-Stn1 and wt Ten1. (Not fused to either GAL4 DNA BD or GAL4 AD.)
