# Supplementary material for: Fission Yeast Shelterin Regulates DNA Polymerases and Rad3ATR Kinase to Limit Telomere Extension
Source: PLoS Genet. 2013 Nov 7;9(11):e1003936. doi: 10.1371/journal.pgen.1003936 (PMC3820796; doi:10.1371/journal.pgen.1003936)
Supplement: Supporting Information S1 — A single PDF file containing all Supporting Information (Figures S1–S21 and Tables S1–S4). (PDF) [file pgen.1003936.s026.pdf]

Fission Yeast Shelterin Regulates DNA Polymerases and Rad3^{ATR} Kinase to Limit Telomere Extension

Ya-Ting Chang[1], Bettina A. Moser[1] and Toru M. Nakamura[1,*]

[1]Department of Biochemistry and Molecular Genetics, College of Medicine, University of Illinois at Chicago, Chicago, IL, USA

Running Title: Cell cycle regulation of telomere maintenance

*E-mail: nakamut@uic.edu

This file contains:

Supplemental Figures S1-S21
Supplemental Tables S1-S4

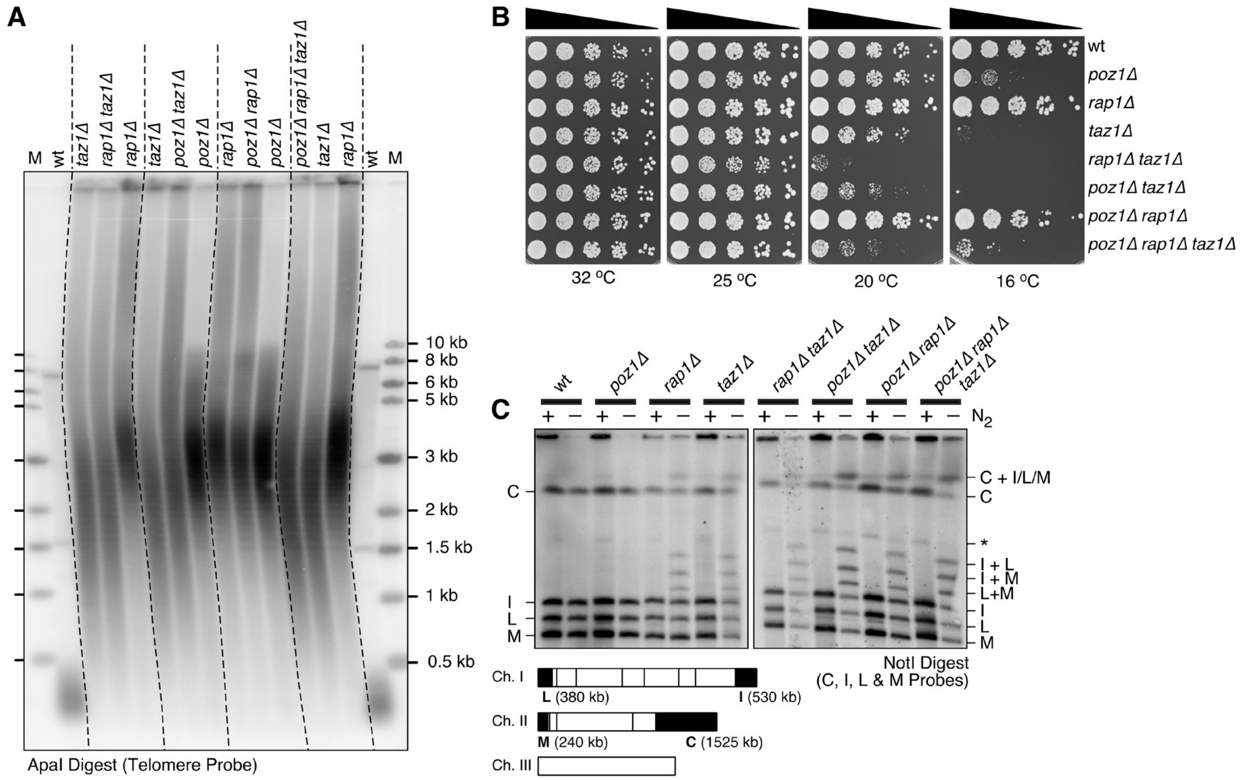

Figure S1 Epistasis analysis of *poz1Δ*, *rap1Δ* and *taz1Δ* cells. (**A**) Telomere length analysis for indicated strains. Genomic DNA was prepared after extensive restreaks on YES plates to ensure telomere length equilibrium. After digestion with ApaI, DNA was fractionated on a 1% agarose gel and processed for Southern blot analysis with a telomere probe. Quantitative analysis of telomere length distribution for this gel is shown in Figure 1B. (**B**) Analysis of cell growth at lower temperatures. Five-fold serial dilution of the indicated strains are plated on YES, and grown at indicated temperatures. (**C**) Chromosome fusion analysis of G_1 arrested cells. Genomic DNA was prepared in agarose plugs from G_1 arrested cells, digested with NotI, fractionated on a 1% agarose gel by pulsed-field gel electrophoresis, and processed for Southern blot analysis with probes specific for C, I, L and M NotI chromosomal fragments. A NotI restriction map of *S. pombe* chromosomes is shown below, with telomeric C, I, L, and M fragments marked as black boxes.

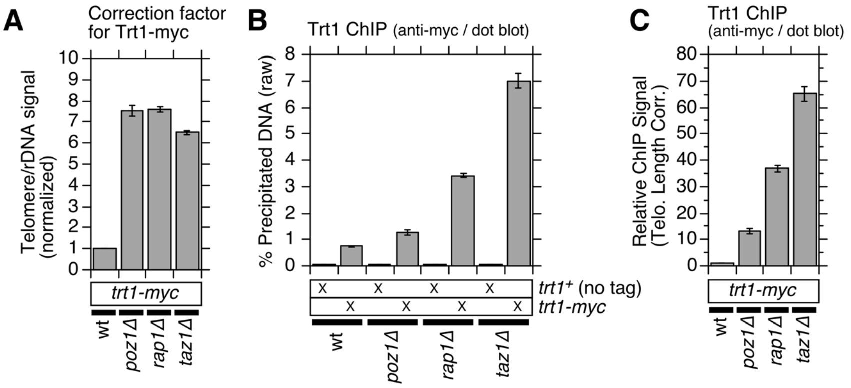

Figure S2 Analysis of Trt1^{TERT} recruitment to telomeres by dot blot-based asynchronous ChIP assays with telomeric DNA probe. (**A**) Telomere correction factors for Trt1-myc strains were established by determining telomere/rDNA hybridization signal ratios relative to wt cells. Telomere correction factors for other epitope tagged strains are shown in Supplementary Table S1. (**B**) Raw % precipitated DNA values for dot blot-based Trt1-myc ChIP assays for the indicated genotypes. (**C**) Telomere length corrected ChIP data for Trt1-myc. (See Materials and Methods section for details.) Error bars correspond to SEM.

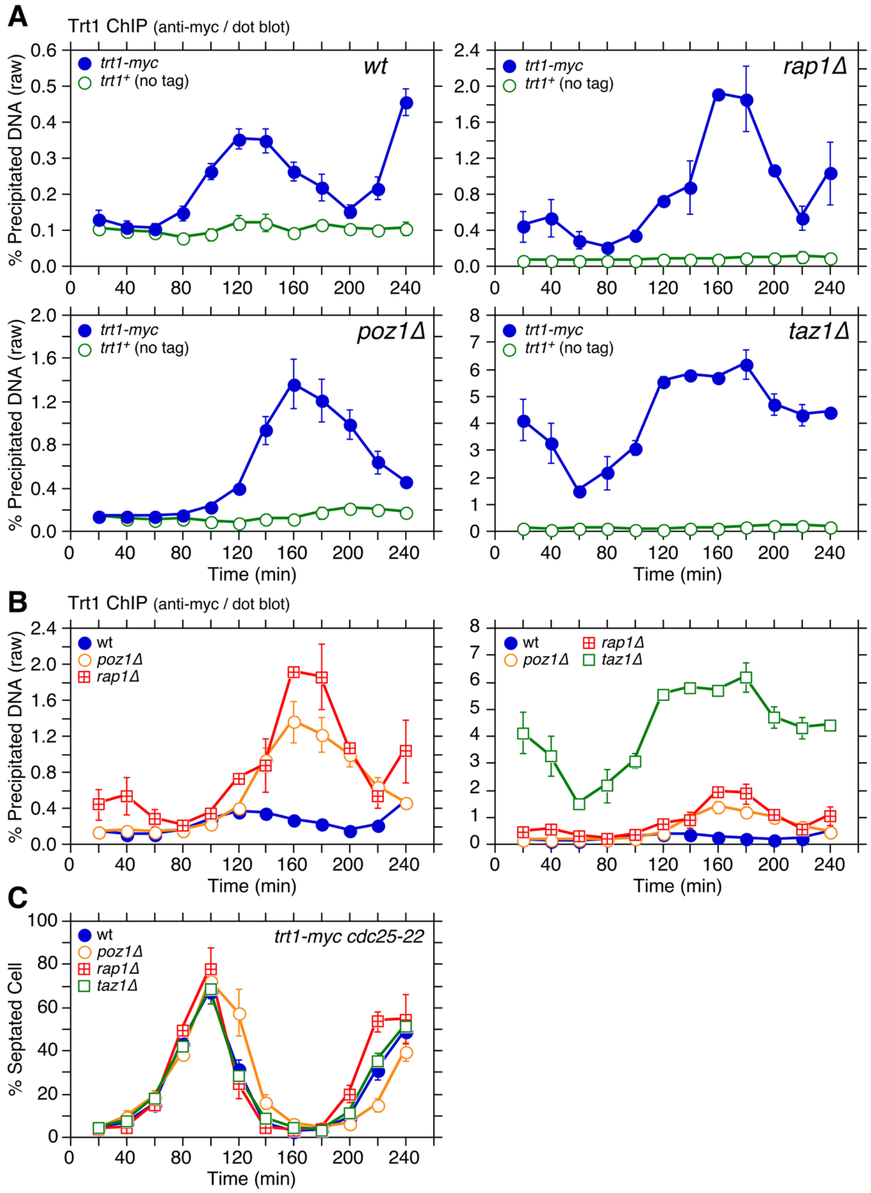

Figure S3 Raw data of dot blot-based cell cycle ChIP assays for Trt1^{TERT}. (**A**, **B**) Cell cycle ChIP assays were performed with *cdc25-22* synchronized cell cultures for wt, *poz1Δ*, *rap1Δ* or *taz1Δ* cells, and % precipitated DNA was determined by hybridization of a telomeric probe to dot blotted input and ChIP samples. (**C**) % septated cells were measured to monitor cell cycle progression of *cdc25-22* synchronized cell cultures for the indicated genotypes. Error bars correspond to SEM.

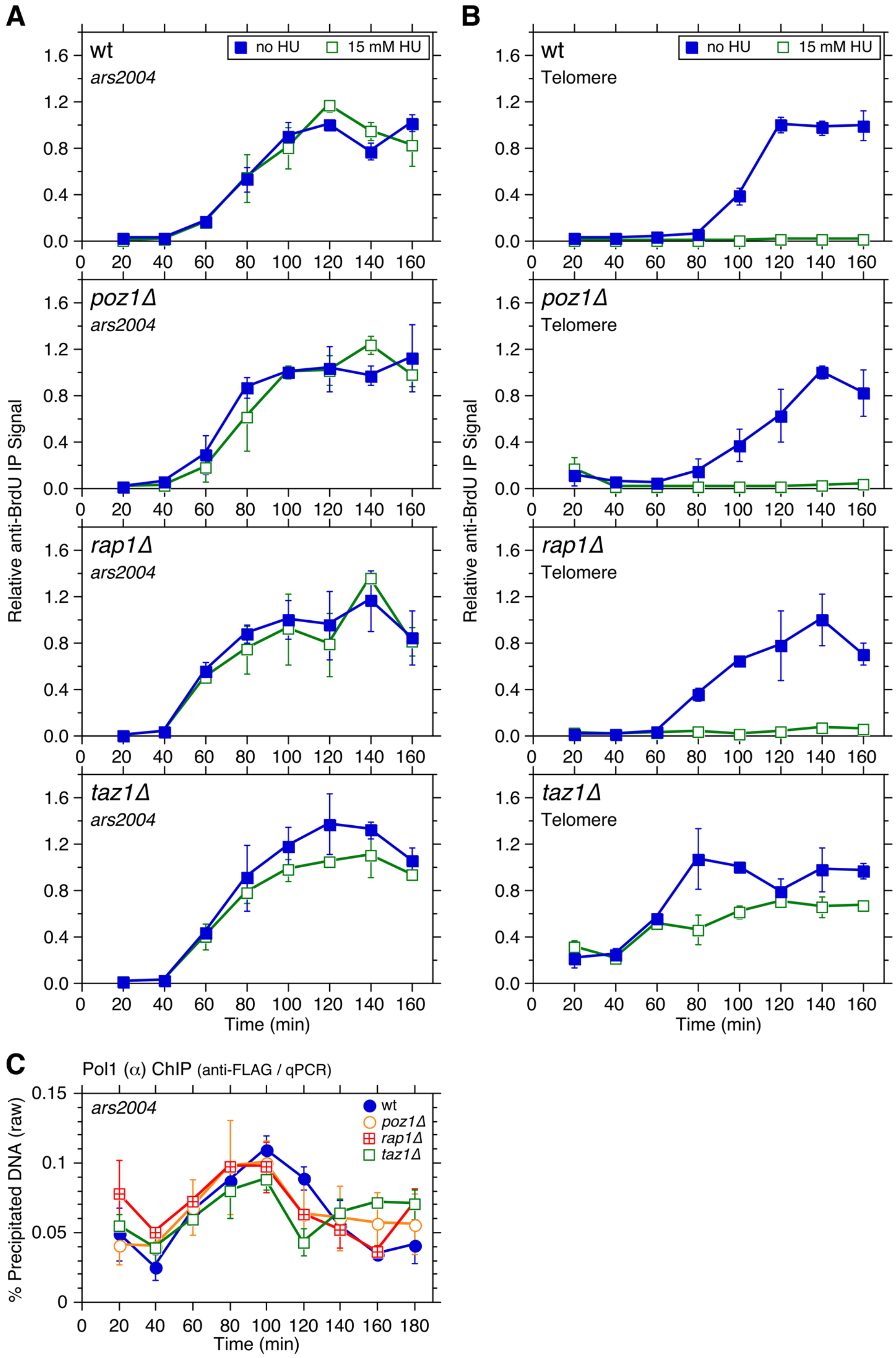

Figure S4 DNA replication timing monitored by incorporation of BrdU in *cdc25-22* synchronized cells for (**A**) *ars2004* and (**B**) telomeres [25]. BrdU incorporation at telomeres is inhibited by addition of 15 mM HU for wt, *poz1Δ* and *rap1Δ* cells but not for *taz1Δ* cells. BrdU is incorporated into *ars2004* with similar kinetics in the presence or absence of HU for all genetic backgrounds tested. (**C**) Pol1 (α) showed similar timing of recruitment to *ars2004* in all genetic backgrounds tested. Error bars correspond to SEM.

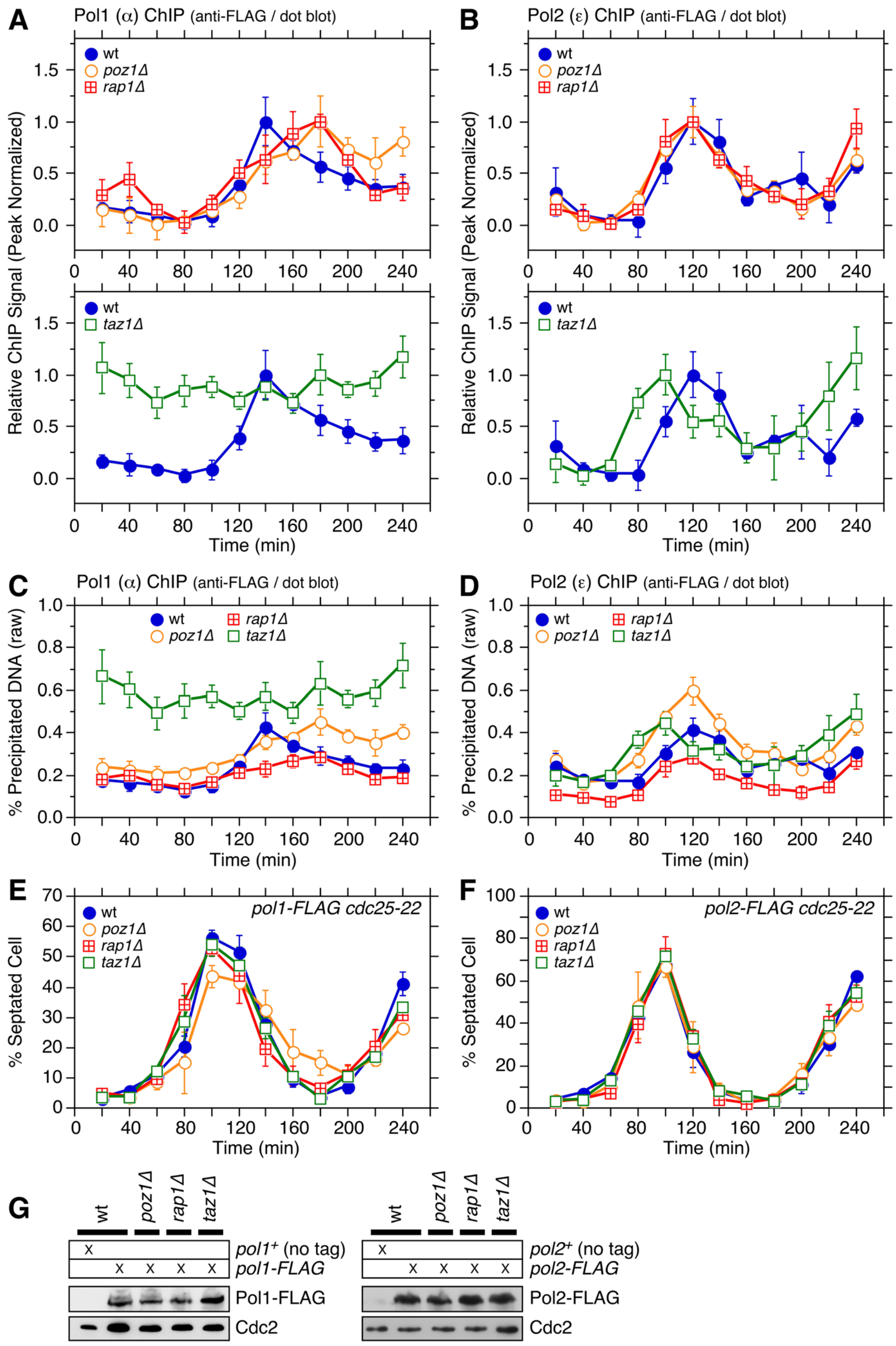

Figure S5 Cell cycle ChIP assays for DNA polymerases. (**A**, **B**) Peak normalized cell cycle ChIP data for Pol1 (α) (A) and Pol2 (ε) (B). For Pol2 (ε), Student's t-test found a statistically significant difference in telomere binding at 80 min (p=0.03) for wt vs. *taz1Δ* cells. (**C**, **D**) Raw data of dot blot-based cell cycle ChIP assays for Pol1 (α) (C) and Pol2 (ε) (D), performed with *cdc25-22* synchronized cell cultures and telomeric DNA probe. (**E**, **F**) % septated cells were measured to monitor cell cycle progression of *cdc25-22* synchronized cell cultures for Pol1 (α) (E) and Pol2 (ε) (F) ChIP assays. Error bars correspond to SEM. (**G**) Anti-FLAG western blot analysis indicated comparable expression levels in different genetic backgrounds for both Pol1 (α) and Pol2 (ε). Cdc2 western blot served as loading control.

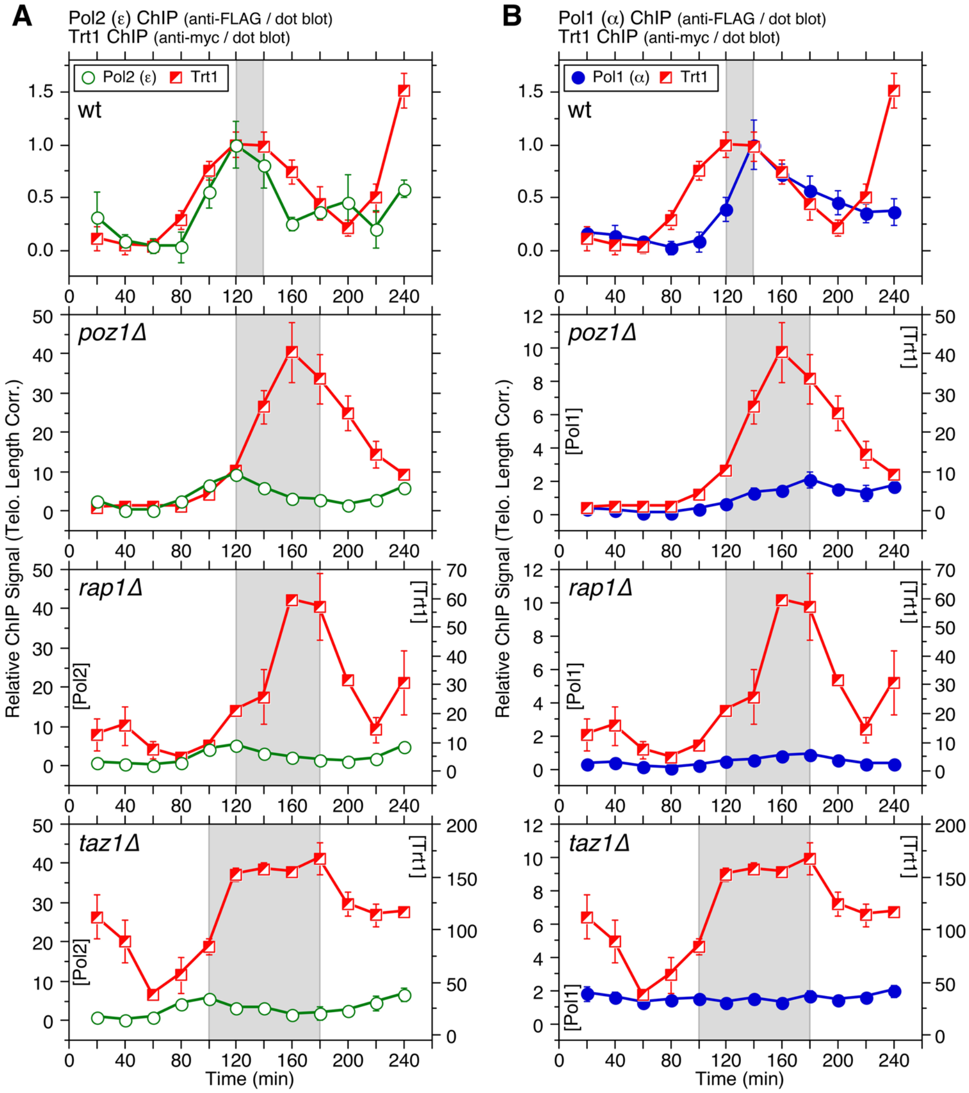

Figure S6 Comparison of cell cycle ChIP data among DNA polymerases and Trt1^{TERT}. Comparison of telomere length corrected ChIP data between Pol2 (ε) and Trt1 (**A**) or Pol1 (α) and Trt1 (**B**) in indicated genomic backgrounds. For explanation of shaded areas in graphs, see Figure 2 legend. Error bars correspond to SEM.

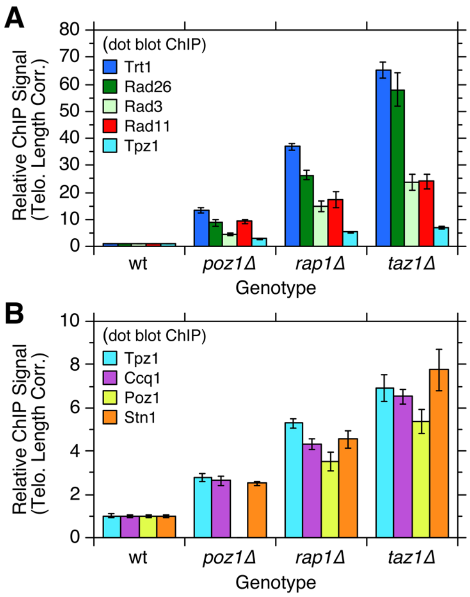

Figure S7 Telomere length corrected dot blot-based asynchronous ChIP data for indicated proteins in wt, *poz1Δ*, *rap1Δ* and *taz1Δ* cells. (**A**) Raw ChIP data from Supplementary Figures S8-S9 for $Trt1^{TERT}$, $Rad26^{ATRIP}$, $Rad3^{ATR}$, $Rad11^{RPA}$ and Tpz1 were corrected for telomere length and normalized to wt cells. Compared to wt cells, *poz1Δ*, *rap1Δ*, and *taz1Δ* cells all showed statistically significant increases in telomere association for $Trt1^{TERT}$ ($p<1.2x10^{-11}$), $Rad26^{ATRIP}$ ($p<6.4x10^{-4}$), $Rad3^{ATR}$ (p<0.047 for *poz1Δ* while $p<1.8x10^{-5}$ for *rap1Δ* and *taz1Δ*), $Rad11^{RPA}$ ($p<1.6x10^{-3}$) and Tpz1 ($p<3.2x10^{-7}$). (**B**) Raw ChIP data from Supplementary Figure S9 for Tpz1, Ccq1, Poz1 and Stn1 were corrected for telomere length and normalized to wt cells. Compared to wt cells, *poz1Δ*, *rap1Δ*, and *taz1Δ* cells all showed statistically significant increases in telomere association for Ccq1 ($p<1.8x10^{-4}$), Poz1 ($p<1.5x10^{-5}$) and Stn1 ($p<1.1x10^{-5}$). Error bars correspond to SEM.

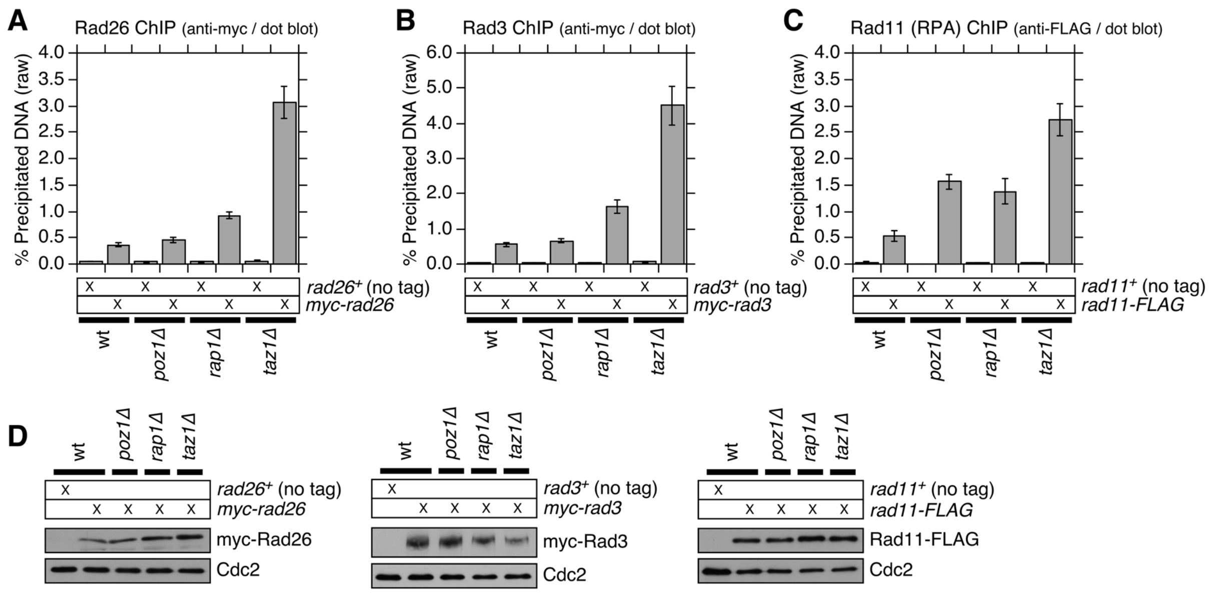

Figure S8 Raw % precipitated DNA against input DNA for Rad26^{ATRIP} (**A**), Rad3^{ATR} (**B**) and Rad11^{RPA} (**C**) obtained by dot blot-based asynchronous ChIP assays with telomeric DNA probe. Error bars correspond to SEM. (**D**) Anti-myc (Rad26 and Rad3) and anti-FLAG (Rad11) western blot analysis indicated comparable expression levels in different genetic backgrounds. Cdc2 western blot served as a loading control.

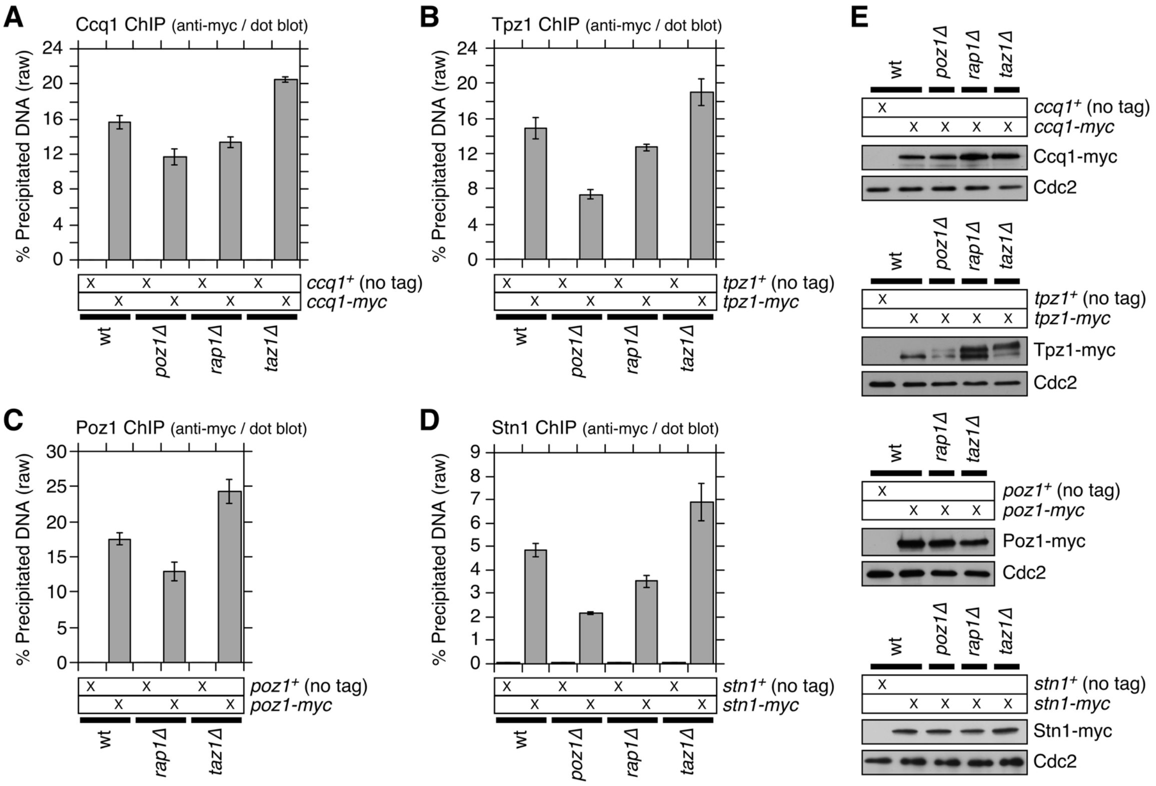

Figure S9 Raw % precipitated DNA against input DNA for Ccq1 (**A**), Tpz1 (**B**), Poz1 (**C**) and Stn1 (**D**) obtained by dot blot-based asynchronous ChIP assays with telomeric DNA probe. Error bars correspond to SEM. (**E**) Anti-myc western blot analyses indicated comparable expression levels for all proteins in different genetic backgrounds. Cdc2 western blot served as a loading control.

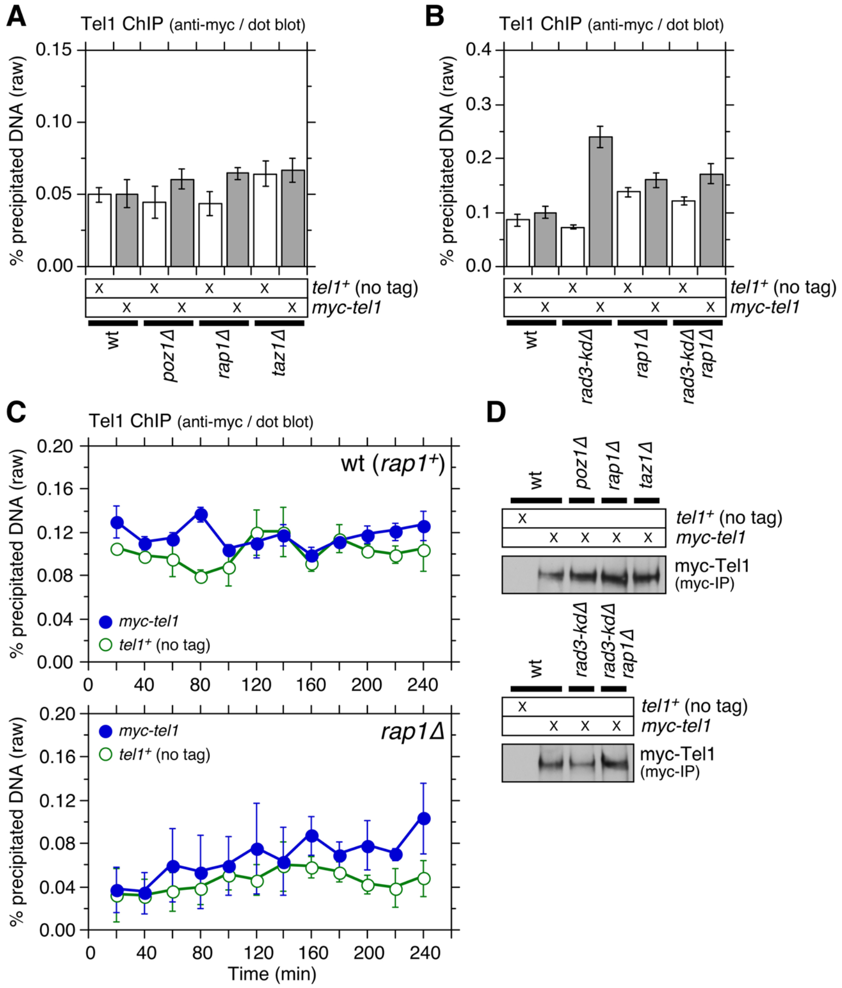

Figure S10 $Tel1^{ATM}$ does not show increased binding to telomeres in *poz1Δ*, *rap1Δ* and *taz1Δ* cells. (**A, B**) Raw % precipitated DNA against input DNA for $Tel1^{ATM}$ obtained by dot blot-based asynchronous ChIP assays with telomeric DNA probe. For (A), none of the strains showed statistically significant $Tel1^{ATM}$ binding over no tag controls. For (B), only *rad3-kdΔ* cells [57] showed statistically significant $Tel1^{ATM}$ binding over no tag control ($p=6.0\times10^{-4}$). (**C**) Raw data of dot blot-based cell cycle ChIP assays for $Tel1^{ATM}$ in wt or *rap1Δ* cells, performed with *cdc25-22* synchronized cell cultures and telomeric DNA probe. Among all time points, only wt cells at 80 min showed statistically significant $Tel1^{ATM}$ binding over no tag control ($p=4.0\times10^{-3}$). Error bars correspond to SEM. (**D**) While myc-Tel1 expressed from its endogenous promoter could not be detected in whole cell extracts, comparable amounts of $Tel1^{ATM}$ were immunoprecipitated (IP) with anti-myc antibody in different genetic backgrounds.

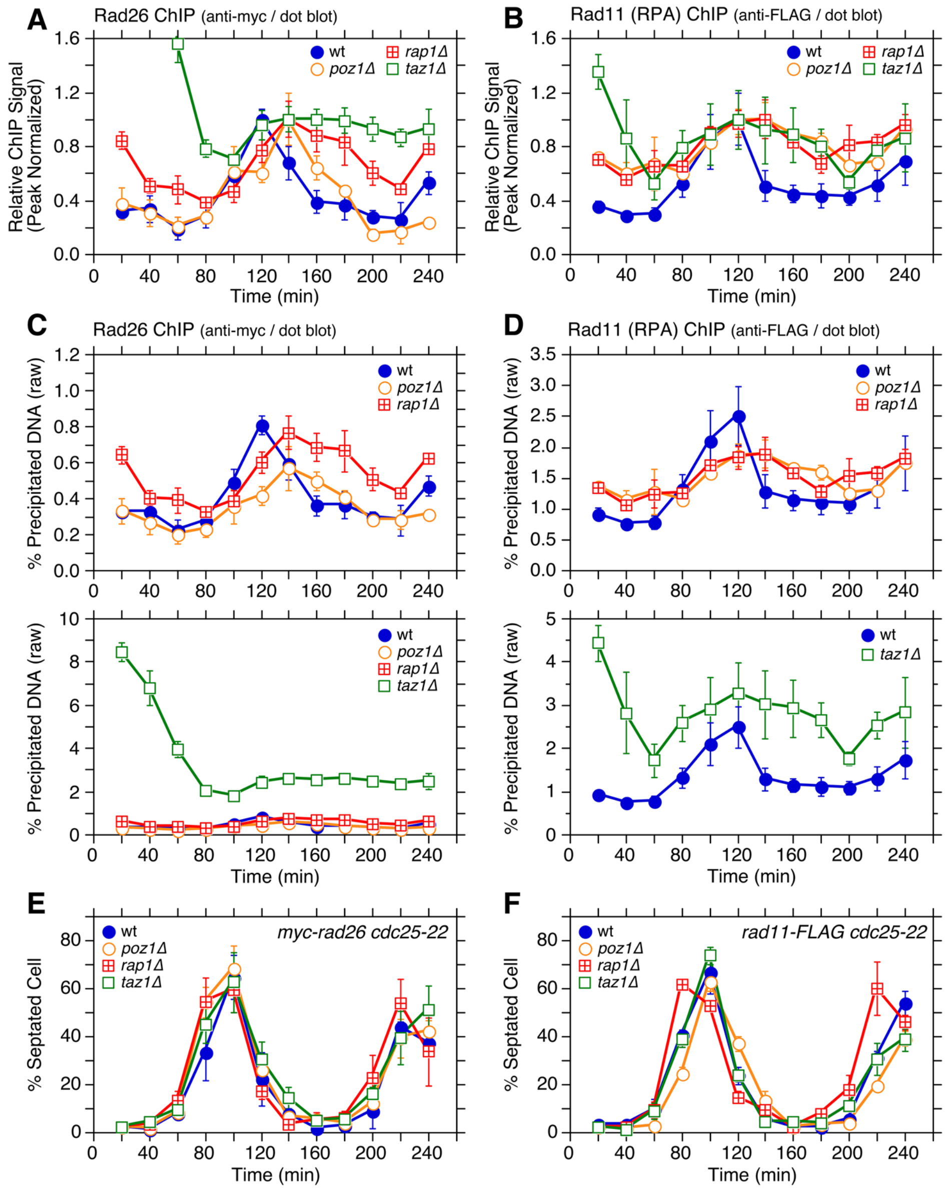

Figure S11 Cell cycle ChIP assays for Rad26^{ATRIP} and Rad11^{RPA}. (**A**, **B**) Peak normalized cell cycle ChIP data for Rad26 (A) and Rad11 (B). (**C**, **D**) Raw data of dot blot-based cell cycle ChIP assays for Rad26 (C) and Rad11 (D), performed with *cdc25-22* synchronized cell cultures and telomeric DNA probe. (**E**, **F**) % septated cells were measured to monitor cell cycle progression of *cdc25-22* synchronized cell cultures for Rad26 (E) and Rad11 (F) ChIP assays. Error bars correspond to SEM.

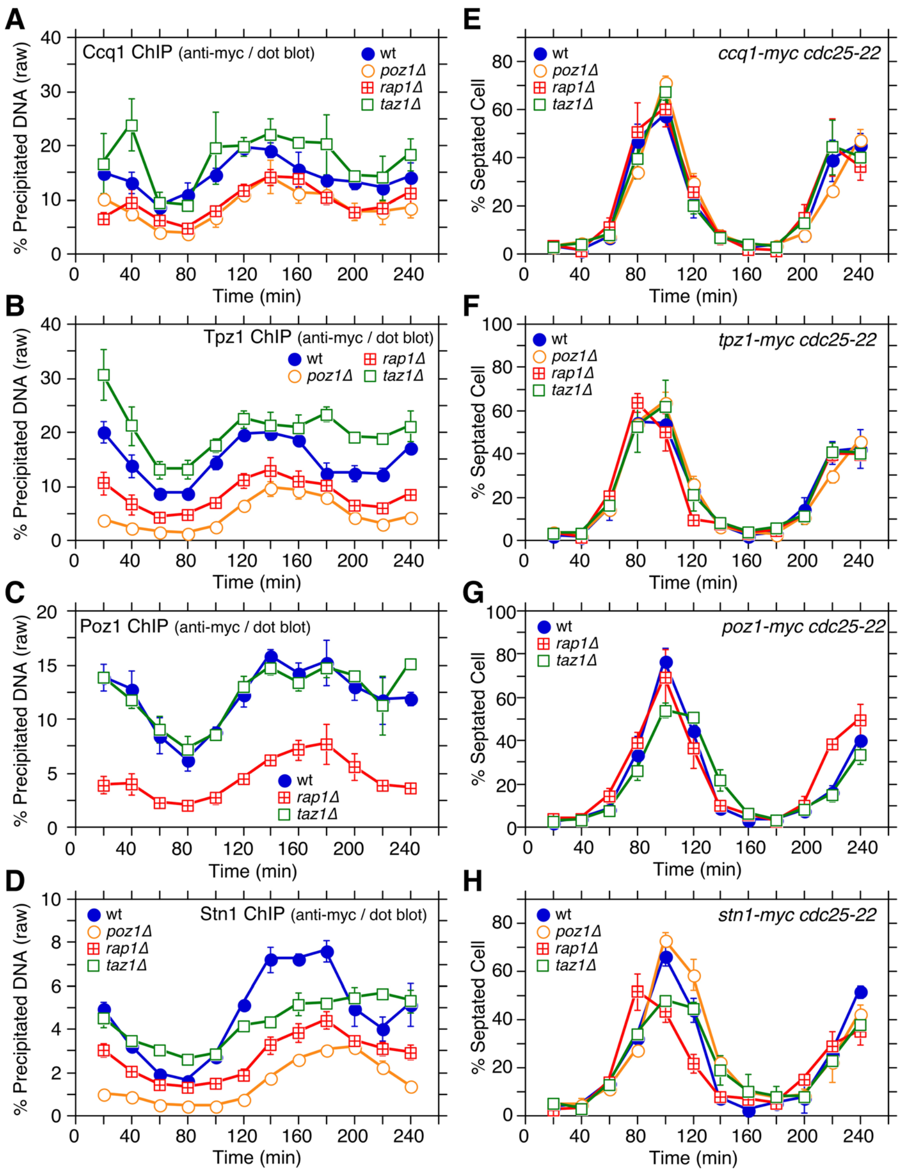

Figure S12 Cell cycle ChIP assays for shelterin subunits and Stn1. (**A-D**) Raw data of dot blot-based cell cycle ChIP assays for Ccq1 (A), Tpz1 (B), Poz1 (C) and Stn1 (D), performed with *cdc25-22* synchronized cell cultures and telomeric DNA probe. (**E-H**) % septated cells were measured to monitor cell cycle progression of *cdc25-22* synchronized cell cultures for Ccq1 (E), Tpz1 (F), Poz1 (G) and Stn1 (H) ChIP assays. Error bars correspond to SEM.

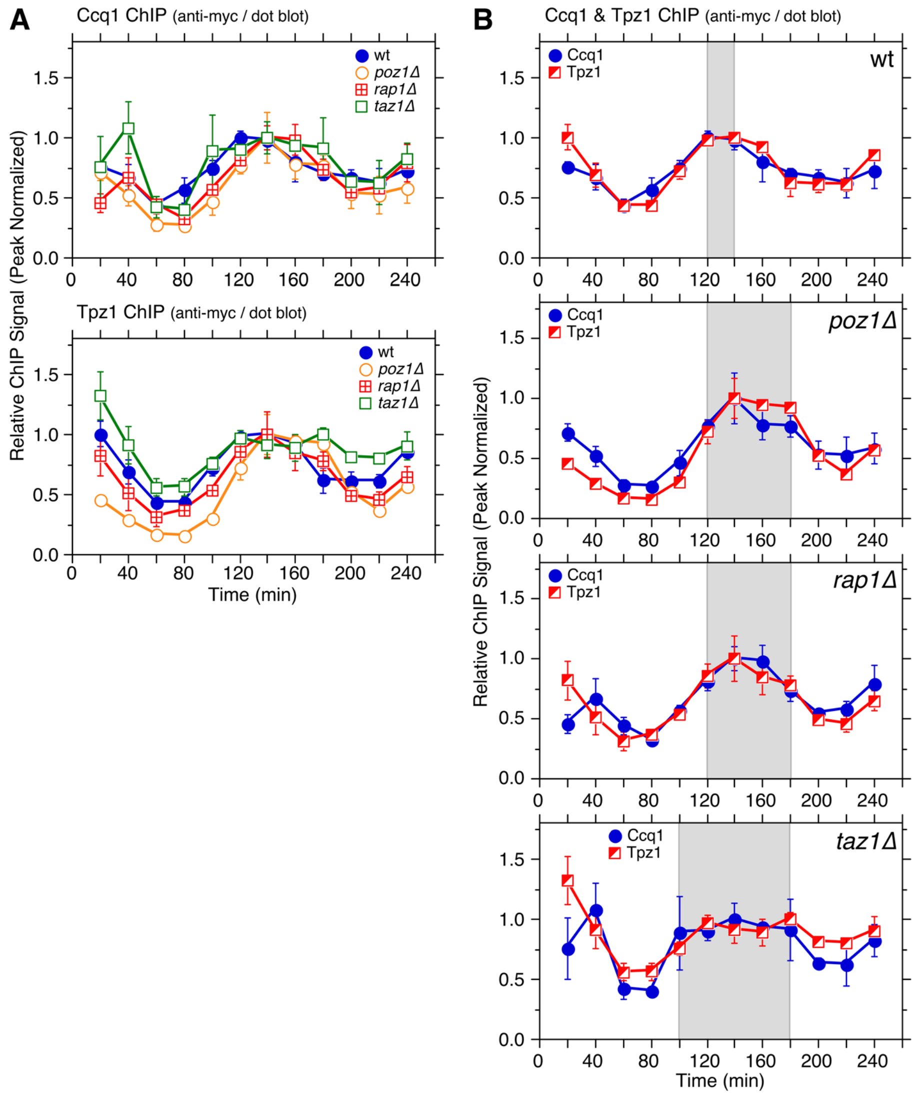

Figure S13 Comparison of peak normalized cell cycle ChIP data between Ccq1 and Tpz1. (**A**) Peak normalized ChIP data for either Ccq1 or Tpz1 in different genetic backgrounds were plotted to compare changes in temporal association with telomeres. (**B**) Comparison of peak normalized ChIP data indicated that temporal changes in telomere association for Ccq1 and Tpz1 are nearly identical in all genetic backgrounds tested. For explanation of shaded areas in graphs, see Figure 2 legend. Error bars correspond to SEM.

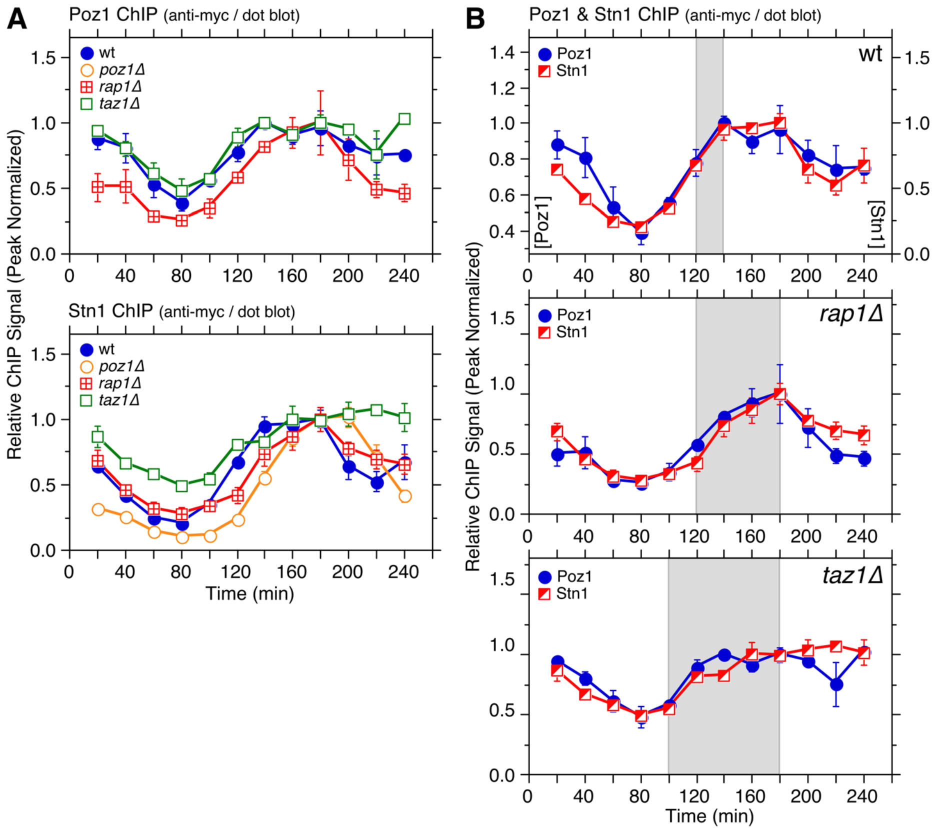

Figure S14 Comparison of peak normalized cell cycle ChIP data between Poz1 and Stn1. (**A**) Peak normalized ChIP data for either Poz1 or Stn1 in different genetic backgrounds were plotted to compare changes in temporal association with telomeres. (**B**) Comparison of peak normalized ChIP data indicated that temporal changes in telomere association for Poz1 and Stn1 are nearly identical in wt, *rap1Δ* and *taz1Δ* cells. For explanation of shaded areas in graphs, see Figure 2 legend. Error bars correspond to SEM.

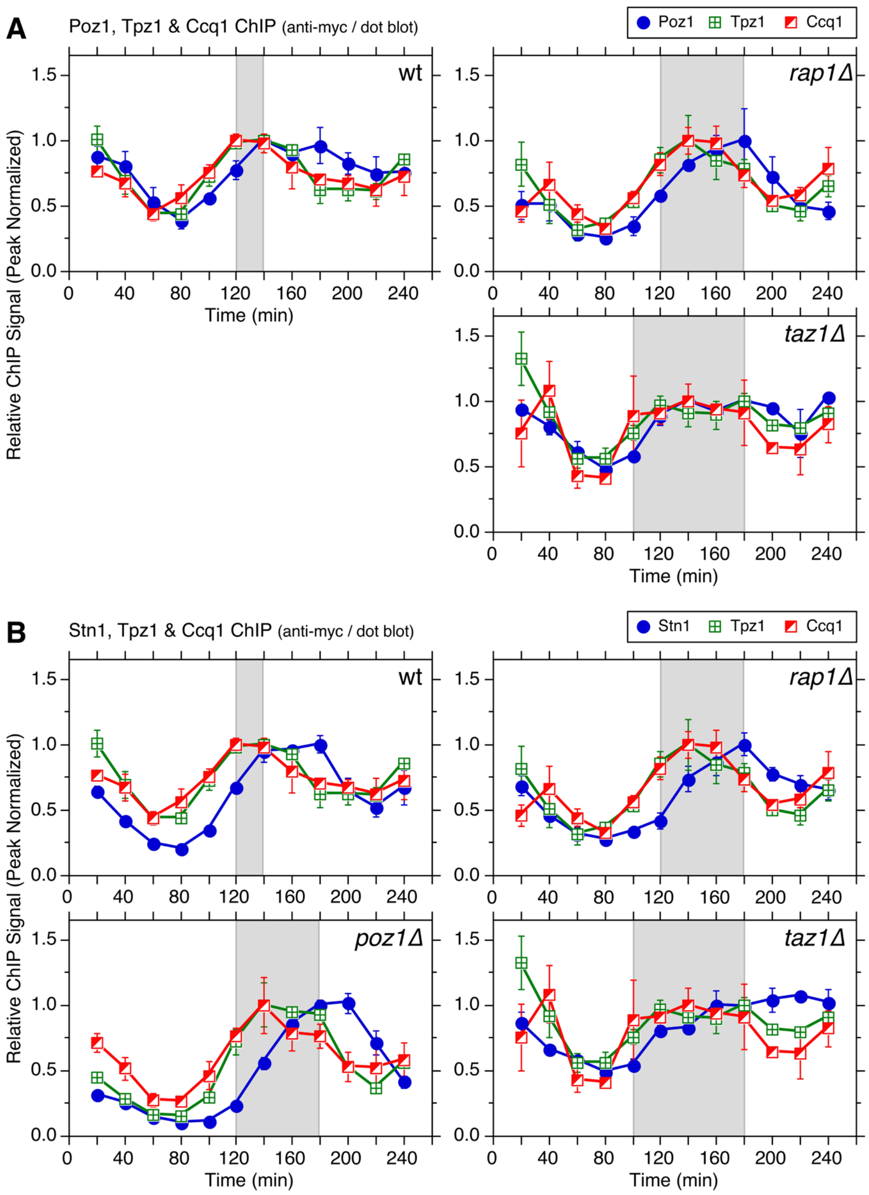

Figure S15 Comparison of cell cycle ChIP data among Ccq1, Tpz1, Poz1 and Stn1. (**A**) Comparison of peak normalized ChIP data for Poz1, Tpz1 and Ccq1 in wt, *rap1Δ* and *taz1Δ* cells. For Tpz1 vs. Poz1, Student's t-test found p=0.053 at 120 min (94.7% confidence level) for wt cells, and p=0.058 at 80 min (94.2% confidence level) and p=0.09 at 100 min (91% confidence level) for *rap1Δ* cells. For Ccq1 vs. Poz1, Student's t-test found p=0.045 at 100 min (95.5% confidence level) and p=0.071 at 120 min (92.9% confidence level) for wt cells, and p=0.082 at 100 min (91.8% confidence level) for *rap1Δ* cells. (**B**) Comparison of peak normalized ChIP data for Stn1, Tpz1 and Ccq1 in wt, *poz1Δ*, *rap1Δ* and *taz1Δ* cells. For Tpz1 vs. Stn1, differences were statistically significant at 60-120 min for wt cells (p<0.03), at 100, 120, 200 and 220 min for *poz1Δ* cells (p<0.04), and at 100, 120, 200 min for *rap1Δ* cells (p<0.01). For Ccq1 vs. Stn1, differences were statistically significant at 100, 120 and 180 min for wt cells (p<0.03), at 80 and 120 min for *poz1Δ* cells (p<0.04), and at 100, 120, 200 min for *rap1Δ* cells (p<0.02). For explanation of shaded areas in graphs, see Figure 2 legend. Error bars correspond to SEM.

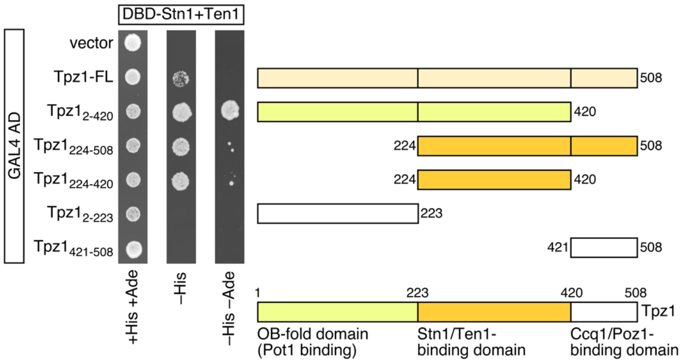

Figure S16 Yeast 3-hybrid assay to monitor interaction between Tpz1 and Stn1-Ten1. Various truncation constructs of Tpz1 were tested for interaction with Stn1 and Ten1. Based on cell growth on –His selection plate, a Tpz1 fragment containing amino acids 224-420 was the smallest Tpz1 construct that retained interaction with Stn1 and Ten1. Based on growth on –His –Ade plate, a Tpz1 fragment containing amino acids 2-420 showed strongest interaction with Stn1 and Ten1.

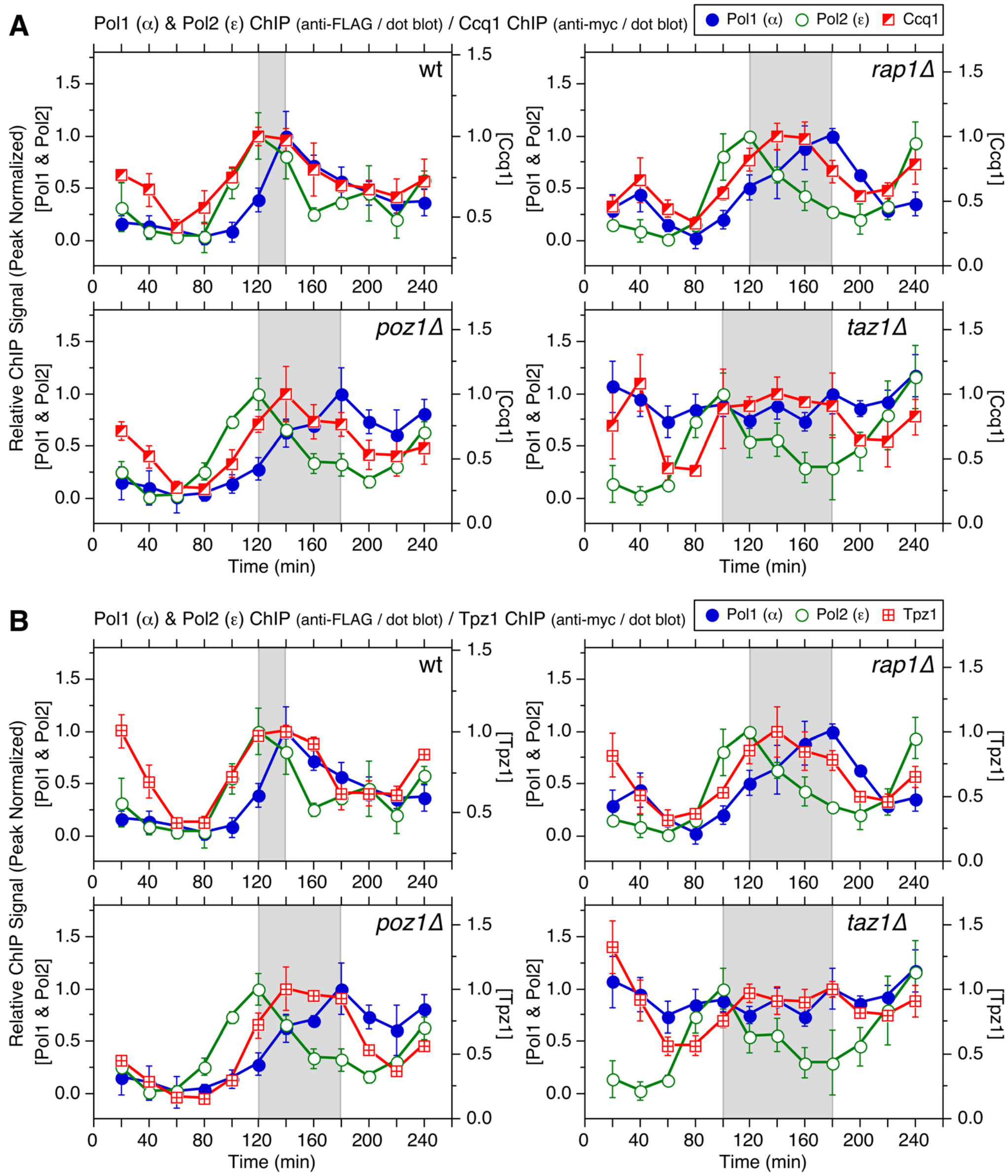

Figure S17 Comparison of cell cycle ChIP data among DNA polymerases, Ccq1 and Tpz1. Comparison of peak normalized ChIP data for Pol1 (α), Pol2 (ε) and Ccq1 (**A**) or Pol1 (α), Pol2 (ε) and Tpz1 (**B**) in wt, *poz1Δ*, *rap1Δ*, and *taz1Δ* cells. For explanation of shaded areas in graphs, see Figure 2 legend. Error bars correspond to SEM.

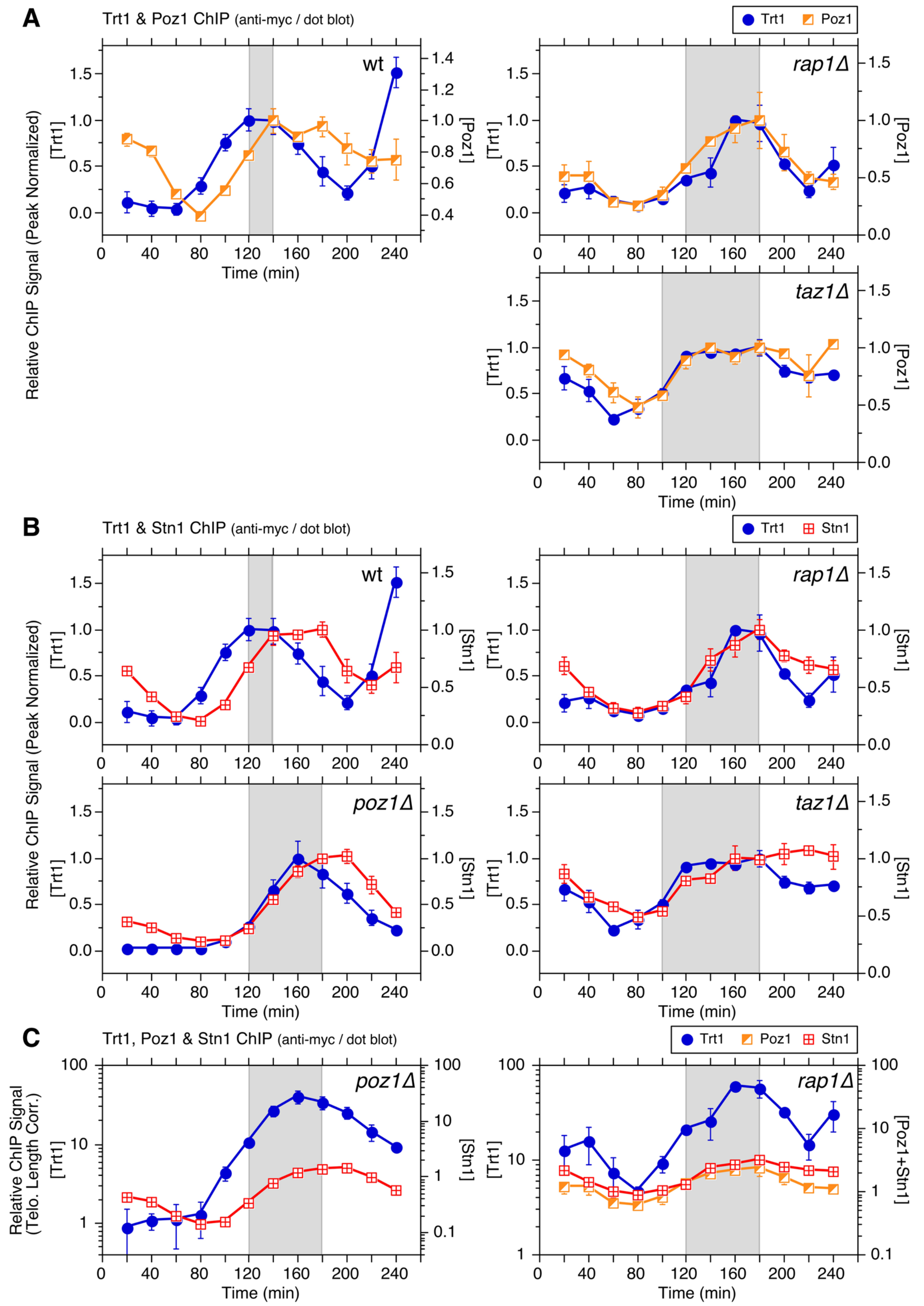

Figure S18 Comparison of cell cycle ChIP data among $Trt1^{TERT}$, Poz1 and Stn1. Comparison of peak normalized ChIP data between Trt1 and Poz1 (**A**) or Trt1 and Stn1 (**B**) for indicated genomic backgrounds. (**C**) Comparison of peak normalized ChIP data among Trt1, Poz1 and Stn1 in *poz1Δ* or *rap1Δ*, plotted on log scale. For explanation of shaded areas in graphs, see Figure 2 legend. Error bars correspond to SEM.

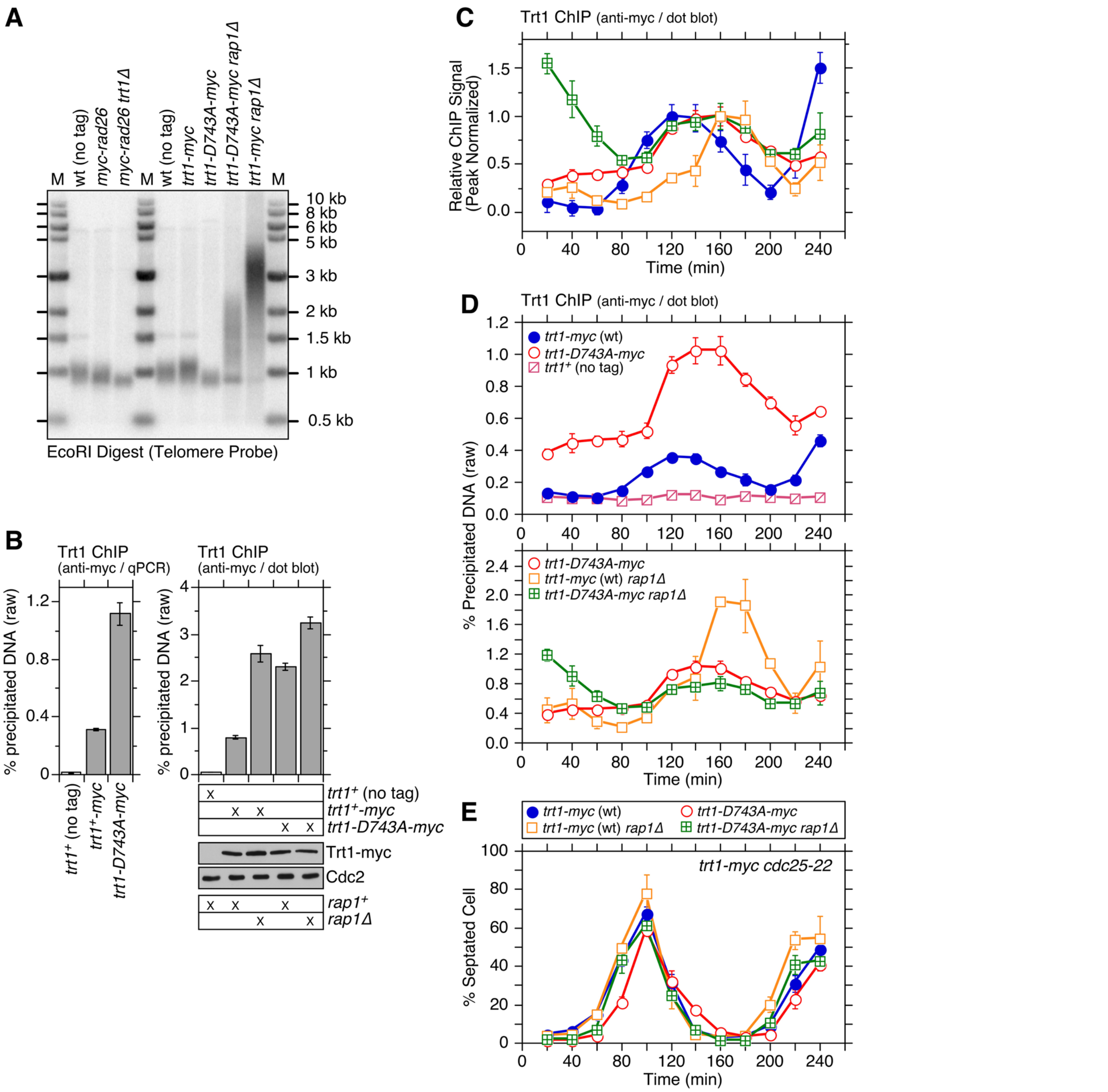

Figure S19 Cell cycle ChIP assays for catalytically dead Trt1-D743A. (**A**) Telomere length analysis for indicated strains used in ChIP analysis. Genomic DNA was prepared from early generation strains. After digestion with EcoRI, DNA was fractionated on a 1% agarose gel and processed for Southern blot analysis with a telomere probe. (**B**) Raw % precipitated DNA against input DNA for $Trt1^{TERT}$ obtained by real-time quantitative PCR analysis (left) or dot blot-based asynchronous ChIP assays with telomeric DNA probe (right). Trt1-D743A showed a statistically significant increase in telomere association compared to wt $Trt1^{TERT}$ ($p=5.4x10^{-5}$) for PCR-based ChIP assay, independently confirming our conclusion from telomere-length corrected dot blot-based ChIP assay (Figure 6B). Anti-myc western blot analysis indicated comparable expression levels of Trt1 in different genetic backgrounds. Cdc2 western blot served as a loading control. (**C**) Peak normalized cell cycle ChIP data for wt or catalytically dead $Trt1^{TERT}$ in *rap1*$^{+}$ or *rap1Δ* cells. (**D**) Raw data of dot blot-based cell cycle ChIP assays for $Trt1^{TERT}$, performed with *cdc25-22* synchronized cell cultures and telomeric DNA probe. (**E**) % septated cells were measured to monitor cell cycle progression of *cdc25-22* synchronized cell cultures for $Trt1^{TERT}$ ChIP assays. Error bars correspond to SEM.

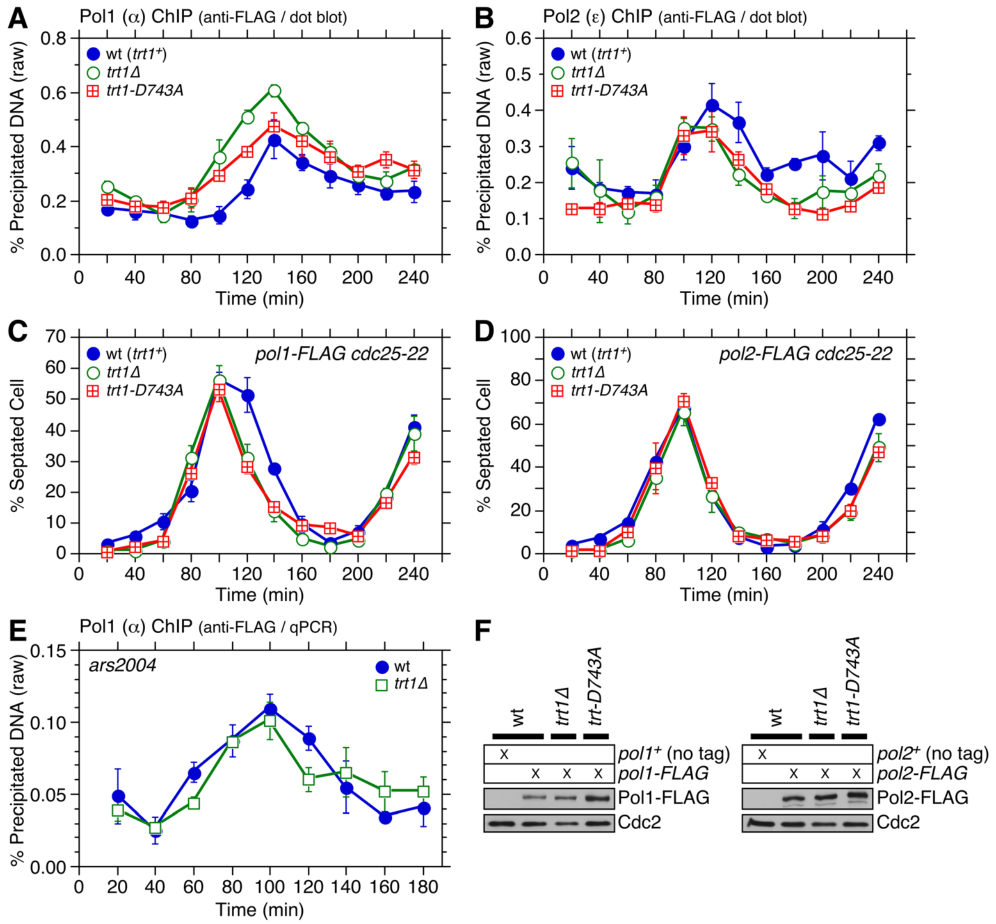

Figure S20 Cell cycle ChIP assays for DNA polymerases in *trt1* mutants. (**A**, **B**) Raw data of dot blot-based cell cycle ChIP assays for Pol1 (α) (A) and Pol2 (ε) (B), performed with *cdc25-22* synchronized cell cultures and telomeric DNA probe. (**C**, **D**) % septated cells were measured to monitor cell cycle progression of *cdc25-22* synchronized cell cultures for Pol1 (α) (C) and Pol2 (ε) (D) ChIP assays. (**E**) Pol1 (α) showed similar timing of recruitment to *ars2004* in wt and *trt1Δ* cells. Error bars correspond to SEM. (**F**) Anti-FLAG western blot analysis indicated comparable expression levels in different genetic backgrounds for both Pol1 and Pol2. Cdc2 western blot served as a loading control.

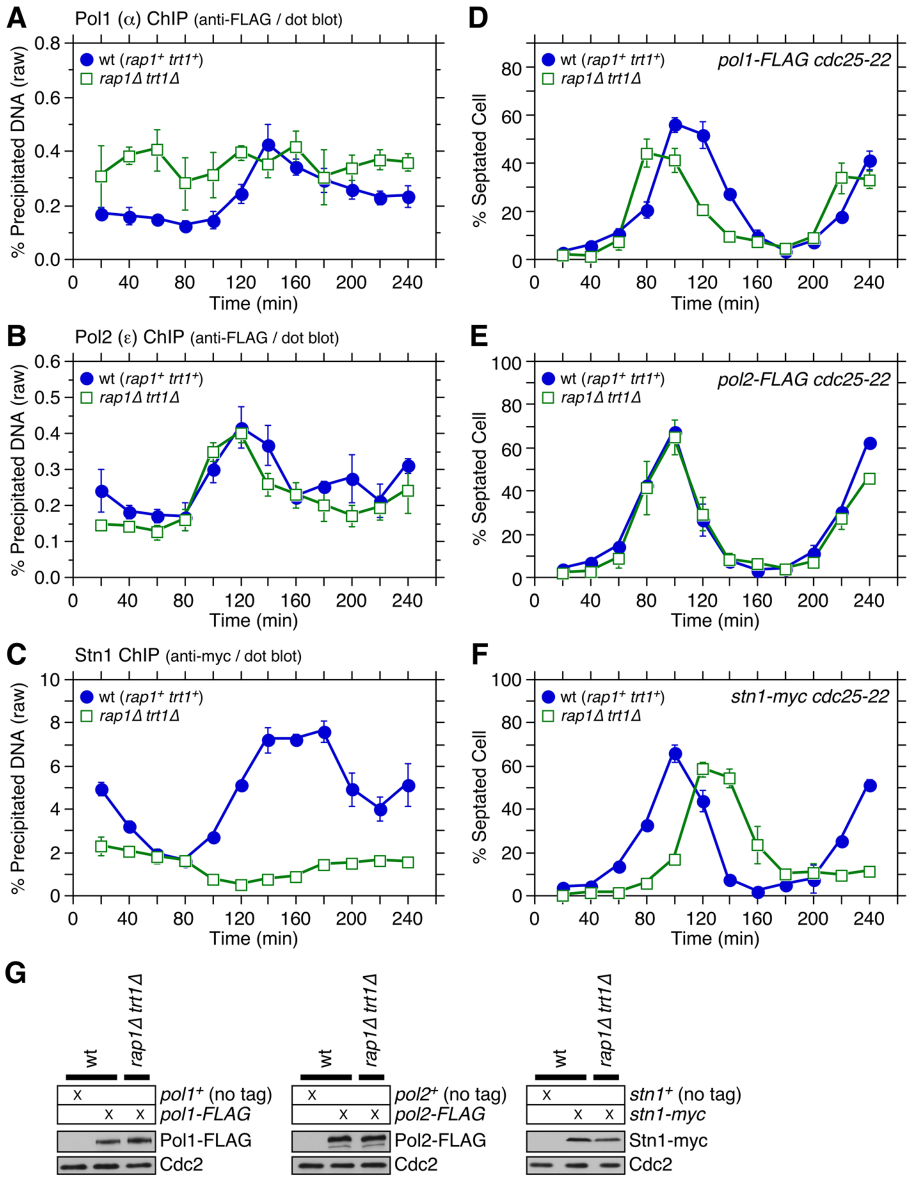

Figure S21 Cell cycle ChIP assays for DNA polymerases and Stn1 in *rap1Δ trt1Δ* cells. (**A**-**C**) Raw data of dot blot-based cell cycle ChIP assays for Pol1 (α) (A), Pol2 (ε) (B) and Stn1 (C), performed with *cdc25-22* synchronized cell cultures and telomeric DNA probe. (**D**-**F**) % septated cells were measured to monitor cell cycle progression of *cdc25-22* synchronized cell cultures for Pol1 (D), Pol2 (E) and Stn1 (F) ChIP assays. Error bars correspond to SEM. (**G**) Anti-FLAG (Pol1 and Pol2) and anti-myc (Stn1) western blot analyses indicated comparable expression levels in different genetic backgrounds for both Pol1 (α) and Pol2 (ε). Cdc2 western blot served as a loading control.

Supplementary Table S1 Telomere length correction factors (telomere/rDNA) for dot blot-based ChIP

Tagged protein	Genetic background	Correction factor[a]	Tagged protein	Genetic background	Correction factor[a]
Trt1-myc	wt	1.000±0.011 (n=23)	Rad11-FLAG	wt	1.000±0.026 (n=17)
	poz1Δ	7.523±0.235 (n=22)		*poz1Δ*	2.950±0.063 (n=17)
	rap1Δ	7.576±0.133 (n=23)		*rap1Δ*	6.281±0.152 (n=17)
	taz1Δ	6.507±0.085 (n=22)		*taz1Δ*	4.407±0.093 (n=15)
	trt1-D743A	1.277±0.158 (n=9)			
	trt1-D743A rap1Δ	2.409±0.190 (n=7)			
Pol1-FLAG	wt	1.000±0.013 (n=18)	Tpz1-myc	wt	1.000±0.014 (n=18)
	poz1Δ	2.474±0.044 (n=18)		*poz1Δ*	5.671±0.107 (n=18)
	rap1Δ	1.802±0.032 (n=18)		*rap1Δ*	6.229±0.154 (n=18)
	taz1Δ	1.001±0.027 (n=18)		*taz1Δ*	5.420±0.194 (n=18)
	trt1Δ	0.669±0.035 (n=9)			
	trt1-D743A	0.508±0.049 (n=9)			
	rap1Δ trt1Δ	0.146±0.009 (n=9)			
Pol2-FLAG	wt	1.000±0.020 (n=18)	Ccq1-myc	wt	1.000±0.020 (n=36)
	poz1Δ	5.389±0.146 (n=18)		*poz1Δ*	3.544±0.140 (n=33)
	rap1Δ	6.362±0.151 (n=15)		*rap1Δ*	5.061±0.216 (n=35)
	taz1Δ	5.008±0.087 (n=15)		*taz1Δ*	4.975±0.258 (n=35)
	trt1Δ	0.632±0.015 (n=9)			
	trt1-D743A	0.450±0.014 (n=9)			
	rap1Δ trt1Δ	0.832±0.016 (n=9)			
myc-Rad3	wt	1.000±0.019 (n=9)	Poz1-myc	wt	1.000±0.025 (n=26)
	poz1Δ	3.730±0.097 (n=9)		*rap1Δ*	4.773±0.311 (n=24)
	rap1Δ	4.790±0.114 (n=9)		*taz1Δ*	3.892±0.282 (n=27)
	taz1Δ	2.750±0.101 (n=9)			
myc-Rad26	wt	1.000±0.017 (n=18)	Stn1-myc	wt	1.000±0.021 (n=25)
	poz1Δ	6.842±0.132 (n=18)		*poz1Δ*	3.624±0.237 (n=27)
	rap1Δ	9.516±0.165 (n=18)		*rap1Δ*	5.416±0.364 (n=27)
	taz1Δ	6.105±0.124 (n=16)		*taz1Δ*	4.497±0.260 (n=26)
				rap1Δ trt1Δ	0.666±0.013 (n=9)

[a]Mean ± standard error of the mean. Values are normalized to wt cells with indicated tagged proteins. Number of samples used to determine correction factors are also indicated as (n=#).

Supplementary Table S2 Fission yeast strains used in this study

Figure		Strain	Full Genotype[a]
1B, S1	wt	TN2411	h^- his3-D1
	poz1Δ	YTC8489	h^- ade6-M216 his3-D1 poz1Δ::natMX6
	rap1Δ	YTC9370	h^+ his3-D1 rap1Δ::$ura4^+$
	taz1Δ	YTC9369	h^+ ade6-M216 his3-D1 taz1Δ::$ura4^+$
	poz1Δ rap1Δ	YTC9371	h^+ his3-D1 poz1Δ::natMX6 rap1Δ::$ura4^+$
	poz1Δ taz1Δ	YTC9372	h^+ ade6-M216 his3-D1 poz1Δ::natMX6 taz1Δ::$ura4^+$
	rap1Δ taz1Δ	YTC9373	h^+ ade6-M210 his3-D1 rap1Δ::$ura4^+$ taz1Δ::LEU2
	poz1Δ rap1Δ taz1Δ	YTC9374	h^+ his3-D1 poz1Δ::natMX6 rap1Δ::$ura4^+$ taz1Δ::LEU2
1C	$rap1^+$ (no tag)	TN2411	h^- his3-D1
	rap1-myc	YTC9493	h^+ ade6-M210 $rap1^+$-7myc::kanMX
	rap1-myc poz1Δ	YTC9929	h^- ade6-M210 his3-D1 $rap1^+$-7myc::kanMX poz1Δ::natMX6
	rap1-myc taz1Δ	YTC9934	h^+ ade6-M210 $rap1^+$-7myc::kanMX taz1Δ::$ura4^+$
	rap1-myc poz1Δ taz1Δ	YTC10060	h^- ade6-M210 his3-D1 $rap1^+$-7myc::kanMX poz1Δ::natMX6 taz1Δ::$ura4^+$
1D	$trt1^+$ (no tag)	SS5264	h^- his3-D1 cdc25-22
	trt1-myc	TN7708	h^- his3-D1 $trt1^+$-G_8-13myc::kanMX6 cdc25-22
	trt1-myc poz1Δ	YTC8558	h^- his3-D1 $trt1^+$-G_8-13myc::kanMX6 poz1Δ::natMX6 cdc25-22
	trt1-myc rap1Δ	YTC8969	h^- his3-D1 $trt1^+$-G_8-13myc::kanMX6 rap1Δ::$ura4^+$ cdc25-22
	trt1-myc taz1Δ	TN8601	h^- his3-D1 $trt1^+$-G_8-13myc::kanMX6 taz1Δ::$ura4^+$ cdc25-22
	trt1-myc poz1Δ taz1Δ	YTC9404	h^- his3-D1 $trt1^+$-G_8-13myc::kanMX6 poz1Δ::natMX6 taz1Δ::$ura4^+$ cdc25-22
	trt1-myc rap1Δ taz1Δ	YTC9411	h^- ade6-M210 his3-D1 $trt1^+$-G_8-13myc::kanMX6 rap1Δ::$ura4^+$ taz1Δ::LEU2 cdc25-22
	trt1-myc poz1Δ rap1Δ	YTC9453	h^- his3-D1 $trt1^+$-G_8-13myc::kanMX6 poz1Δ::natMX6 rap1Δ::$ura4^+$ cdc25-22
	trt1-myc poz1Δ rap1Δ taz1Δ	YTC9452	h^- his3-D1 $trt1^+$-G_8-13myc::kanMX6 poz1Δ::natMX6 rap1Δ::$ura4^+$ taz1Δ::LEU2 cdc25-22
2, 3C, 5,	trt1-myc	TN7708	h^- his3-D1 $trt1^+$-G_8-13myc::kanMX6 cdc25-22
7C, S2, S3,	trt1-myc poz1Δ	YTC8558	h^- his3-D1 $trt1^+$-G_8-13myc::kanMX6 poz1Δ::natMX6 cdc25-22
S5, S6,	trt1-myc rap1Δ	YTC8969	h^- his3-D1 $trt1^+$-G_8-13myc::kanMX6 rap1Δ::$ura4^+$ cdc25-22
S17, S18	trt1-myc taz1Δ	TN8601	h^- his3-D1 $trt1^+$-G_8-13myc::kanMX6 taz1Δ::$ura4^+$ cdc25-22
	pol1-FLAG	TN4781	h^- his3-D1 $pol1^+$-5FLAG::kanMX cdc25-22
	pol1-FLAG poz1Δ	YTC8705	h^- his3-D1 $pol1^+$-5FLAG::kanMX poz1Δ::natMX6 cdc25-22
	pol1-FLAG rap1Δ	YTC9589	h^- his3-D1 $pol1^+$-5FLAG::kanMX rap1Δ::$ura4^+$ cdc25-22
	pol1-FLAG taz1Δ	YTC9375	h^- his3-D1 $pol1^+$-5FLAG::kanMX taz1Δ::$ura4^+$ cdc25-22
	pol2-FLAG	TN4782	h^+ ade6-M210 his3-D1 $pol2^+$-5FLAG::kanMX cdc25-22
	pol2-FLAG poz1Δ	YTC8693	h^- ade6-M210 his3-D1 $pol2^+$-5FLAG::kanMX poz1Δ::natMX6 cdc25-22
	pol2-FLAG rap1Δ	YTC9423	h^- ade6-M210 his3-D1 $pol2^+$-5FLAG::kanMX rap1Δ::$ura4^+$ cdc25-22
	pol2-FLAG taz1Δ	YTC9280	h^- ade6-M210 his3-D1 $pol2^+$-5FLAG::kanMX taz1Δ::$ura4^+$ cdc25-22
	wt (no tag)	SS5264	h^- his3-D1 cdc25-22
	poz1Δ (no tag)	YTC8929	h^- his3-D1 poz1Δ::natMX6 cdc25-22
	rap1Δ (no tag)	YTC8938	h^+ his3-D1 rap1Δ::$ura4^+$ cdc25-22
	taz1Δ (no tag)	YTC8933	h^- his3-D1 taz1Δ::$ura4^+$ cdc25-22
3, S11	myc-rad26	TN7840	h^- his3-D1 9myc-$rad26^+$::hphMX6 cdc25-22
	myc-rad26 poz1Δ	YTC10349	h^- his3-D1 9myc-$rad26^+$::hphMX6 poz1Δ::natMX6 cdc25-22
	myc-rad26 rap1Δ	YTC10338	h^- his3-D1 9myc-$rad26^+$::hphMX6 rap1Δ::$ura4^+$ cdc25-22
	myc-rad26 taz1Δ	YTC10420	h^+ his3-D1 9myc-$rad26^+$::hphMX6 taz1Δ::$ura4^+$ cdc25-22
	rad11-FLAG	BAM5875	h^+ his3-D1 $rad11^+$-5FLAG::kanMX cdc25-22
	rad11-FLAG poz1Δ	YTC10334	h^+ his3-D1 $rad11^+$-5FLAG::kanMX poz1Δ::natMX6 cdc25-22
	rad11-FLAG rap1Δ	YTC10341	h^+ his3-D1 $rad11^+$-5FLAG::kanMX rap1Δ::$ura4^+$ cdc25-22
	rad11-FLAG taz1Δ	YTC10319	h^- his3-D1 $rad11^+$-5FLAG::kanMX taz1Δ::$ura4^+$ cdc25-22

4A	*ccq1-FLAG*	TN6847	*h$^-$ his3-D1 ccq1$^+$-5FLAG::kanMX6 cdc25-22*
	ccq1-FLAG rap1Δ	YTC10947	*h$^-$ ccq1$^+$-5FLAG::kanMX6 rap1Δ::ura4$^+$ cdc25-22*
	ccq1-FLAG taz1Δ	YTC10950	*h$^+$ his3-D1 ccq1$^+$-5FLAG::kanMX6 taz1Δ::ura4$^+$ cdc25-22*
4B, 5,	*ccq1-myc*	TN7456	*h$^-$ his3-D1 ccq1$^+$-13myc::kanMX6 cdc25-22*
S12-S15,	*ccq1-myc poz1Δ*	YTC8709	*h$^-$ his3-D1 ccq1$^+$-13myc::kanMX6 poz1Δ::natMX6 cdc25-22*
S17, S18	*ccq1-myc rap1Δ*	YTC9384	*h$^+$ his3-D1 ccq1$^+$-13myc::kanMX6 rap1Δ::ura4$^+$ cdc25-22*
	ccq1-myc taz1Δ	YTC9292	*h$^-$ his3-D1 ccq1$^+$-13myc::kanMX6 taz1Δ::ura4$^+$ cdc25-22*
	tpz1-myc	TN7467	*h$^-$ his3-D1 tpz1$^+$-13myc::kanMX6 cdc25-22*
	tpz1-myc poz1Δ	YTC9362	*h$^-$ his3-D1 tpz1$^+$-13myc::kanMX6 poz1Δ::natMX6 cdc25-22*
	tpz1-myc rap1Δ	YTC9307	*h$^-$ his3-D1 tpz1$^+$-13myc::kanMX6 rap1Δ::ura4$^+$ cdc25-22*
	tpz1-myc taz1Δ	YTC9327	*h$^-$ his3-D1 tpz1$^+$-13myc::kanMX6 taz1Δ::ura4$^+$ cdc25-22*
	poz1-myc	TN6843	*h$^-$ his3-D1 poz1$^+$-13myc::kanMX6 cdc25-22*
	poz1-myc rap1Δ	YTC9314	*h$^-$ his3-D1 poz1$^+$-13myc::kanMX6 rap1Δ::ura4$^+$ cdc25-22*
	poz1-myc taz1Δ	YTC9285	*h$^-$ his3-D1 poz1$^+$-13myc::kanMX6 taz1Δ::ura4$^+$ cdc25-22*
	stn1-myc	TN6886	*h$^-$ his3-D1 stn1$^+$-13myc::kanMX6 cdc25-22*
	stn1-myc poz1Δ	YTC8717	*h$^-$ his3-D1 stn1$^+$-13myc::kanMX6 poz1Δ::natMX6 cdc25-22*
	stn1-myc rap1Δ	YTC9299	*h$^-$ his3-D1 stn1$^+$-13myc::kanMX6 rap1Δ::ura4$^+$ cdc25-22*
	stn1-myc taz1Δ	YTC9414	*h$^+$ his3-D1 stn1$^+$-13myc::kanMX6 taz1Δ::ura4$^+$ cdc25-22*
6A, S19A	*rad26$^+$* (no tag)	TN2411	*h$^-$ his3-D1*
	myc-rad26	LS7680	*h$^-$ his3-D1 9myc-rad26$^+$::hphMX6*
	myc-rad26 trt1Δ	YTC12785	*h$^-$ ade6-M216 his3-D1 9myc-rad26$^+$::hphMX6 trt1Δ::his3$^+$*
6B-C, 7C,	*trt1$^+$* (no tag)	SS5264	*h$^-$ his3-D1 cdc25-22*
S19	*trt1-myc*	TN7708	*h$^-$ his3-D1 trt1$^+$-G_8-13myc::kanMX6 cdc25-22*
	trt1-myc rap1Δ	YTC8969	*h$^-$ his3-D1 trt1$^+$-G_8-13myc::kanMX6 rap1Δ::ura4$^+$ cdc25-22*
	trt1-D743A-myc	YTC12787	*h$^-$ ade6-M210 his3-D1 trt1-D743A-G_8-13myc::kanMX6 cdc25-22*
	trt1-D743A-myc rap1Δ	YTC12788	*h$^-$ ade6-M210 his3-D1 trt1-D743A-G_8-13myc::kanMX6 rap1Δ::ura4$^+$ cdc25-22*
7, S20	*pol1-FLAG trt1$^+$*	TN4781	*h$^-$ his3-D1 pol1$^+$-5FLAG::kanMX cdc25-22*
	pol1-FLAG trt1Δ	TN8388[b]	*h$^-$ ade6-M210 his3-D1 pol1$^+$-5FLAG::kanMX trt1Δ::his3$^+$ cdc25-22 //pNR210-trt1$^+$(ade6$^+$; P_{adh}::tk; trt1$^+$)*
	pol1-FLAG trt1-D743A	YTC10066[b]	*h$^-$ ade6-M210 his3-D1 pol1$^+$-5FLAG::kanMX trt1-D743A::LEU2 cdc25-22 //pNR210-trt1$^+$ (ade6$^+$; P_{adh}::tk; trt1$^+$)*
	pol2-FLAG trt1$^+$	TN4782	*h$^+$ ade6-M210 his3-D1 pol2$^+$-5FLAG::kanMX cdc25-22*
	pol2-FLAG trt1Δ	TN8393[b]	*h$^-$ ade6-M210 his3-D1 pol2$^+$-5FLAG::kanMX trt1Δ::his3$^+$ cdc25-22 //pNR210-trt1$^+$ (ade6$^+$; P_{adh}::tk; trt1$^+$)*
	pol2-FLAG trt1-D743A	YTC10064[b]	*h$^-$ ade6-M210 his3-D1 pol2$^+$-5FLAG::kanMX trt1-D743A::LEU2 cdc25-22 //pNR210-trt1$^+$ (ade6$^+$; P_{adh}::tk; trt1$^+$)*
8, S21	*pol1-FLAG*	TN4781	*h$^-$ his3-D1 pol1$^+$-5FLAG::kanMX cdc25-22*
	pol1-FLAG rap1Δ	YTC9589	*h$^-$ his3-D1 pol1$^+$-5FLAG::kanMX rap1Δ::ura4$^+$ cdc25-22*
	pol1-FLAG rap1Δ trt1Δ	BAM10118[b]	*h$^-$ ade6-M210 his3-D1 pol1$^+$-5FLAG::kanMX rap1Δ::ura4$^+$ trt1Δ::his3$^+$ cdc25-22 //pNR210-trt1$^+$ (ade6$^+$; P_{adh}::tk; trt1$^+$)*
	pol2-FLAG	TN4782	*h$^+$ ade6-M210 his3-D1 pol2$^+$-5FLAG::kanMX cdc25-22*
	pol2-FLAG rap1Δ	YTC9423	*h$^-$ ade6-M210 his3-D1 pol2$^+$-5FLAG::kanMX rap1Δ::ura4$^+$ cdc25-22*
	pol2-FLAG rap1Δ trt1Δ	BAM10114[b]	*h- ade6-M210 his3-D1 pol2$^+$-5FLAG::kanMX rap1Δ::ura4$^+$ trt1Δ::his3$^+$ cdc25-22 //pNR210-trt1$^+$ (ade6$^+$; P_{adh}::tk; trt1$^+$)*
	stn1-myc	TN6886	*h$^-$ his3-D1 stn1$^+$-13myc::kanMX6 cdc25-22*
	stn1-myc rap1Δ	YTC9299	*h$^-$ his3-D1 stn1$^+$-13myc::kanMX6 rap1Δ::ura4$^+$ cdc25-22*
	stn1-myc rap1Δ trt1Δ	YTC13543[b]	*h- ade6-M210 his3-D1 stn1$^+$-13myc::kanMX6 rap1Δ::ura4$^+$ trt1Δ::his3$^+$ cdc25-22 //pNR210-trt1$^+$ (ade6$^+$; P_{adh}::tk; trt1$^+$)*
	wt (no tag)	SS5264	*h$^-$ his3-D1 cdc25-22*

S4	wt	TN4777	h$^-$ leu1-32::[hENT1 leu1$^+$] his3-D1 his7-366::[hsv-tk his7+] cdc25-22
	poz1Δ	YTC9777	h$^-$ leu1-32::[hENT1 leu1$^+$] his3-D1 his7-366::[hsv-tk his7$^+$] poz1Δ::natMX6 cdc25-22
	rap1Δ	YTC6430	h$^-$ leu1-32::[hENT1 leu1$^+$] his3-D1 his7-366::[hsv-tk his7$^+$] rap1Δ::ura4$^+$ cdc25-22
	taz1Δ	YTC6479	h$^-$ leu1-32::[hENT1 leu1$^+$] his3-D1 his7-366::[hsv-tk his7$^+$] taz1Δ::ura4$^+$ cdc25-22
S7-S9	*trt1-myc*	TN7708	h$^-$ his3-D1 trt1$^+$-G_8-13myc::kanMX6 cdc25-22
	trt1-myc poz1Δ	YTC8558	h$^-$ his3-D1 trt1$^+$-G_8-13myc::kanMX6 poz1Δ::natMX6 cdc25-22
	trt1-myc rap1Δ	YTC8969	h$^-$ his3-D1 trt1$^+$-G_8-13myc::kanMX6 rap1Δ::ura4$^+$ cdc25-22
	trt1-myc taz1Δ	TN8601	h$^-$ his3-D1 trt1$^+$-G_8-13myc::kanMX6 taz1Δ::ura4$^+$ cdc25-22
	myc-rad26	TN7840	h$^-$ his3-D1 9myc-rad26$^+$::hphMX6 cdc25-22
	myc-rad26 poz1Δ	YTC10349	h$^-$ his3-D1 9myc-rad26$^+$::hphMX6 poz1Δ::natMX6 cdc25-22
	myc-rad26 rap1Δ	YTC10338	h$^-$ his3-D1 9myc-rad26$^+$::hphMX6 rap1Δ::ura4$^+$ cdc25-22
	myc-rad26 taz1Δ	YTC10420	h$^+$ his3-D1 9myc-rad26$^+$::hphMX6 taz1Δ::ura4$^+$ cdc25-22
	myc-rad3	YTC13163	h$^+$ his3-D1 9myc-rad3$^+$ cdc25-22
	myc-rad3 poz1Δ	YTC13047	h$^+$ his3-D1 9myc-rad3$^+$ poz1Δ::natMX6 cdc25-22
	myc-rad3 rap1Δ	YTC13053	h$^+$ his3-D1 9myc-rad3$^+$ rap1Δ::ura4$^+$ cdc25-22
	myc-rad3 taz1Δ	YTC13057	h$^+$ his3-D1 9myc-rad3$^+$ taz1Δ::ura4$^+$ cdc25-22
	rad11-FLAG	BAM5875	h$^+$ his3-D1 rad11$^+$-5FLAG::kanMX cdc25-22
	rad11-FLAG poz1Δ	YTC10334	h$^+$ his3-D1 rad11$^+$-5FLAG::kanMX poz1Δ::natMX6 cdc25-22
	rad11-FLAG rap1Δ	YTC10341	h$^+$ his3-D1 rad11$^+$-5FLAG::kanMX rap1Δ::ura4$^+$ cdc25-22
	rad11-FLAG taz1Δ	YTC10319	h$^-$ his3-D1 rad11$^+$-5FLAG::kanMX taz1Δ::ura4$^+$ cdc25-22
	tpz1-myc	TN7467	h$^-$ his3-D1 tpz1$^+$-13myc::kanMX6 cdc25-22
	tpz1-myc poz1Δ	YTC9362	h$^-$ his3-D1 tpz1$^+$-13myc::kanMX6 poz1Δ::natMX6 cdc25-22
	tpz1-myc rap1Δ	YTC9307	h$^-$ his3-D1 tpz1$^+$-13myc::kanMX6 rap1Δ::ura4$^+$ cdc25-22
	tpz1-myc taz1Δ	YTC9327	h$^-$ his3-D1 tpz1$^+$-13myc::kanMX6 taz1Δ::ura4$^+$ cdc25-22
	ccq1-myc	TN7456	h$^-$ his3-D1 ccq1$^+$-13myc::kanMX6 cdc25-22
	ccq1-myc poz1Δ	YTC8709	h$^-$ his3-D1 ccq1$^+$-13myc::kanMX6 poz1Δ::natMX6 cdc25-22
	ccq1-myc rap1Δ	YTC9384	h$^+$ his3-D1 ccq1$^+$-13myc::kanMX6 rap1Δ::ura4$^+$ cdc25-22
	ccq1-myc taz1Δ	YTC9292	h$^-$ his3-D1 ccq1$^+$-13myc::kanMX6 taz1Δ::ura4$^+$ cdc25-22
	poz1-myc	TN6843	h$^-$ his3-D1 poz1$^+$-13myc::kanMX6 cdc25-22
	poz1-myc rap1Δ	YTC9314	h$^-$ his3-D1 poz1$^+$-13myc::kanMX6 rap1Δ::ura4$^+$ cdc25-22
	poz1-myc taz1Δ	YTC9285	h$^-$ his3-D1 poz1$^+$-13myc::kanMX6 taz1Δ::ura4$^+$ cdc25-22
	stn1-myc	TN6886	h$^-$ his3-D1 stn1$^+$-13myc::kanMX6 cdc25-22
	stn1-myc poz1Δ	YTC8717	h$^-$ his3-D1 stn1$^+$-13myc::kanMX6 poz1Δ::natMX6 cdc25-22
	stn1-myc rap1Δ	YTC9299	h$^-$ his3-D1 stn1$^+$-13myc::kanMX6 rap1Δ::ura4$^+$ cdc25-22
	stn1-myc taz1Δ	YTC9414	h$^+$ his3-D1 stn1$^+$-13myc::kanMX6 taz1Δ::ura4$^+$ cdc25-22
	wt (no tag)	SS5264	h$^-$ his3-D1 cdc25-22
	poz1Δ (no tag)	YTC8929	h$^-$ his3-D1 poz1Δ::natMX6 cdc25-22
	rap1Δ (no tag)	YTC8938	h$^+$ his3-D1 rap1Δ::ura4$^+$ cdc25-22
	taz1Δ (no tag)	YTC8933	h$^-$ his3-D1 taz1Δ::ura4$^+$ cdc25-22
S10A, C, D	*tel1*$^+$ (no tag)	SS5264	h$^-$ his3-D1 cdc25-22
	myc-tel1	YTC13049	h$^+$ ade6-704 his3-D1 9myc-tel1$^+$ cdc25-22
	myc-tel1	LS8284	h$^-$ ade6-704 his3-D1 9myc-tel1$^+$
	poz1Δ (no tag)	YTC8930	h$^+$ his3-D1 poz1Δ::natMX6 cdc25-22
	myc-tel1 poz1Δ	YTC10940	h$^-$ ade6-704 his3-D1 9myc-tel1$^+$ poz1Δ::natMX6 cdc25-22
	rap1Δ (no tag)	YTC8938	h$^+$ his3-D1 rap1Δ::ura4$^+$ cdc25-22
	myc-tel1 rap1Δ	YTC10935	h$^+$ ade6-704 his3-D1 9myc-tel1$^+$ rap1Δ::ura4$^+$ cdc25-22
	taz1Δ (no tag)	YTC8933	h$^-$ his3-D1 taz1Δ::ura4$^+$ cdc25-22
	myc-tel1 taz1Δ	YTC10932	h$^+$ ade6-704 his3-D1 9myc-tel1$^+$ taz1Δ::ura4$^+$ cdc25-22
S10B, D	*tel1*$^+$ (no tag)	TN2411	h$^-$ his3-D1
	myc-tel1	LS8284	h$^-$ ade6-704 his3-D1 9myc-tel1$^+$
	rap1Δ (no tag)	TN5346	h$^+$ his3-D1 rap1Δ::ura4$^+$

myc-tel1 rap1Δ	YTC10938	*h^- his3-D1 9myc-tel1$^+$ rap1Δ::ura4$^+$*
rad3-kdΔ (no tag)	TN1678	*h^- ade6-M216 his3-D1 rad3-kdΔ::kanMX4*
myc-tel1 rad3-kdΔ	YTC12932	*h^- ade6-704 his3-D1 9myc-tel1$^+$ rad3-kdΔ::kanMX4*
rad3-kdΔ rap1Δ (no tag)	YTC13045	*h^+ ade6$^-$ his3-D1 rad3-kdΔ::kanMX4 rap1Δ::ura4$^+$*
myc-tel1 rad3-kdΔ rap1Δ	YTC12931	*h^+ ade6-704 his3-D1 9myc-tel1$^+$ rad3-kdΔ::kanMX4 rap1Δ::ura4$^+$*

[a]All strains are *leu1-32 ura4-D18*, except for strains expressing hENT1, which are *leu1-32::[hENT1 leu1$^+$] ura4-D18.*

[b]Early generation strains that have just lost *trt1$^+$* plasmid (*pNR210-trt1$^+$*) were used to ensure that ChIP assays monitored *trt1Δ* or *trt1-D743A* cells carrying longest telomeres as possible.

Supplementary Table S3 DNA primers used in this study

Primer Name	Primer Sequence (5' to 3')	Description
jk380	TATTTCTTTATTCAACTTACCGCACTTC	Used as real-time PCR primers for telomere ChIP [63].
jk381	CAGTAGTGCAGTGTATTATGATAATTAAAATGG	
ars2004-66-F	CGGATCCGTAATCCCAACAA	Used as real-time PCR primers for *ars2004* ChIP [64].
ars2004-66-R	TTTGCTTACATTTTCGGGAACTTA	
BAM140	TTTTCAGGGTCGGTAGAGTCAGAG	Used to PCR amplify a region near *ars3001* within rDNA repeats. The PCR product was used as template to generate rDNA region probe to establish telomere correction factors.
BAM141	CCTCCTTACTTCTCCTTATTCCACG	
trt1-B29	CTTATTCTAAATGAAAGGAGATTAGC	Used to PCR amplify *trt1-*G_8*-13myc::kanMX6* construct to generate cells carrying *trt1-D743A-*G_8*-13myc::kanMX6* allele.
trt1-T30	TAGGCTAGGATACTCTATGTGTATGAGAGC	
UraKan-T1	CCCACTGGCTATATGTATGCATTTGTGTTAAAAAAGTTTGTATAGATTATTTAATCTACTCAGCATTCTTTCTCTAACGCGCCAGATCTGTTTAGCTTGC	Used to swap marker from *ura4*$^+$ to *kanMX4*. 5' end anneals to *ura4*$^+$ and 3' end (underlined) anneals to *kanMX4* module in pFA6a-kanMX4 plasmid.
UraKan-B1	GATATTGACGAAACTTTTTGACATCTAATTTATTCTGTTCCAACACCAATGTTTATAACCAAGTTTTATCTTGTTTGGGCGGCGTTAGTATCGAATCGAC	

Supplementary Table S4 Plasmids used in this study

Plasmid (Lab stock #)	Genes	Description
pTELO (254)	Fission yeast telomere fragment; *ampR*	Carries a telomeric repeat fragment (ApaI-SacI) used in generating a telomere probe for Southern blot analysis [54].
pNR210-trt1$^+$ (290)	*ade6*$^+$; *trt1*$^+$; P_{adh}*::tk*; *ampR*	Trt1 plasmid used to maintain telomeres. It also expresses *tk* (herpes simplex virus thymidine kinase) gene to allow counter selection of the plasmid on media containing 5FdUR [65].
pFA6a-kanMX4 (4)	*kanMX4*; *ampR*	Used as a PCR template for *kanMX4* [55] to swap marker to generate *rad3-kdΔ::kanMX4* strain.
pTM580 (461)	Full length *GAD-tpz1*; *LEU2*; *ampR*	pGAD-GH-Tpz1 full length plasmid from Ishikawa lab [6].
pGAD-GH-tpz1$_{2-420}$ (838)	*GAD-tpz1*$_{2-420}$; *LEU2*; *ampR*	Expresses truncated GAL4 AD-Tpz1 (2-420).
pGAD-GH-tpz1$_{224-508}$ (814)	*GAD-tpz1*$_{224-508}$; *LEU2*; *ampR*	Expresses truncated GAL4 AD-Tpz1 (224-508).
pGAD-GH-tpz1$_{224-420}$ (818)	*GAD-tpz1*$_{224-420}$; *LEU2*; *ampR*	Expresses truncated GAL4 AD-Tpz1 (224-420).
pGAD-GH-tpz1$_{2-223}$ (812)	*GAD-tpz1*$_{2-223}$; *LEU2*; *ampR*	Expresses truncated GAL4 AD-Tpz1 (2-223).
pGAD-GH-tpz1$_{421-508}$ (816)	*GAD-tpz1*$_{421-508}$; *LEU2*; *ampR*	Expresses truncated GAL4 AD-Tpz1 (421-508).
pGBKT7-stn1 (476)	*DBD-stn1*; *TRP1*; *kanR*	Expresses GAL4 DNA BD-Stn1.
pGBKT7-ten1 (474)	*DBD-ten1*; *TRP1*; *kanR*	Expresses GAL4 DNA BD-Ten1.
pGBKT7-stn1+ten1 (570)	*DBD-stn1*; *ten1*; *TRP1*; *kanR*	Expresses GAL4 DNA BD-Stn1 and wt Ten1. (Not fused to either GAL4 DNA BD or GAL4 AD.)
